# ADAPT-M: a workflow for rapid, quantitative in vitro measurements of enriched protein libraries

**DOI:** 10.1038/s41467-026-75463-1

**Published:** 2026-07-15

**Authors:** Carla P. Perez, Nicole V. DelRosso, Cameron L. Noland, Udit Parekh, Christian A. Choe, Raphael R. Eguchi, Qi Wen, Polly M. Fordyce, Po-Ssu Huang

**Affiliations:** 1 Biophysics Program, Stanford University, Stanford, CA USA; 2 Department of Cellular & Molecular Pharmacology, University of California, San Francisco, CA USA; 3 Discovery Chemistry, Merck & Co. Inc., South San Francisco, CA USA; 4 Department of Bioengineering, Stanford University, Stanford, CA USA; 5 Department of Biochemistry, Stanford University, Stanford, CA USA; 6 Sarafan ChEM-H Institute, Stanford University, Stanford, CA USA; 7 Chan Zuckerberg Biohub, San Francisco, CA USA; 8 Department of Genetics, Stanford University, Stanford, CA, USA

**Keywords:** High-throughput screening, Protein design

## Abstract

Protein-protein interactions underpin most cellular processes, and engineered binders present powerful tools for probing biology and developing novel therapeutics. However, scalable, quantitative characterization of large numbers of candidates remains a major bottleneck. Here we show that ADAPT-M (**A**ffinity **D**etermination by **A**daptation of **P**ro**T**ein binders for **M**icrofluidics) enables rapid, parallel measurement of binding affinities and dissociation behavior directly from enriched display libraries in under one week, without requiring gene synthesis or hands-on protein purification. Applied to a computationally designed library targeting the SARS-CoV-2 Omicron BA.1 receptor binding domain, ADAPT-M recovered most highly enriched variants and revealed that many display-enriched binders lacked measurable binding in vitro, highlighting limitations of screening alone. ADAPT-M enabled quantitative characterization of dozens of binders in parallel and selection of lead candidates for structural analysis. Unexpectedly, structural and mutational studies revealed that designed binding interfaces were preserved despite engaging alternative epitopes. By bridging screening and scalable in vitro validation, ADAPT-M accelerates protein binder discovery and supports data-driven protein engineering.

## Abstract (web summary)

ADAPT-M is a workflow combining design and high-throughput experimentation. It overcomes the testing bottleneck and enables rapid quantitative affinity measurements of thousands of designer proteins enriched from yeast surface display libraries.

## Introduction

Protein-protein interactions underlie nearly all cellular processes, from cell signaling and communication to gene expression and immune responses. Understanding and engineering these interactions is fundamental to efforts in biotechnology, synthetic biology, and therapeutic development. In particular, with the advancements seen with machine learning algorithms, which are capable of rapidly generating new designer proteins, there is an ever-increasing demand for high-throughput and quantitative experimental validation methods. Display technologies such as yeast surface display^1^ (YSD) allow protein engineers to screen large libraries of designed or randomly mutated protein variants in high throughput to select for candidates binding to a target of interest. These platforms provide relative binding information across pooled variants, but not precise biophysical parameters^1,2^. Methods such as TITE-seq^3^ and MAGMA-seq^4^ couple YSD with next-generation sequencing (NGS) and fit titration curves across multiple antigen concentrations to extract quantitative affinities. While powerful, these methods can be labor- and reagent-intensive, especially for weaker or transient interactions. AlphaSeq^5^, an alternative display-based strategy, leverages synthetic yeast mating agglutination to enable library-on-library affinity profiling, using calibration to internal standards to estimate individual dissociation constants (*K*_d_s). However, this method necessitates that both protein partners be compatible with YSD, which can be limiting since large proteins, membrane-bound proteins, or proteins requiring specific glycosylation patterns may not functionally display on yeast^6^. In all cases, observed YSD enrichments convolve impacts of protein abundance (which depends on cell-specific differences in folding, degradation, and export), potential avid interactions with other surface-displayed proteins, post-translational modifications, and interaction affinities. As a result, in vitro characterization of hits remains essential to distinguish true binders, quantify affinities, rule out artifacts, and guide downstream development.

Traditional in vitro approaches for binding quantification, such as surface plasmon resonance^7^ (SPR), biolayer interferometry^8,9^ (BLI), or isothermal titration calorimetry^10^ (ITC), have historically been low-throughput, typically requiring purified recombinant proteins, which are often labor-intensive to produce. Recent advancements have improved the scalability of these techniques. For example, advancements in instrumentation have increased throughput by enabling larger numbers of samples to be measured in parallel (e.g. plate-based systems such as the Octet RED384 and Biacore 8k+), and coupling of these techniques to cell-free expression allows binding measurements to be performed directly on unpurified expression reactions^11,12^. However, these approaches require significant amounts of reagents (e.g. purified target protein to distribute across every well and expensive store-bought or challenging-to-prepare house-made cell-free reaction mixtures), and rely on gene synthesis of DNA templates for expression, which can be cost-prohibitive for large sequence libraries. As a result, only a subset of candidate sequences can be finely characterized, limiting insight into sequence-function relationships and the availability of quantitative affinity data for training predictive models.

STAMMPPING^13^, a fluorescent-based microfluidic platform, which leverages cell-free expression and pneumatically controlled valves to enable parallel, quantitative *K*_d_ measurements of protein-protein interactions using a low-volume microfluidic device, addresses many of these challenges. This method uses a C-terminal meGFP tag on immobilized binders to quantify expression levels and selectively pull down expressed proteins, thereby normalizing for sequence-dependent differences in expression and enabling high-throughput purification of protein libraries. These features make STAMMPPING a low-volume, scalable solution for directly characterizing large numbers of enriched candidates in vitro, making it feasible to profile the full range of diverse hits from a binder generation campaign.

**Fig. 1:**
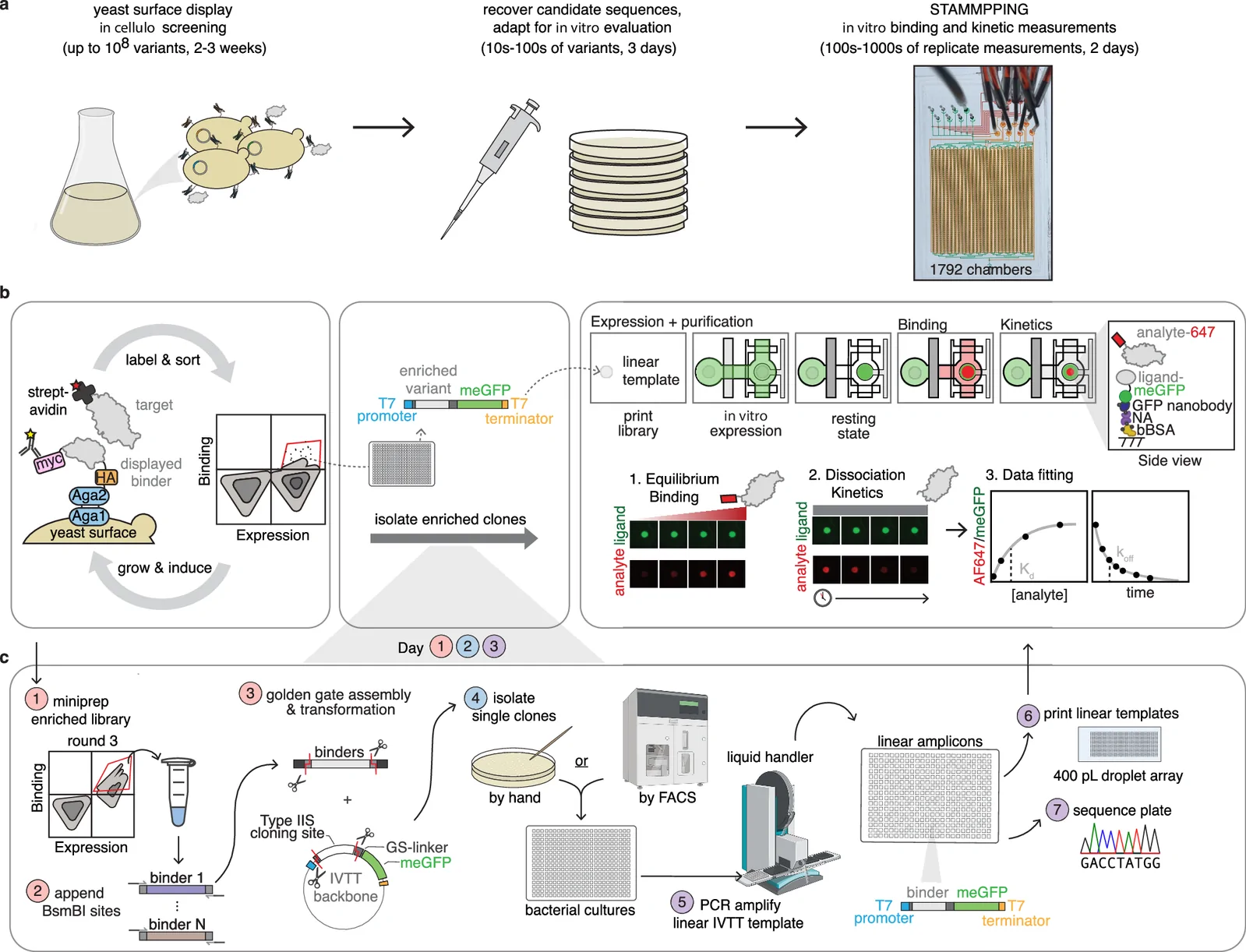
100s–1000s of affinity and kinetic measurements from enriched yeast surface display libraries in under a week. **a** Cartoon schematic illustrating the ADAPT-M workflow, starting with *in cellulo* screening in yeast, followed by recovery and adaptation of enriched DNA sequences, and concluding with in vitro evaluation on a microfluidic device via STAMMPPING^13^. Image of the microfluidic device reused from Horton et al., Short tandem repeats bind transcription factors to tune eukaryotic gene expression, 10.1126/science.add1250 [2023], AAAS^29^. **b** Detailed schematic of yeast surface display (left). Designed binders are displayed on the yeast surface as Aga2 fusions with a C-terminal myc tag. Labeling with an anti-cmyc antibody permits separation of expressing and non-expressing populations during flow cytometry; fluorescently labeled target proteins discriminate between binding and non-binding populations. Binding and expressing populations are sorted, and the labeling and sorting process is repeated over multiple rounds to enrich for binders; DNA sequences from an enriched population are isolated, meGFP-tagged, adapted for cell-free expression, and printed for the STAMMPPING assay (right)^13^. NA = NeutrAvidin, bBSA = biotinylated bovine serum albumin. **c** Detailed schematic of ADAPT-M workflow: (1) following a few rounds of yeast surface display, the enriched population is miniprepped; (2) the binder pool is amplified and BsmBI-v2 recognition sites are appended via PCR; (3) amplicons are assembled into a cell-free expression-compatible backbone via Golden Gate Assembly and then transformed into bacteria; (4) individual clones are isolated by colony picking or by cell sorting into individual wells of a 384-well plate; (5) overnight liquid cultures are used as templates for PCR amplification of cell-free expression-compatible amplicons; (6) amplicons are diluted and printed in array format on a glass slide and STAMMPPING begins; and (7) the entire amplicon-containing plate is sequenced to map wells to protein variant identity. Instrument icons created in BioRender. Perez, C. (2026) (https://BioRender.com/qbimhg2, https://BioRender.com/z63vlnn).

Here, we introduce and demonstrate “ADAPT-M” (**A**ffinity **D**etermination by **A**daptation of **P**ro**T**ein binders for **M**icrofluidics), a workflow to adapt enriched protein binders from YSD screening for in vitro evaluation via STAMMPPING (Fig. 1a). By leveraging DNA sequences directly recovered from enriched yeast populations, ADAPT-M eliminates the need for large-scale gene synthesis of individual candidates, substantially reducing the time and cost to profile binder libraries (Supplementary Tables 1, 2). Our workflow provides a streamlined bridge between candidates enriched via *in cellulo* screening and in vitro measurement of affinity and kinetic constants for hundreds of putative protein binders against a shared protein target in under a week. To demonstrate the utility of the workflow, we apply ADAPT-M to a library of computationally designed binders enriched via YSD, screening 51 unique putative binders in parallel. STAMMPPING measurements identified 14 true binders, for which we obtained quantitative binding affinities and qualitative kinetic profiles. We selected 2 candidates for detailed characterization, including paratope and epitope mapping, using STAMMPPING, YSD, and cryo-electron microscopy (cryo-EM). By enabling parallel, quantitative evaluation of a broad set of enriched binders, rather than just a select few, ADAPT-M expedites and expands the scope of protein engineering campaigns and design-build-test cycles.

## Results

### Overview of ADAPT-M workflow

ADAPT-M provides a pipeline to bridge pooled library screening with individual quantitative affinity measurements over 3 main stages: (1) YSD-based screening to identify candidate binders, (2) isolation and re-cloning of individual candidate binders into STAMMPPING-compatible constructs containing a C-terminal meGFP tag, and (3) STAMMPPING-based in vitro measurement of binding affinities (Fig. 1a). During Stage 1, several rounds of YSD enrich a binding population with varied affinity levels (Fig. 1b, left). Stage 2 transfers enriched variants to plasmids compatible with cell-free expression and isolates individual binder variants in unique wells of a 384-well plate (Fig. 1b, center). Stage 3 uses STAMMPPING to express, purify, and measure up to 1792 target interactions in parallel on a single microfluidic device (Fig. 1b, right). This workflow makes it possible to generate hundreds to thousands of in vitro affinity and kinetics measurements from enriched YSD libraries in <1 week.

We first enriched candidate binders using YSD. Initial identification of candidate binders from libraries containing up to 1 × 10^7^ members with YSD requires 14+ days (Fig. 1b, left). During the first 5 days, the library is cloned into a YSD-compatible vector and transformed into yeast cells as fusions to a surface anchor protein, Aga2, permitting surface display of the library upon induction. During the next 7+ days, the library is induced, and yeast cells are subjected to iterative rounds of fluorescence-activated cell sorting (FACS) to select and enrich a target-binding population. After enrichment, input and enriched populations are sequenced by NGS.

Following enrichment, we prepared candidate binders for individual characterization, shifting from pooled screening to individual measurements. This involves re-cloning variants carried in a yeast surface display-optimized plasmid into a plasmid optimized for cell-free expression and fluorescent detection with STAMMPPING. These binder sequences are then physically isolated from one another over 3 days (Fig. 1c). On Day 1, the enriched library is re-cloned into cell-free expression-compatible plasmids encoding expression of C-terminally meGFP-tagged variants via Golden Gate assembly. On Day 2, individual variant DNA sequences encoding each construct are isolated by either manually picking single bacterial colonies or by FACS sorting single cells into unique wells of a 384-well plate and incubating overnight (Supplementary Figs. 1, 2). On Day 3, linear templates encoding cell-free expression of individual variants are amplified via bacterial colony PCR and then arrayed on glass slides to begin the STAMMPPING pipeline. Isolated DNA variants are used directly for STAMMPPING without identifying the sequence of each clone; downstream sequencing of each template by either Sanger sequencing, short-read NGS^14,15^, or long-read sequencing^16^ allows subsequent identification of individual variants in parallel to STAMMPPING measurements.

Finally, we quantified affinities for all isolated binders using STAMMPPING, which requires 2–3 days. STAMMPPING takes place via 4 main steps: (1) polydimethylsiloxane (PDMS) devices containing pneumatic sandwich, neck, and button valves (Supplementary Fig. 3) are aligned and bonded to glass slides bearing printed DNA arrays (Fig. 1b, right); (2) device surfaces are patterned with anti-eGFP nanobodies in the center of each chamber and passivated with BSA elsewhere; (3) meGFP-tagged candidate binders are expressed within each chamber on-chip using PURExpress, a cell-free transcription-translation system, and immobilized via anti-eGFP nanobody-mediated capture for on-chip purification; and (4) increasing concentrations of fluorescently labeled target protein are introduced and mechanically “trapped” at equilibrium via button valves prior to washing and imaging. Small amounts of reagents are used (e.g. 5 µL of biotinylated anti-eGFP nanobody and 50 µL of PurExpress per device) by virtue of the low-volume microfluidic device with nanoliter sized chambers. Measured intensity ratios (target/binder) from images are then plotted as a function of target protein concentration and fit to quantify interaction affinities. To measure dissociation kinetics after the binding series, valves are iteratively opened to permit dissociation, closed to trap remaining bound labeled target protein, and chambers across the device are washed and imaged.

### ADAPT-M recovers enriched variants

**Fig. 2:**
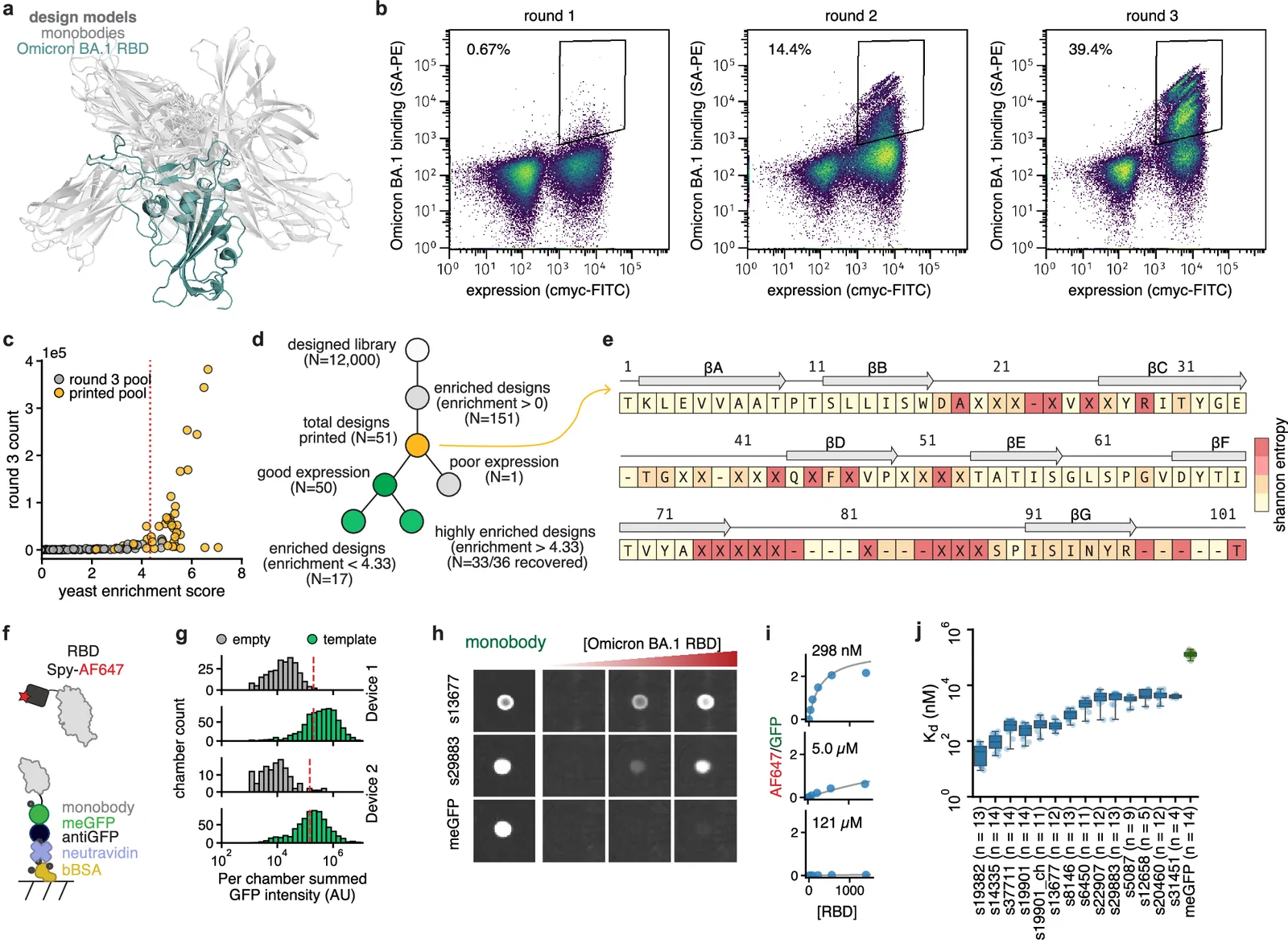
100 s of affinity measurements from an enriched yeast surface display library of designed monobodies against the SARS-CoV-2 Omicron/BA.1 receptor binding domain (RBD). **a** Structural models of 16 representative monobody designs (light gray) bound to the Omicron/BA.1 RBD (teal). **b** Flow cytometry dot plots showing enrichment across rounds of yeast display selection; sort gates are outlined in black. **c** Scatter plot of round 3 counts versus enrichment scores for 151 binders (enrichment score > 0). Binders recovered for STAMMPPING are shown in yellow; red dashed line indicates the threshold for highly enriched designs. **d** Binary tree summarizing outcomes for all 12,000 library members. **e** Sequence alignment to the monobody framework. Secondary structure is shown above, with beta strands (**a**–**g**) as arrows and loops as connecting lines. “X” denotes unseeded positions and “–” denotes gaps. Amino acids are colored by Shannon entropy across sequences printed for in vitro characterization. **f** Components of the functionalized device surface, including biotinylated bovine serum albumin (bBSA), NeutrAvidin (NA), and a biotinylated anti-GFP nanobody that binds the monobody-monomeric enhanced GFP (meGFP), and the SpyTag/Catcher-647-labeled Omicron/BA.1 RBD antigen. **g** Distribution of measured intensities for empty and template-containing chambers denoting per-chamber expression. Red dotted lines represent cutoff thresholds (10-16 standard deviations above the mean of empty chambers) used to differentiate between expressing and non-expressing chambers. **h** Representative images of three individual reaction chambers, depicting immobilized designs after expression and purification (left) and following incubation with increasing concentrations of the Omicron RBD target (right). **i** Fits (solid line) to normalized RBD/monobody fluorescence intensity ratios (points) as a function of free RBD concentration (Methods). **j** Measured *K*_d_s for enriched designs with binding above the limit of detection, variable number of replicates per design. Box plots show the median, interquartile range (25th–75th percentiles), and whiskers extending to 1.5× IQR. Source data are provided as a Source Data file.

Monobodies are small synthetic binding proteins derived from the immunoglobulin-like fibronectin domain^17^, and their compact size and lack of disulfides make them especially suitable for intracellular and therapeutic applications. Each monobody protein is composed of 7 beta strands (A-G) and 6 loops, with loops BC, DE, and FG structurally analogous to the CDR1, CDR2, and CDR3 loops of immunoglobulin heavy chain variable domains^17^. Rationally-designed monobodies typically vary these loop sequences to engage convex target surfaces, while variation of surface residues on beta strands C and D, in combination with loop sequences are modified to complement more concave surfaces. Here, we applied ADAPT-M to a library of 12,000 designed monobodies targeting the severe acute respiratory syndrome coronavirus 2 (SARS-CoV-2) Omicron/BA.1 variant Receptor Binding Domain (RBD), a highly divergent variant of concern that emerged during the COVID-19 pandemic^18^. Each designed protein was 91 amino acids long (enabling library synthesis as a 300 bp nucleotide oligo pool), and designs spanned a wide range of binding orientations relative to the Omicron RBD, including “loop-only” and “loop-side” recognition modes^19^ (Fig. 2a).

To identify designs that bound the target RBD, we cloned the library into a YSD vector (11,995/12,000 sequences recovered) and performed 3 rounds of FACS and outgrowth (Fig. 2b and Supplementary Fig. 4). To maximize the dynamic range of affinities within the captured binder population, we modified 2 steps of typical binder enrichment workflows^2^: (1) we performed each round of FACS using the same high staining concentration of Omicron RBD, and (2) we maintained the same permissive FACS gating window throughout all 3 rounds of sorting. After FACS sorting, 152 variants were enriched (as assessed by computing log enrichment scores^20^ comparing variant read counts between the initial and enriched pools); 36 of these were ‘highly enriched’ (defined as having a log enrichment score > 1 standard deviation above the mean of all designs with log enrichment score > 0) (Fig. 2c).

After selection, we used the ADAPT-M pipeline to re-clone and physically isolate enriched variants prior to STAMMPPING measurements. After individually inoculating 274 wells of a 384-well plate by hand, all inoculated wells showed successful outgrowth. Sequencing after STAMMPPING experiments revealed sampling of 51 unique monobody sequences. To assess the number of enriched sequences recovered by ADAPT-M, NGS sequencing analysis of input and enriched YSD populations was performed, revealing a recovery rate of 92% (33/36) for the 36 highly enriched sequences (Fig. 2d and Supplementary Table 3). This type of quality control is essential to ensure variants of interest are adequately represented by the ADAPT-M protocol and to identify missing variants that can be ordered and screened separately or during replicate STAMMPPING measurements.

As expected, enriched monobody sequences varied most significantly in loop regions, with notable variation in the length of the FG loop of designed monobodies, as well as in the surface-exposed residues of βC and βD (Fig. 2e). The diversity of loop sequences and conserved core observed in our selected designs align both with the sequence variability of existing monobodies in the Protein Data Bank (PDB) and our design strategy, which seeded monobody backbones with a constant framework sequence while redesigning residues at the binding interface.

### STAMMPPING in vitro affinity measurements

Next, we used STAMMPPING to quantify affinities between these designed monobodies and their Omicron RBD target. Fluorescence images taken after on-chip monobody expression revealed high-intensity spots corresponding to surface-immobilized meGFP-tagged proteins with intensity distributions substantially higher than those of empty chambers (Fig. 2f–h); overall, 31/34 printed designs and an meGFP-only negative control expressed above the expression threshold (defined here as 10-16 standard deviations above the mean value for blank chambers) on at least 1 of 2 devices (Fig. 2g and Supplementary Fig. 5). After introducing Alexa 647-labeled, C-terminally SpyTag003-tagged^21^ Omicron RBD target (Fig. 2f), additional imaging revealed concentration-dependent increases in Alexa 647/meGFP intensity ratios (which report on expression-normalized target binding), consistent with specific binding (Fig. 2h). Fitting fractional occupancies as a function of introduced Omicron RBD concentration to a quadratic binding equation yielded *K*_d_ values that were highly replicable across two devices (Pearson *R*^2^ = 0.91, RMSLE = 0.64, Fig. 2i, Supplementary Fig.6). *K*_d_ values spanned >2 orders of magnitude (from 64.5 nM to 10.3 µM), with 14 designs exhibiting binding above the noise floor (as determined by a one-sided t-test with a Holm-Bonferroni correction comparing AF647 intensities of empty chambers to those of monobody-containing chambers on a per-design basis) (Fig. 2j and Supplementary Figs. 7, 8). Additional experiments using an orthogonal labeling approach (commercially available His-Avi-tagged Omicron RBD labeled with a fluorophore-conjugated avidin molecule, similar to the YSD experiments) also returned reproducible, high-quality binding curves (Supplementary Fig. 9). However, while the relative categorization of strong and weak binders was consistent across experiments, the dynamic range of measured *K*_d_s for the His-Avi-tagged Omicron RBD was compressed (Supplementary Fig. 10), making it difficult to achieve a precise one-to-one rank ordering between measurements. These compressed measurements likely result from a combination of avidity effects from using a tetrameric probe and sequence-specific avidity effects that amplify background binding. (Supplementary Note 1 and Supplementary Figs. 11,12).

### Correspondence between *in cellulo* and in vitro affinities

**Fig. 3:**
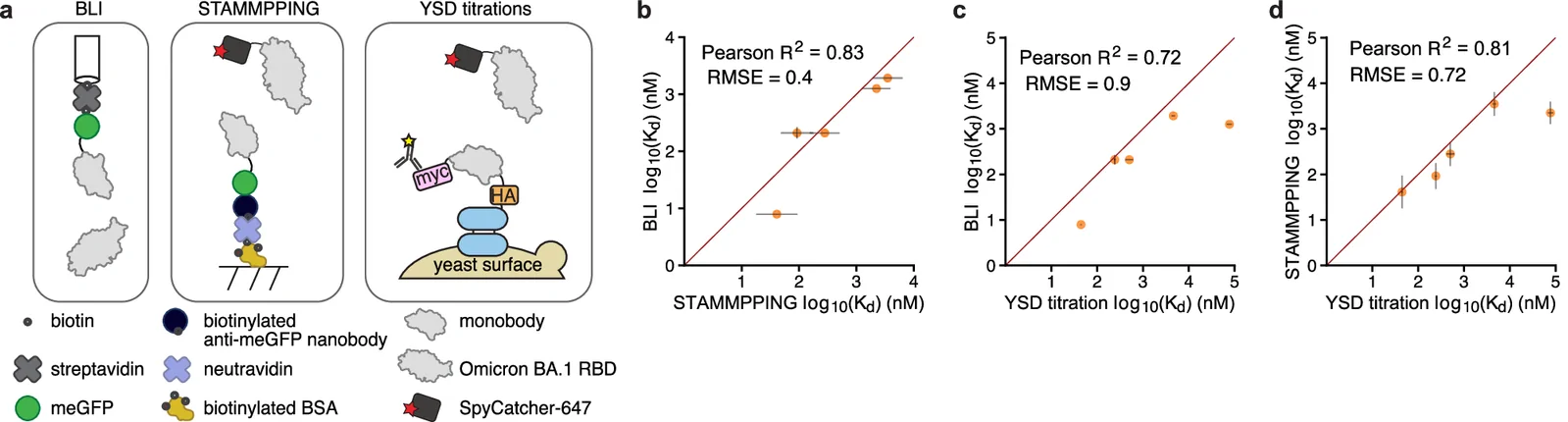
Correspondence between in vitro and *in cellulo* binding measurements. **a** Cartoon representation of surface chemistry for biolayer interferometry (BLI) measurements (left), STAMMPPING measurements (center), and yeast titration measurements (right). **b** Pairwise comparison between BLI *K*_d_ measurements (y-axis) and STAMMPPING *K*_d_ measurements (x-axis). **c** Pairwise comparison between BLI *K*_d_ measurements (y-axis) and yeast titration *K*_d_ measurements (x-axis). **d** Pairwise comparison between STAMMPPING *K*_d_ measurements (y-axis) and yeast titration *K*_d_ measurements (x-axis). Pearson *R*^2^ values quantify linear correlation between measurements, and root-mean square error (RMSE) quantifies deviation from the 1:1 (red) line. Data are presented as median values ± SD. Source data are provided as a Source Data file.

To establish the reliability of the measurements made with STAMMPPING, we selected 5 clones with varying affinities and also measured *K*_d_ values via biolayer interferometry (BLI) and yeast surface titrations^22^ (Fig. 3a). STAMMPPING-derived *K*_d_ values correlated strongly with those obtained via label-free BLI (Fig. 3b, Supplementary Fig. 13, r2 = 0.83). Both measurements agreed with those derived from yeast-based titration assays (Fig. 3c, d and Supplementary Fig. 14, *r*^2^= 0.72 and *r*^2^ = 0.8, respectively), consistent with prior reports^23^. The largest discrepancy between measurements was for design s6450, which bound more weakly by yeast-based titration than by BLI and STAMMPPING. One possible explanation for the variation in measurements is reduced epitope accessibility on the yeast surface that could be limiting the labeling efficiency of this particular design and thereby underestimating the *K*_d_.

Overall, variants with high YSD log enrichment scores generally bound with high affinity (i.e. low *K*_d_) when assessed by STAMMPPING (Supplementary Fig. 15); nevertheless, a substantial population (17/31 candidates with expression above the expression threshold) exhibited high enrichment via YSD and no detectable binding by STAMMPPING. Observed strong binding within YSD experiments could reflect extrinsic properties inherent to the YSD environment (*e.g*. multivalent avidity effects, high local target concentrations, nonspecific binding to the yeast cell wall, and unexpected glycosylation patterns), motivating the use of ADAPT-M, which decouples binding from variant expression and presentation on the cell surface.

### STAMMPPING in vitro dissociation measurements

Although equilibrium binding affinities provide a useful summary of interaction strengths, they do not provide information about underlying association (*k*_on_) and dissociation (*k*_off_) kinetics that describe how rapidly complexes form and come apart. Differences in kinetic parameters can lead to distinct functional outcomes, such as enhanced efficacies from prolonged residence times^24,25^ or limited adverse effects enabled by rapid reversibility^26,27^. To begin to disentangle these processes, we monitored RBD-monobody complex dissociation over time.

**Fig. 4:**
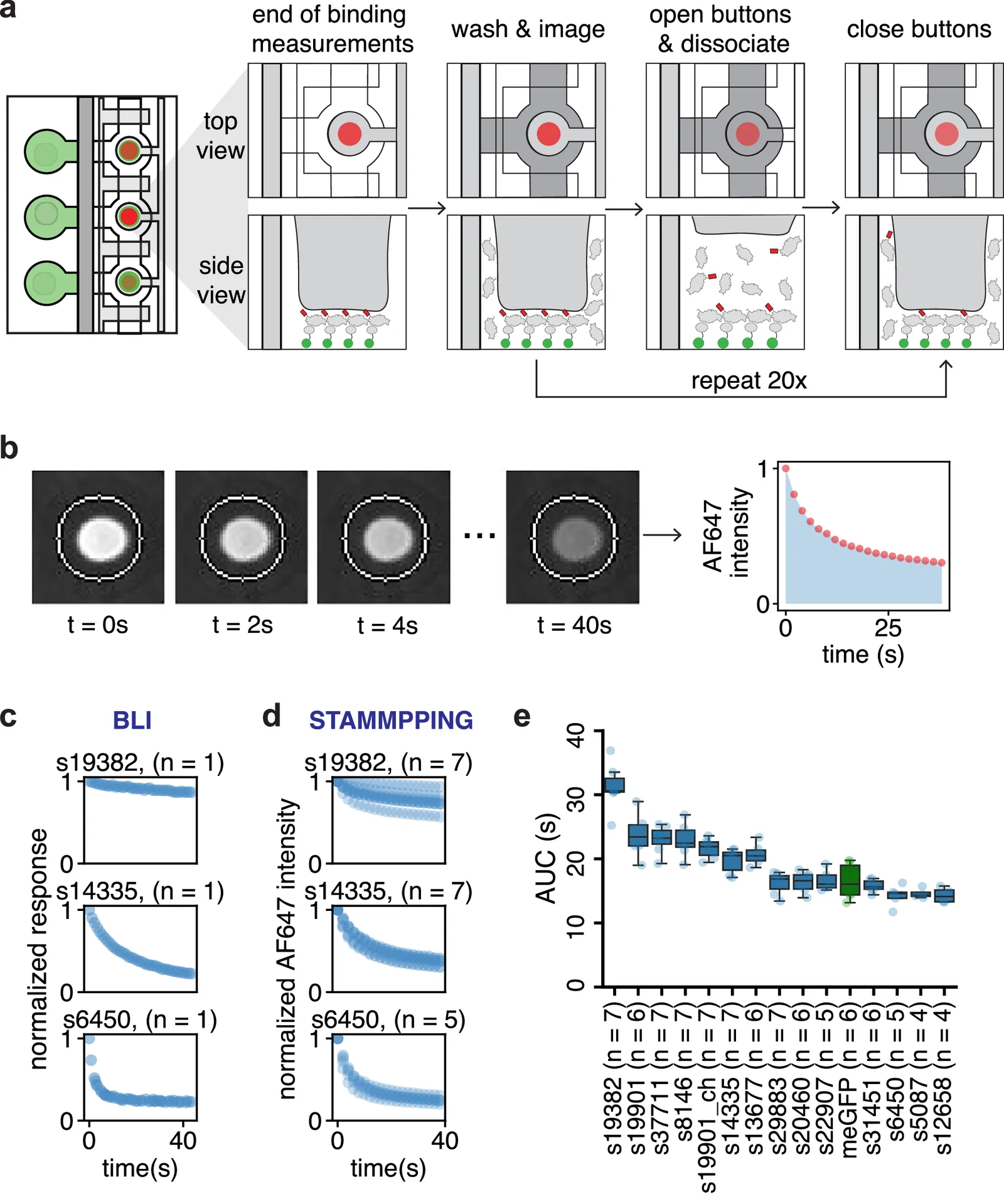
Dissociation measurements of designed monobodies via STAMMPPING. **a** Schematic of dissociation measurement protocol^28–30^ on STAMMPPING^13^. At the end of the binding titration series, button valves are closed, and chambers are washed with an unlabeled competitor before imaging. Button valves are then opened for 2 s to allow dissociation of labeled target protein, after which they are closed again to trap residual binding interactions. This cycle is repeated to monitor dissociation over time. In the side view schematic, meGFP (green) covalently linked to a monobody (gray) is shown bound to AF647-labeled RBD (red/gray). Unlabeled competitor RBD is depicted in gray. **b** Example time-course images of one reaction chamber, demonstrating dissociation of the Omicron BA.1 receptor binding domain (RBD) over time (left). AF647 intensities are plotted against time to extract qualitative kinetic dissociation measurements (right). **c** Dissociation traces measured with biolayer interferometry (BLI). **d** Dissociation traces measured with STAMMPPING. **e** Swarm plot summarizing measured area under the curve (AUCs) for designs with binding to RBD above the limit of detection, variable number of replicates per design. Box plots show the median, interquartile range (25th–75th percentiles), and whiskers extending to 1.5× IQR. Source data are provided as a Source Data file.

Microfluidic devices similar to those used for STAMMPPING have previously been used to quantify rates of dissociation (*k*_off_s) for DNA oligonucleotides bound to surface-immobilized transcription factor proteins^28–30^. Here, we adapted this method to quantify dissociation rates for target RBD bound by surface-immobilized designed monobody binders by equilibrating binding at high target concentration, opening button valves to allow dissociation, closing them to “trap” remaining bound material, and imaging (Fig. 4a). These dissociation measurements typically take place in the presence of a >10-fold molar excess of unlabeled target protein to prevent rebinding^31^.

Fluorescence images of chambers revealed loss of Alexa647 signals over time, consistent with expected RBD dissociation (Fig. 4b). However, measured STAMMPPING intensities decreased in 2 phases: a fast initial loss of RBD followed by a slower phase that did not decrease back to background levels; measurements of dissociation via BLI revealed a similar plateau (Fig. 4c,d). While the presence of additional molecular states complicates attempts to fit observed intensity traces and extract dissociation rates via either STAMMPPING or BLI, the traces clearly establish that RBD dissociates substantially faster from some complexes than others. To quantify these differences, we calculated the area under the curve (AUC) of fraction bound over time for each measured dissociation event. Median AUCs per design ranged from 14—30 s (Fig. 4e and Supplementary Fig. 16), with binders falling into approximately 3 regimes—slow, moderate, and fast dissociators.

### Characterization of lead designs via mutational and structural mapping

Results thus far establish that many designed monobodies sequence-specifically bind the RBD with high affinities, but do not reveal whether interactions take place at the designed interface. Here, we selected 2 designs for further characterization: s19382, the highest affinity binder with the slowest dissociation rate, and s14335, the second strongest binder, with dissociation kinetics similar to those of the other designs in this study. Notably, while s19382 ranked among the top designs (4th) by yeast log enrichment score, s14335 was only moderately enriched, ranking 23rd overall (Supplementary Fig. 15).

**Fig. 5:**
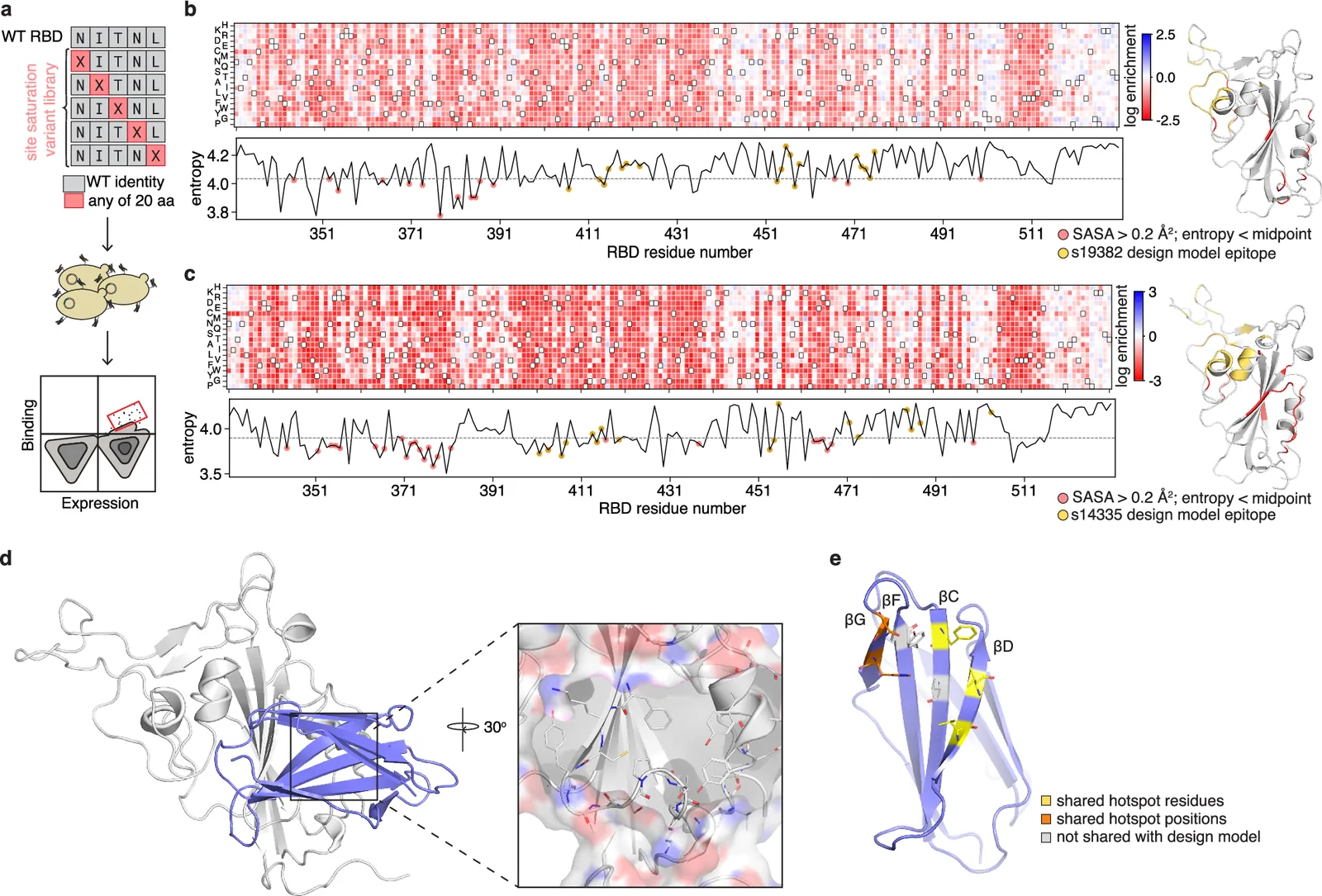
Epitope mapping of s19382 and s14335 binding via deep mutational scanning and cryo-EM. **a** Cartoon schematic of the epitope mapping strategy using yeast surface display. A site-saturation mutagenesis library of the Omicron BA.1 receptor binding domain (RBD), containing all possible single amino acid substitutions at each position (top), was expressed on the surface of yeast (center). Cells were stained with either monobody s19382 or s14335, and populations retaining binding were sorted to identify residues contributing to binding specificity (bottom). Epitope identification for (**b**) s19382 and (**c**) s14335. Heat maps (top) derived from deep sequencing of sorted libraries, visualizing the impact of mutations on binding. Wildtype sequence outlined in black. Sequence entropy plots (bottom), with dashed black lines indicating cutoffs to define sensitive residues. Red scatter points mark residues with entropies below this threshold and solvent-accessible surface area >0.2 Å^2^. Gold scatter points mark residues at the interface in the design model. These positions are mapped onto a structure model of RBD (right). **d** Cryo-EM structure (left) of Omicron RBD (silver) in complex with monobody s19382 (purple). A close-up view highlights residues involved in the binding interface. (right). Dashed yellow lines denote polar contacts. **e** Cryo-EM structure of s19382 with Rosetta-calculated hotspot residues shown as lines. Yellow indicates residues predicted as hotspots in both the design model and the experimental structure. Orange indicates paratope positions predicted as hotspots in both models, although specific contact residues differ due to beta-strand register shift. Gray indicates hotspot residues identified only in the experimental structure. Source data are provided as a Source Data file.

To characterize the binding interfaces, we used YSD to express a previously published site-saturation mutagenesis (SSM) library of Omicron BA.1 RBD^32,33^ and sorted the library separately against each binder (Fig. 5a and Supplementary Figs. 17, 18), reasoning that RBD mutations at the binding interface should drive loss of binding. Following sorting, we calculated positional Shannon entropies from NGS, thresholded on Shannon entropy to identify regions with mutational tolerance and on solvent-accessible surface area to select surface-exposed residues, and then mapped the resulting positions onto the structure of Omicron RBD (Fig. 5b,c)^34–36^. Both s19382 and s14335 appeared to bind a similar region on RBD, with the s19382 binding interface appearing less mutationally sensitive compared to s14335. Further, mutational scanning results suggest that both monobodies engage RBD in modes distinct from their respective design models (Fig. 5b, left; Supplementary Fig. 19 and Supplementary Tables 5,6). Given its higher affinity and slower dissociation, we focused first on s19382 to gain structural insight into this interaction.

To directly visualize this interaction, we determined the cryo-EM structure of s19382 in complex with the Omicron RBD Spike trimer (Supplementary Fig. 20a–e and Supplementary Table 4). Surprisingly, ab initio models showed that this monobody oligomerizes to facilitate Spike dimerization at its RBDs (Supplementary Fig. 20a). To molecularly characterize the s19382-RBD interaction, we performed a local refinement at the dimer interface to obtain a 3.16 Å resolution reconstruction that contained two RBDs from each Spike trimer and a total of eight s19382 monobodies (Fig. 5d and Supplementary Fig. 20). Here, strong binding is facilitated by shape complementarity between the concave, β-strand–driven binding surface of s19382 and a convex protrusion on the RBD. This interface shields a cluster of otherwise solvent-accessible aromatic residues on the RBD as well as a couple of solvent-exposed hydrophobic residues on s19382. Stabilization of the binding interface is achieved by strand-like pairing between βD of s19382 and the RBD at one end of the binding interface, and by hydrogen bonding between loop FG and the RBD at the opposite end, effectively latching s19382 onto the RBD. Together, hydrophobic residue burial and strand-like pairing provide a structural rationale for the compact binding footprint of s19382-RBD observed from deep mutational scanning experiments (Supplementary Table 5).

This overall recognition face in the solved complex shares similarity to that of the design model (Fig. 5e), in which s19382 binds to the RBD primarily through its FG loop and beta strands C and D. In the cryo-EM structure of s19382, packing between βA and βG is stabilized by a bulged conformation of βG and hydrogen bonding between the side chain of His80 and backbone atoms of Lys2 (Supplementary Fig. 21). In contrast, both the design model and AlphaFold3^37^ predictions position His-80 as solvent exposed. We hypothesize that this may result from the large number of monobody structures in the PDB featuring a solvent-facing Ser at this position, which could explain why neither our design model nor AlphaFold3, both trained on existing structural datasets, captured the observed packing.

The internal packing of His80 is critical to the binding mode of s19382. Packing of His80 towards the core results in a register shift in strand G as compared to the design model, altering the positions at the designed interface, particularly affecting residues on βG (Supplementary Fig. 21). Despite the discrepancy in the binding epitope and structure of loop FG, 13 of 16 designed paratope residues on s19382 also interface with Omicron RBD in the experimental structure, with a 1.02 Å cα root-mean-square deviation (RMSD) between the monobody design model and the solved structure of s19382 (Supplementary Fig. 22).

**Fig. 6:**
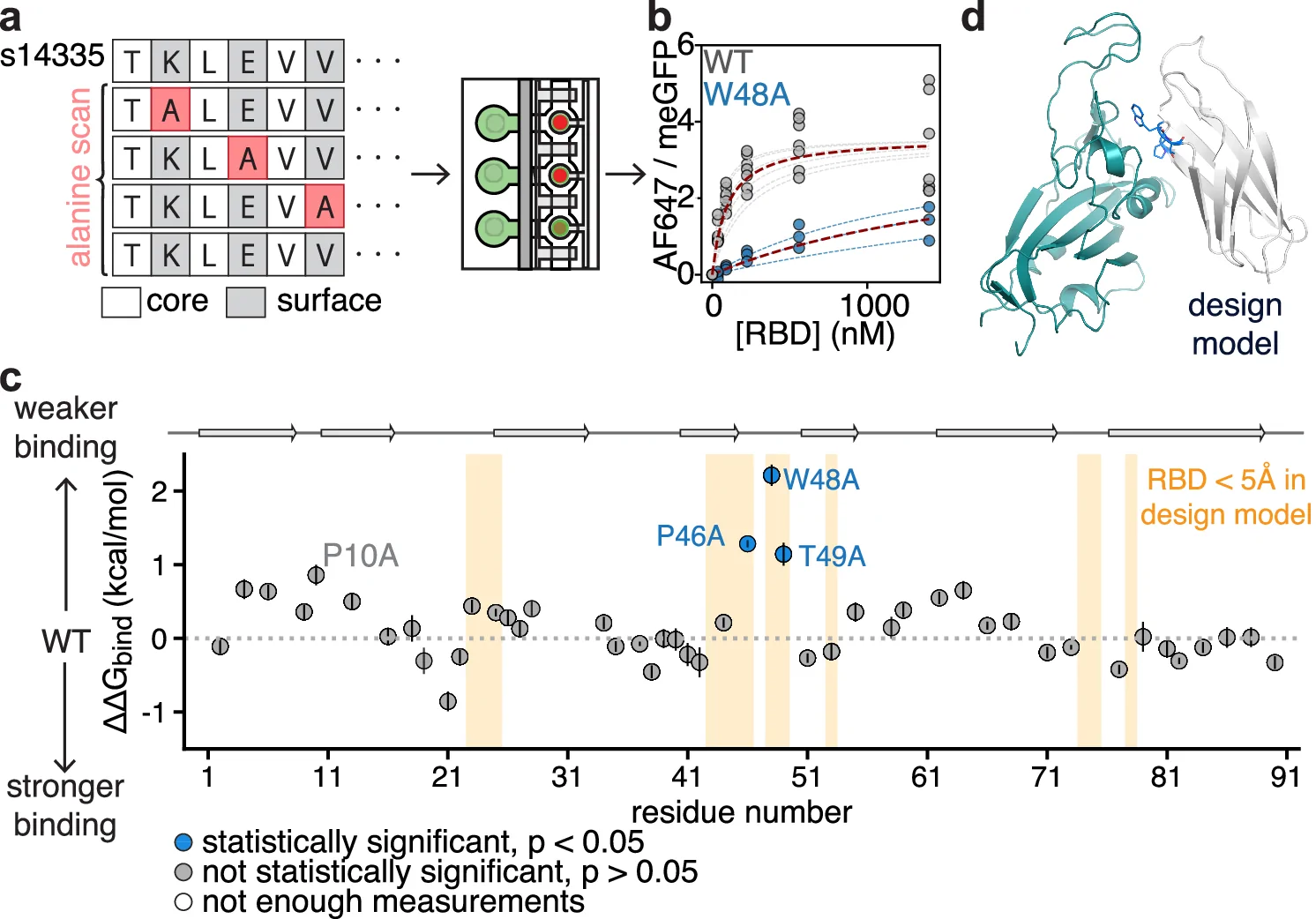
s14335 paratope mapping via STAMMPPING. **a** Cartoon schematic of the paratope mapping strategy on STAMMPPING^13^. An alanine scan library of monobody s14335, where each residue is mutated to alanine one at a time (left), was printed for analysis via STAMMPPING (right). **b** Langmuir isotherm fits for the wildtype sequence and variant W48A. **c** Scatter plot of median per-residue contributions to the binding free energy (y-axis) plotted against residue position on s14335 (x-axis) across two devices. Yellow spanning regions highlight residue positions that have a root-mean square deviation (RMSD) < 5 Å to residues on the Omicron BA.1 receptor binding domain (RBD) in the design model. Blue scatter points denote hot spot residues, defined as a statistically significant contribution to binding of >1 kcal/mol as determined by a one-sided t-test with a Holm-Bonferroni correction (P46A, *p* = 0.0089; W48A, *p* = 0.00000002; T49A, *p* = 0.00093). Gray scatter points denote residues where mutation to alanine results in a <1 kcal/mol contribution to binding. Vertical bars on scatter points indicate standard error. **d** Design model of s14335 (gray) binding to RBD (teal). Hotspot residues identified using STAMMPPING are shown as blue lines. Source data are provided as a Source Data file.

As we were unable to determine a structure for s14335, we next sought to map its paratope using STAMMPPING. Using this approach, we measured the effects of alanine substitutions at each surface-exposed position within the designed monobody and quantified the loss of affinity (ΔΔ*G*) to RBD (Fig. 6a, b and Supplementary Fig. 23), reasoning again that mutations at the binding interface should drive loss of binding. Alanine scanning revealed 3 hot spot residues on s14335, Pro46, Trp48 and Thr49, with mutation of Trp48 to alanine completely ablating binding to below the limit of detection (Fig. 6c). All 3 hotspot residues lie on loop DE, part of the designed paratope region of s14335 (Fig. 6d), suggesting that s14335 retains at least elements of the designed paratope interface, despite binding to an alternative epitope on Omicron RBD.

## Discussion

In this study, we introduced ADAPT-M, a workflow to adapt enriched binder sequences from YSD for in vitro affinity determination on a microfluidic platform in under 1 week’s time. We validated the ADAPT-M workflow on a library of designed monobody binders targeting the RBD of the SARS-CoV-2 Omicron BA.1 variant. Of the 36 highly enriched candidates, 33 were successfully recovered, and binding was evaluated for a total of 51 unique design sequences with ADAPT-M. From these in vitro results, we selected 2 lead candidates for deeper characterization. Using YSD, cryo-EM, and STAMMPPING, we mapped the binding interfaces for both. Structural analyses confirmed that both s19382 and s14335 bind to the C4 epitope^38^ of the RBD, engaging a similar region to that recognized by the heavy chain variable domain of the Class IV binder, CR3022^39–41^. Due to this shared epitope, it is likely that these monobodies bind only in the ‘up’ RBD conformation, similar to CR3022^38^.

Through mutational and structural studies, we found that both monobodies retain features consistent with the designed paratope interfaces, despite binding to an off-target epitope. This may suggest that the design metrics used here (see Methods), favoring large, hydrophobic interfaces, may be insufficient in evaluating alternative epitope binding modes, especially when flexible loops are involved. Moreover, the EM-resolved binding modes for these monobodies are not predictable by AlphaFold3, despite the high specificity and affinity observed in our experiments. The shared epitope for the 2 monobodies structurally characterized here may point to a patch on RBD that is particularly “sticky” for our designed library. Indeed, library sequences that were highly enriched had a lower Rosetta ΔΔ*G*_hydrophobic_ (i.e., having a more hydrophobic interface) compared to the other sequences in the input library (Supplementary Fig. 24). For the quantitative affinity measurements, no particular metrics, including AlphaFold3 iptm scores, appeared to have any correlation with in vitro binding affinity (Supplementary Fig. 25), highlighting the limitations of current design metrics and the complexity of protein-protein recognition. These challenges also suggest why methods like ADAPT-M are critical, as they should provide rich data on many binders for further ML model improvements and can nominate particular designs for additional studies to resolve binding modes.

While we focused here on a small computationally designed library, ADAPT-M is broadly applicable to diverse protein scaffolds and library types. The workflow is not constrained by initial library size, as it leverages enrichment through YSD screening to reduce the pool to a manageable set of candidate sequences. Despite YSD’s scalability, false positives can persist post-selection due to nonspecific binding at high analyte concentrations, multivalent avidity effects arising from surface display, and/or unexpected glycosylation events. In principle, these false positives can be reduced through adjustments to the enrichment workflow (e.g. lower analyte concentrations, negative selection with off-target analytes). ADAPT-M offers an orthogonal quantitative workflow that filters out false positives post-enrichment, enabling rapid discrimination between true high-affinity binders and YSD artifacts.

As with other surface-based affinity assays, factors such as surface heterogeneity, density-dependent effects, avidity, and potential mass-transport limitations could influence ADAPT-M and STAMMPPING affinity and kinetic measurements. In particular, high surface densities may promote re-binding or multivalent interactions that deviate from 1:1 binding models. To mitigate these effects, our platform allows deliberate tuning of surface density, our analyses are performed by comparing variants under similar surface conditions, and we included a “dark” competitor in dissociation assays to minimize rebinding.

ADAPT-M and STAMMPPING are also limited by factors intrinsic to all fluorescence-based assays. As with all assays that label ligands, the presence of the label may impact measured affinities and dissociation kinetics. In addition, the minimum quantifiable surface density of the immobilized protein (here, low nanomolar) defines the limit for strong interactions that can be measured without entering a titration regime, while the maximum concentration of the protein that is flowed on defines the limit for quantifying weak interactions. In the results presented here, STAMMPPING measurements for monovalent-labeled RBD correlated well with measurements made via label-free BLI (*R*^2^ = 0.83), suggesting that monovalent tags do not substantially impact the affinities.

Finally, while ADAPT-M and STAMMPPING can, in principle, be used to quantify dissociation rate constants (k_off_), the presence of multiple kinetic microstates in the RBD-monobody interactions studied here produced multi-phase dissociation behaviors (also present in the BLI measurements) that could not be reliably fit with a single exponential model. Since similar microfluidic devices have previously been used to quantify dissociation rate constants for DNA oligonucleotides bound to surface-immobilized transcription factor proteins, STAMMPPING should also be able to quantify these interactions for single-state dissociation of protein-protein interactions^28–30^.

As advances in artificial intelligence continue to transform structure-based design, ADAPT-M offers a powerful bridge to accelerate experimental validation and rapidly inform model development. The workflow is also fully compatible with mutational scanning libraries, enabling detailed studies of binding energetics and sequence-function landscapes. More broadly, we envision that this workflow can be integrated into many other existing protein library characterization pipelines to extract additional biophysical insights. For example, the workflow is readily compatible with microfluidic platforms such as SPARKfold^42^, which enables high-throughput measurements of protein unfolding rates via native proteolysis, providing information on protein kinetic stability.

All together, ADAPT-M provides a versatile workflow for bridging YSD screening with in vitro biophysical characterization, accelerating the development and functional understanding of protein binders across diverse library types.

## Methods

### Computational design of SARS-CoV-2 Omicron/BA.1 RBD binders

An early version of the Sculptor^43^ algorithm was used to generate 33,095 trajectories for target binding to a defined epitope on Omicron RBD (PDB: 7tn0^44^), using a monobody generator^45^. During sequence design, a monobody framework sequence was provided, but mutations were allowed during interface design. Sequence design was completed in two stages, comprising backbone generation followed by sequence optimization to stabilize the fold, using RosettaScripts^46^ and PyRosetta^47^. 11,000 top-ranking designs were selected by joint ranking of Rosetta metrics: ddg, interface_buried_sasa, and interface_sc. Disulfides were designed onto the 1000 top-ranking sequences, and these variants were denoted by a ‘ds’ suffix.

Framework sequence:

VSSVPTKLEVVAATPTSLLISWDAXXXXVXXYRITYGETGXXXXXQXFXVPXXXXTATISGLSPGVDYTITVYAXXXXXXXSPISINYRT

### Library synthesis and yeast transformation

A library of 12,000 monobody designs against the SARS-CoV-2 Omicron/BA.1 RBD was codon optimized for *Saccharomyces cerevisiae* and synthesized as a single-stranded 300-nucleotide DNA oligo pool by Twist Biosciences. The oligo pool was amplified with Phusion DNA polymerase for 12–14 cycles. Long oligos were used during amplification to include upstream and downstream homology to the pCTCON2 yeast surface display vector. PCR reactions were pooled and column purified using the Qiagen PCR Purification kit and eluted into 30 μL molecular grade water. 4 μg of the amplified and extended library were combined with 1 μg of gel purified, linearized (NheI, BamHI) pCTCON2 backbone and concentrated by a CentriVap DNA vacuum concentrator to a final volume of less than 10 μL. Concentrated DNA was transformed into *Saccharomyces cerevisiae* yeast EBY100 cells following an established protocol^2^. The transformed library was grown in SDCAA media for 24 h in a shaking incubator at 30 °C. Yeast cells were then passaged into fresh SDCAA media and grown overnight. The following day, a subset of the library was stored in a 20% glycerol solution at −80 °C, and the remaining subset was induced for expression.

### Yeast surface display and library screening

To induce expression, passaged yeast cells were centrifuged at 2000 × g for 5 min and resuspended in SGCAA media at a final seeding density of 1 × 10^7^ cells/mL. Expression was induced for 18–20 h at 30 °C in a shaking incubator. To prepare yeast cells for sorting, 1–2 × 10^7^ induced yeast cells were washed three times with PBSA (PBS containing 0.5% BSA). Yeast cells were labeled with anti-c-myc fluorescein isothiocyanate (FITC, Miltenyi Biotec) (1:100) and His-Avi-tagged SARS-CoV-2 Omicron/BA.1 RBD (Acro Biosystems, SPD-C82E4) (1 μM final concentration) for 60 minutes at room temperature on a rotating platform. Yeast cells were then washed once, resuspended in PBSA, and labeled with Streptavidin, R-Phycoerythrin (SAPE, Invitrogen) (1:100) for 10–15 min at room temperature on a rotating platform. Following secondary labeling, yeast cells were washed once in PBSA and kept on ice as pellets. Just before sorting, yeast cells were resuspended in PBSA and passed through a 35μm mesh filter. Yeast cells were sorted using a Sony SH800S FACS instrument with 70 μm sorting chip. Singlets were isolated (Supplementary Fig. 4) and then gated for FITC+SAPE+ signal above SAPE+ only control. Selected yeast cells were sorted into SDCAA media supplemented with 50 U/mL penicillin-streptomycin (Gibco), grown shaking overnight at 30 °C, and induced for consecutive rounds of selection.

### Yeast miniprep

Yeast cells were miniprepped using a Zymoprep Yeast Plasmid Miniprep II kit (Zymo Research) with the following modifications: 1 × 10^8^ cells were used for plasmid isolation; following resuspension in 200 μL Solution 1, cells underwent one to two freeze/thaw cycles prior to proceeding to step three; the amount of Zymolase used was double that recommended; and cells were incubated at 37 °C for a minimum of 2 h.

### NGS sample preparation and analysis

Samples were amplified using primers designed according to standard primer design criteria to append partial illumina adapter sequences. Primers were sequenced by Integrated DNA Technologies (IDT), and sequences are provided below:

FWD:5’-ACACTCTTTCCCTACACGACGCTCTTCCGATCT

REV: 5’-GACTGGAGTTCAGACGTGTGCTCTTCCGATCT

To minimize PCR bias, a cycle number scan was set up using 8 μL reaction volumes to determine the optimal number of cycles for amplification. 8 μL reactions were set up as follows: 4 μL 2x Q5 Mastermix (New England Biolabs), 0.4 μL of forward + rev primer mix, 10 μM each, 6.4 ng template, water to 8 μL total volume. Thermocycling conditions: 98 °C, 30 s → (98 °C, 10 s → 62 °C, 10 s → 72 °C, 15 s) × N cycles → 72 °C, 2 min → 4 °C, Hold. The library was then amplified at this optimal cycle number in a scaled-up 400 μL reaction volume. The entire PCR reaction was loaded onto a 1.2% agarose gel and gel purified using a Qiagen gel purification kit. Concentrations were determined by Qubit (Invitrogen) and submitted for paired-end 250 at Azenta (South Plainfield, NJ) or paired-end 300 at Neochromosome (Long Island City, NY) for NGS sequencing.

BBMerge^48^ (V39.01) was used to merge demultiplexed reads, Cutadapt^49^ (V2.6) was used to trim adapters, and Bowtie2^50^ was used to align and map trimmed reads to the library reference FASTA. Custom Python scripts were used to translate sequences and calculate log enrichment scores^20^ from naive and enriched library counts as well as for downstream analyses.

### PCR amplification of enriched monobody library

Primers were designed according to standard primer design criteria to include flanking BsmBI-v2 recognition sites and were synthesized by Integrated DNA Technologies (IDT). The sequence format used is as follows:

forward: 5’-gatagat**CGTCTC**gctcc-[primer binding sequence]

reverse: 5’-atgctaat**CGTCTC**gatcc-[primer binding sequence]

The bolded regions represent the BsmBI-v2 recognition site. The specific full-length primers used for amplification here were:

sars_esp3i_forward: 5’-gatagatCGTCTCgctccACCAAACTAGAAGTAGTAGCC

sars_esp3i_reverse: 5’-atgctaatCGTCTCgatccCAAGTCCTCTTCAGAAATAAGC

To minimize PCR bias, a cycle number scan was set up using 8 μL reaction volumes. The full-scale 200 μL reaction was set up as follows: 100 μL 2x Q5 Mastermix (New England Biolabs), 10 μL of forward + rev primer mix (10 μM each), 56 ng yeast miniprep template, water to 200 μL total volume. Thermocycling conditions: 98 °C, 30 s → (98 °C, 15 s → 61 °C, 30 s → 72 °C, 20 s) × 19 cycles → 72 °C, 5 min → 12 °C, Hold. The entire PCR reaction was loaded onto a 1.2% agarose gel and gel purified using a Qiagen gel purification kit.

### Golden Gate assembly and transformation

pNVD006, an IVTT expression plasmid with C-terminal meGFP-tag (Addgene ID: 229642, https://www.addgene.org/229642/), was generated by digestion of the PURExpress DHFR control plasmid (New England Biolabs, N0424AVIAL) followed by ligation of a custom gene block and sequence verification by Sanger Sequencing.

For Golden Gate assembly, 100 ng of pNVD006, was combined with 23.4 ng of amplified monobody library (2x molar excess), 1 μL BsmBI-v2, 1 μL T4 DNA Ligase, 1 μL T4 DNA Ligase buffer, and water in a final 10 μL volume. A control reaction was set up as above, replacing the amplified monobody library with water. The library assembly and control reactions were thermocycled as either (42 °C, 5 min → (16 °C, 5 min → 42 °C, 5 min) × 65 → 60 °C, 15 min → 4 °C Hold) or as (42 °C, 1 hr → 60 °C, 5 min). Following completion of the Golden Gate reaction, tubes were kept at −20 °C until transformation. 1 μL of Golden Gate assembly reaction mixture was used to transform 50 μL of high efficiency chemically competent *E. coli* cells (NEB, C2987H) according to the manufacturer's protocol. Transformations were either plated or diluted into a 20 mL volume of LB, depending on the method for single clone isolation.

### Colony picking

Following transformation, cells were plated onto 15 mm LB agar plates at a density of 100–200 colonies per plate. 70 μL of either LB or 2xYT medium supplemented with 50 μg/mL carbenicillin was dispensed into wells of a 384 F-bottom plate (Greiner Bio-One) using a multichannel pipette. P200 tips were used to pick single bacterial colonies and briefly introduced into each well, using a new tip in between picking and touching each tip to a single well only briefly. Plates were sealed and incubated at 37 °C overnight without shaking for at least 16 h.

### Single-cell sorting of bacteria

Following transformation, cells were diluted into 20 mL LB media supplemented with kanamycin and grown overnight at 37 °C in a shaking incubator to retain library uniformity^51^. In the morning, 30 μL of saturated overnight culture was used to inoculate a 3 mL culture and grown at 37 °C in a shaking incubator for 2.5–3 h to an optical density of 0.6–1.0. About 1.5–2 h after inoculating cultures, a Sony SH800 FACS instrument was started up, calibrated using a 70 μm chip, and droplet sorting was aligned to an F-bottom 384-well plate. 70 μL of 2xYT medium supplemented with 50 μg/mL carbenicillin was dispensed into all wells of a 384 F-bottom plate (Greiner Bio-One) using a multichannel pipette. Importantly, we noticed lower cell viability when using LB media instead of 2xYT media.

After cultures reached the target OD, 5 × 10^7^ cells (assuming 1 × 10^8^ cells/mL at OD = 1) were washed once in PBSA (PBS supplemented with 0.5% BSA). Cells were resuspended in a 1 mL volume and labeled with 5 μL of 1 mg/mL Propidium Iodide (PI) and incubated for 5 minutes on a tube rotator, protected from light. Following incubation, cells were washed once in PBSA and resuspended to a final cell concentration of 0.25 × 10^6^ cells/mL before being passed through a 35 μm mesh filter. Cells were loaded onto the sorter, gated by size using FSC and SSC channels, and then gated for singlets (Supplementary Fig. 1). PI- cells were sorted in single-cell sorting mode at a sample pressure of 3 with an average event rate of 500–800 events per second. After sorting, plates were sealed and incubated at 37 °C without shaking for at least 24 h.

### Linear template amplification and Sanger sequencing

4.224 mL of PCR mastermix was prepared in a laminar flow hood by combining reagents into a 15 mL falcon tube as follows: 844.8 μL 5x Q5 reaction buffer, 84.48 μL 10 mM dNTPs, 21.2 μL 100 μM T7long_forward, 21.2 μL 100 μM T7long_reverse, 42.24 μL Q4 polymerase, 3.21 mL ice-cold nuclease-free water. The mastermix was kept on ice during plate preparation. The volume was calculated as 384 wells * 10uL volume/well *1.1. Primers were synthesized by Integrated DNA Technologies (IDT) and are shared below:

T7long_forward: 5’GCGAAATTAATACGACTCACTATAGG

T7long_reverse: 5’CTTTCAGCAAAAAACCCCTCAAG

A mosquito LV liquid handler (SPT Labtech) was used to aspirate and dispense 50 nL of overnight liquid cultures and dispense into individual wells of a 384-well PCR plate (BioRad). A mantis microfluidic liquid dispenser (Formulatrix) was used to dispense 10 μL PCR mastermix into each well. Plates were tightly sealed, lightly vortexed, and centrifuged before being placed into a thermocycler with a 384-well reaction module (BioRad). Thermocycling conditions: 98 °C, 3 min → (98 °C, 10 s → 63 °C, 20 s → 72 °C, 50 s) × 30 cycles → 72 °C, 5 min → 4 °C, Hold.

2-4uL of 384-well plate samples were submitted for Sanger sequencing to Elim Biopharmaceuticals (Hayward, CA). Sequencing chromatograms were manually inspected and used to map wells back to their corresponding design sequence in the library. Only STAMMPPING chambers corresponding to wells containing a single design sequence were selected for downstream analysis.

### Plasmid template preparation

For validation experiments with Spy647-labeled RBD, wells containing selected designs were PCR amplified using 50 µL reactions with primers appending BsmBI-v2 overhangs to the protein-coding sequence as described above, and reactions were PCR purified.

4 μL-scale Golden Gate assembly reactions were set up in a 96-well plate (0.4 μL BsmBI-v2, 0.4 μL 10x T4 DNA Ligase buffer, 0.4 μL T4 DNA Ligase, 0.8 μL 10 ng/μL insert, 34 ng nvd006 template, water to 4 μL). Reactions were thermocycled overnight as follows: 42 °C, 5 min → (16 °C, 5 min → 42 °C, 5 min) × 65 → 60 °C, 15 min → 4 °C Hold. 2 μL of each Golden Gate reaction were transferred into a fresh 96-well plate, and a 20 uL volume of DH5alpha cells (Zymo) was added to each well for transformation. Following transformation, a 245 mm agar plate was divided into an appropriate number of grids, and 4 µL of each transformation was plated onto individual grids. Plates were grown overnight at 30 °C. A single colony was cultured and miniprepped using a Qiagen miniprep kit. Plasmids were sequence validated.

### s14335 alanine scan library template preparation

A custom Python script was used to identify residues with a SASA > 0.2 Å^2^ in the design model and generate a FASTA sequence file with the mutated DNA sequence. The residue list was inspected via visualization in Pymol and updated as necessary. BsmBI-v2 recognition sites were appended to both ends of the coding sequence. The GCG alanine codon was selected for all alanine mutations. Sequences were ordered from IDT as e-blocks in a 96-well plate. Plasmids were prepared as in the section above. Flanking sites:

5’ flank: gatagatCGTCTCgctcc

3’ flank: ggatcGAGACGattagcat

### Preparation of epoxysilane-coated slides

Plain 25 × 75 mm glass slides (Corning) were functionalized with epoxysilane (based on ref. ^52^). First, slides were cleaned in an 80 °C heated bath of hydrogen peroxide and ammonium hydroxide for 30 min, rinsed, and blow-dried with nitrogen. Next, epoxysilane was deposited by submerging slides in toluene doped with 3-glycidyloxypropyl-trimethoxymethylsilane (3-GPS, Millipore-Sigma) for 20 min. Slides were then rinsed in toluene and dried by baking at 120 °C for 30 min.

### Printing DNA libraries onto slides

5–10 µL of DNA templates were transferred to a 384-well plate and resuspended in 2x “print buffer” (20 mg/mL UltraPure BSA, 24 mg/mL trehalose dihydrate, 100 mM NaCl). Templates were randomly assigned positions within a 32 × 56 array, with controls containing only a print buffer distributed throughout. Two 300-360 pL spots were arrayed on epoxysilane-coated glass slides using a SciFLEXARRAYER S3 (Scienion) outfitted with a PDC-70 Piezo Dispense Capillary nozzle with Type 1 coating. Between every sample, the nozzle was washed to ensure no cross-contamination. Fabricated devices were then aligned on top of this array by eye, placing one spot per DNA chamber using a stereoscope. Devices were baked on a hot plate (Torrey Pines) for 4 h at 80 °C.

### Mold and device fabrication

Two-layer microfluidic devices were cast from previously fabricated 15 µm tall flow and control molding masters^13^ using polydimethylsiloxane (PDMS) polymer (Momentive, RTV615). Control layers were generated by pouring a mixed 1:5 ratio of cross-linker to base (THINKY 3 min at 2000 rpm) onto the molds, degassing to remove all air in a vacuum chamber for 45 min, and baking for 60 min at 80 °C. Flow layers were generated by spin-coating a 1:20 ratio of mixed cross-linker to base onto the molds at 500 rpm for 10 seconds followed by 1800 rpm for 75 s. Layers were relaxed on a flat surface for 5–20 min at room temperature prior to baking at 80 °C for 40 min. Cut and punched device control layers were then aligned to flow layers using a stereoscope. Aligned devices were then baked for 50 min at 80 °C and excised from the molds using a scalpel.

### Microscopy instrumentation

Chip imaging was performed using a Nikon Ti-2 Microscope equipped with motorized XY-stage (Applied Scientific Instruments, MS-2000 XY stage), an LED light source (Lumencor SOLA SE Light Engine), a 4x Objective (CFI Plan Apo 4X NA 0.20, Nikon), and a CMOS camera (Andor Zyla 4.2). The Nikon Ti-2 core itself was equipped with an automated Z-axis, turret aperture, and filter turret, containing pre-arranged filter cubes for EGFP (Semrock, GFP-4050B) and Cy5 (Semrock, Cy5-5070A). All imaging was performed using 2×2 pixel binning with exposure times as follows: EGFP: 100 ms and 500 ms, Cy5: 50 ms, 100 ms, 500 ms, 1000 ms, and 3000 ms.

### STAMMPPING device operation and experimental pipeline

Microfluidic valves were operated via a custom-built open-source WAGO-controlled pneumatic manifold^53^. Automated control of pneumatic valves and microscopy was run through custom Python scripts (RunPack, AcqPack) as described previously^54^. Briefly, standard STAMMPPING experiments consist of the following steps: (1) pattern button-valve surfaces for bait immobilization, (2) express bait-meGFP constructs from DNA templates on chip, (3) iteratively introduce labeled prey protein, allow interactions to come to equilibrium, trap bound material using ‘button’ valves, wash away unbound material, and image in both the eGFP and Cy5 channels to measure binding, and (4) open the ‘button’ valves for a defined period of time to allow dissociation of prey protein before closing and imaging in both eGFP and Cy5 channels to measure dissociation kinetics. Steps 1 and 2 were carried out largely as described previously^13,54,55^, with the following modifications. For surface patterning, we used a biotinylated anti-GFP nanobody (Proteintech, gtb-250). We also optimized on-chip expression for monobody-meGFP constructs such that expression conditions varied from 25–37 °C for 2–6 h. Following expression and binding of monobody-meGFP to anti-GFP-patterned buttons, device chambers were washed in TrypLE and regions surrounding the protected buttons were re-patterned with biotinylated BSA or Neutravidin (Supplementary Note 1). For step 3, dye-labeled proteins made off-chip were prepared as serial dilutions in an Assay Buffer of 20 mM KH_2_PO_4_, 200 mM KCl, 5 mg/ml UltraPure BSA (Thermo Fisher), 0.05% v/v Tween-20 (Thermo Fisher) in UltraPure distilled water. On-chip iterative measurements of binding over multiple concentrations were carried out via scripts based on those used previously^13^. Although it is theoretically possible to re-use devices (i.e. test binding to a different target) after patterning, expression, and target dissociation, all STAMMPPING experiments here were performed from newly patterned and expressed devices.

### STAMMPPING image processing

Raw 7×7 image rasters of fluorescence images covering the full-chip area were flat-field corrected using manual flat-field images or an implementation of BaSiCpy^56^ and stitched into single full-chip images using ImageStitcher, an in-house publicly-available software package (https://github.com/FordyceLab/ImageStitcher). These stitched images were processed using ProcessingPack, an in-house publicly available software package (https://github.com/FordyceLab/ProcessingPack). Briefly, circle-finding algorithms identified fluorescent spots under chamber “button” valves and per-spot metrics were mapped to Scienion print array position. Downstream analysis primarily utilized background-subtracted summed intensities of GFP and Cy5 per-spot fluorescence.

### STAMMPPING quality control

Prior to fitting curves to determine binding affinities, we performed several quality control steps. First, we filtered out any chambers with GFP expression levels below the threshold set by considering blank chambers (mean + ten or more standard deviations). This leaves us with a narrow distribution of GFP intensities (and densities) for chambers analyzed for binding. While there is some variation (<2-fold) in chamber GFP fluorescence within a given experiment, this has a very small effect on fit *Kd* values (Supplementary Fig. 26). Second, we manually culled chambers that had visible dust or flow issues. Third, we computed a linear regression between measured Cy5 intensities of soluble prey in each chamber and the estimated input concentration and required *r*^2^ > 0.9 (ensuring that soluble prey was available for binding as expected, Supplementary Fig. 27).

### Determining fractional occupancies

Given the observation of decreasing meGFP intensity over the course of binding measurements and increasing meGFP intensity over the course of kinetics measurements (Supplementary Fig. 26), we suspected Förster Resonance Energy Transfer (FRET) between meGFP and AF647 fluorophores. FRET would artificially increase cy5/meGFP ratios at increasing Omicron RBD concentrations, to differing degrees for different monobody designs, owing to differing binding modes and strengths. To circumvent this, we use meGFP intensities measured at step 0, prior to any Omicron RBD introduction, to calculate response ratios at each step as AF647/meGFP_step0_, thereby making the assumption that meGFP is not lost due to dissociation or button “shearing” over the course of the binding experiment. For kinetics measurements, we make this same assumption and instead fit data to per-chamber summed AF647 intensities rather than AF647/meGFP ratios.

### Determination of STAMMPPING equilibrium binding constants

Considering the structural data show a 1:1 stoichiometry, we assume all binders/targets bind with the same stoichiometry. All *K*_*d*_s were estimated by global fitting of a quadratic solution to 1:1 binding equilibrium^57^, Eq. (1), to concentration-dependent data. Here, the concentration of immobilized monobody in each chamber was determined by dividing the summed, background-subtracted eGFP intensity for each chamber by an empirically-calculated slope (here, slope = 43,000 a.u./nM^13^), relating eGFP intensity to immobilized eGFP concentration, as has been demonstrated previously^54^.

1 $$	 {{{\rm{Ratio}}}}=\\ 	 {{{{\rm{R}}}}}_{\max }\cdot \frac{([{{{\rm{RBD}}}}]+[P]+{{{{\rm{K}}}}}_{d})-\sqrt{{([{{{\rm{RBD}}}}]+[{{{\rm{monobody}}}}]+{{{{\rm{K}}}}}_{d})}^{2}-4[{{{\rm{RBD}}}}][{{{\rm{monobody}}}}]}}{2\cdot [{{{\rm{monobody}}}}]}$$

In cases where ligand (putative binder) depletion is negligible, the data can be accurately fit using the standard Langmuir isotherm, Eq. (2), instead.

2 $${{{\rm{Ratio}}}}=\frac{{{{{\rm{R}}}}}_{\max }[{{{\rm{RBD}}}}]}{{{{{\rm{K}}}}}_{d}+[{{{\rm{RBD}}}}]}$$

We globally fit all binding data from the same experiment (i.e. single microfluidic device) to determine apparent *K*_d_s even for interactions that did not saturate at the highest concentrations tested. First, we estimated the saturation point for a given experiment by fitting the top binders (binders with a Cy5/GFP Ratio above a set threshold set to identify the most-saturated binders) to Eq. (1) with *K*_*d*_ and *R*_*max*_ as free parameters. The mean *R*_*max,top_binders*_ was then used as a global *R*_*max*_ to re-fit all remaining RBD-monobody interactions with *K*_*d*_ as a free parameter. From these fits, we calculated the median *K*_*d*_ value across replicates for each binder/target pair. In all cases, we remove outliers (>1.5 times the interquartile region (IQR) and report median *K*_d_s. All data fitting was done in custom Python scripts using open-source lmfit and scipy packages^58,59^.

### Qualitative measurement of dissociation kinetics

Qualitative dissociation kinetics were estimated by calculating the AUC from time-course plots of summed AF647 fluorescence intensities across all regions of interest, over the first 40 s of the dissociation measurement. AUC was computed by numerical integration using the composite trapezoidal rule implemented in NumPy^60^ (numpy.trapezoid).

### Determination of the limit of detection/noise floor

Due to the higher binding to “empty” chambers as compared to meGFP-only containing chambers, we used “empty” chambers to establish the limit of detection (Supplementary Fig. 7). To identify designs with binding above this limit of detection, we applied a one-sided *t-*test with a Holm-Bonferroni correction to compare the cy5 intensities of all empty chambers to the cy5 intensities of monobody-containing chambers, on a per-monobody basis. Monobodies with *p* < 0.05 were denoted as binding above the limit of detection, and those with *p* > 0.05 were categorized as having binding below this limit. Importantly, using empty chambers to establish the limit of detection provides a strict binding threshold, as meGFP-only containing chambers consistently exhibited weaker nonspecific binding than empty chambers. Because of this, binding affinities for binders near the noise floor are likely to be an overestimate.

### ΔΔG estimation

ΔΔG values were calculated by subtracting the wildtype sequence ΔG, Eq. (3), from each alanine variant ΔG, using a lower limit *K*_d_ when applicable. ΔΔGs were calculated per-device, and the median ΔΔG is reported.

3 $$\Delta G=-{{{\rm{RT}}}}\cdot {{\mathrm{ln}}}\left({{{{\rm{K}}}}}_{d}\right)$$

### SARS-CoV-2 Omicron/BA.1 protein expression and purification

Constructs encoding the SARS-CoV-2 Omicron BA.1 RBD (residues R328-L533 of GenBank QHD43416.1 sequence (G339D, S371L, S373P, S375F, K417N, N440K, G446S, S477N, T478K, E484A, Q493R, G496S, Q498R, N501Y, Y505H)) and either a C-terminal 3xFLAG tag or C-terminal SpyTag003 were transiently transfected into Expi293F cells (Gibco). After the addition of enhancers, cells were grown at 30 °C and 8% CO_2_. Supernatants were harvested 4–5 days after transfection and supplemented with 0.02% sodium azide (1000x) and 0.2 M PMSF (100x) before being spun down at 3500 g for 15 min and vacuum-filtered using a 0.22 µm mesh filter. Filtered supernatants were diluted 2-fold in ice-cold PBS and supplemented to a final 30 mM imidazole concentration before being loaded onto a pre-packed nickel affinity column (HisPur resin, Thermo Fisher Scientific) pre-equilibrated with PBS containing 50 mM imidazole. Resin was then washed with 10–20 column volumes of PBS containing 50 mM imidazole before being eluted over 12 0.5 mL fractions using PBS containing 500 mM imidazole. Eluted fractions were analyzed by SDS-PAGE. Fractions containing protein of interest were pooled and concentrated using Amicon Ultra−4 10 kDa MWCO centrifugal filters (Sigma Aldrich) to a 2 mL volume. Concentrated protein was purified by size-exclusion chromatography (Superdex 75 Increase 10/300 GL or Superdex 200 Increase 10/300 GL, GE Healthcare) into PBS. SEC was performed at 4 °C on an ÄKTA chromatography system at a flow rate of 0.5 mL/min, and 1 mL of concentrated protein was injected per run. Elution was monitored by UV absorbance at 280 nm, and fractions corresponding to the major monodisperse peak were collected and analyzed by SDS–PAGE. Concentrations were determined by absorbance at 280 nm measured with a NanoDrop8000 spectrophotometer (Thermo Fisher Scientific) using predicted extinction coefficients.

### SARS-CoV-2 Omicron/BA.1 fluorescent labeling (SpyTag-SpyCatcher-647)

2 mg of SpyCatcher003Cys (Bio-Rad) was resuspended in PBS in a final volume of 497.5 μL. To reduce disulfides, 2.5 μL of a freshly prepared 1 M DTT stock was added to reach a final concentration of 5 mM DTT, and the reaction was incubated at room temperature for 1 h. Meanwhile, a Superdex 75 10/300 GL column was equilibrated with degassed PBS. After incubation, the reduced SpyCatcher003Cys was immediately injected onto the column, and monomeric fractions were collected. SEC was performed at room temperature on an ÄKTA chromatography system at a flow rate of 0.75 mL/min, and 0.5 mL of concentrated protein was injected per run. To prevent the reformation of disulfides, conjugation with maleimide was initiated immediately.

Monomer fractions were pooled in a 15 mL Falcon tube with a mini stir bar. From Nanodrop A280 measurements, typical recovery following size exclusion was 1–1.2 mg of SpyCatcher003Cys. A 10 mM stock of AF647-C2-maleimide (ThermoFisher) was prepared by dissolving the full 1 mg vial in 80 μL of solvent, providing a 10–20 fold molar excess relative to the protein. All 80 μL of the dye stock were added dropwise to the reduced SpyCatcher003Cys while stirring. The reaction was protected from light and allowed to proceed under vacuum at room temperature for 2 h. Following conjugation, the protein was buffer exchanged via a Superdex 75 10/300 GL column to remove excess, unconjugated AF647 dye. Labeling efficiency was calculated according to the manufacturer’s protocol and typically exceeded 97%. The labeled protein was then concentrated using a 3 kDa MWCO Amicon filter (Millipore Sigma) to a working concentration of 80 μM.

After confirming successful labeling, SpyCatcher003-AF647 was reacted with Omicron BA.1-SpyTag003 at a 1.2:1 ratio, at a final 40 μM concentration, overnight at 4 °C. The next day, the conjugated protein was purified using a Superdex 200 10/300 GL Increase column equilibrated with ice-cold PBS. SEC was performed at 4 °C on an ÄKTA chromatography system at a flow rate of 0.5 mL/min and eluted over 0.5 mL fraction volumes. Fractions were analyzed by gel electrophoresis (Supplementary Fig. 28) to confirm conjugation, pooled, and aliquoted. Aliquots were stored at 4 °C for immediate use or -80 °C for long-term storage.

### Monobody protein purification and biotinylation

meGFP-tagged monobody sequences were amplified from the print plate using primers appending 5’ and 3’ overhangs to pET24-a(+) with and without an Avi-tag at the C-terminus of the fusion protein. Expression plasmids were assembled using gibson assembly in DH5alpha cells (Zymo Research), miniprepped, sequence verified, and transformed into BL21(DE3) *E.coli* cells (Zymo Research). A single colony was used to inoculate 5 mL 2xYT cultures supplemented with kanamycin and grown in a shaking incubator at 37 °C for at least 5 h. Cultures were then diluted in 500 mL 2xYT and grown until the optical density reached 0.6–1.0. Protein expression was induced by addition of isopropyl β-D-thiogalactopyranoside (IPTG) to a final 1 mM concentration and cultures were grown at 16 °C for 16–20 h. Following induction, cells were pelleted and resuspended in 30 mL of phosphate-buffered saline (PBS), pH 7.4 and frozen at −20 °C until purification.

Pellets were thawed at room temperature, and 4.2 mL of 4 M NaCl and 600 μL of 50 mM phenylmethylsulfonyl fluoride (PMSF) were added. Thawed pellets were sonicated on ice in a ~35 mL volume over 12 min using 30 s ON/59 s OFF pulses with a ¼” probe on a Q500 sonicator (Qsonica) operating at 20% power, ensuring the sample remained cold throughout sonication. Sonicated samples were then clarified by centrifugation at 20,000 × *g* for 30 min. The resulting supernatant was supplemented to a final 30 mM imidazole concentration and loaded onto a pre-packed nickel affinity column (HisPur resin, Thermo Fisher Scientific) pre-equilibrated with PBS containing 50 mM imidazole. Resin was then washed with 10–20 column volumes of PBS containing 50 mM imidazole before being eluted over six 1 mL fractions using PBS containing 500 mM imidazole. Eluted fractions were analyzed by SDS-PAGE. Fractions containing protein of interest were then pooled and concentrated using Amicon Ultra-4 10 kDa MWCO centrifugal filters (Sigma Aldrich) to a 1 mL volume. Concentrated protein was purified by size-exclusion chromatography (Superdex 75 Increase 10/300 GL or Superdex 200 Increase 10/300 GL, GE Healthcare) into either 50 mM Bicine, 100 mM NaCl, pH 8.3 buffer (Avi-tagged) or PBS (non-Avi-tagged). SEC was performed at room temperature on an ÄKTA chromatography system at a flow rate of 0.75 mL/min, and 1 mL of concentrated protein was injected per run. Elution was monitored by UV absorbance at 280 nm, and fractions corresponding to the major monodisperse peak were collected and analyzed by SDS–PAGE (Supplementary Fig. 28). Concentrations were determined by absorbance at 280 nm measured with a NanoDrop8000 spectrophotometer (Thermo Fisher Scientific) using predicted extinction coefficients.

Avi-tagged proteins were biotinylated using a BirA biotin-protein ligase standard reaction kit (Avidity), and the reaction was incubated overnight at 4 °C, followed by 1 h at room temperature. Proteins were then buffer exchanged into PBS, pH 7.4 buffer via size-exclusion chromatography to remove unreacted biotin. SEC was performed at room temperature on an ÄKTA chromatography system at a flow rate of 0.75 mL/min. The extent of biotinylation was assessed by streptavidin gel-shift analysis^61^.

### Biolayer interferometry

BLI experiments were performed on an Octet RED96 system (Forté Bio). Octet streptavidin (SA) biosensors (Sartorius) were rehydrated in a 1x kinetics buffer (Sartorius) for at least 30 min at room temperature. Biotinylated meGFP-tagged monobodies diluted in 1x kinetics buffer to a concentration of 0.8 µg/mL. FLAG-tagged Omicron RBD stocks were diluted first in PBS to 4–5x the target concentration and then diluted 4–5-fold into 1x Kinetics Buffer to ensure a consistent buffer composition for all concentrations in the series.

SA biosensors were loaded with biotinylated meGFP-tagged monobody for 120 s to 600 s to a density of 0.8 to 1.3 response units to ensure the loading density remained in the linear regime. Loading was stopped early if necessary. SA sensors were then dipped into a 1x kinetics buffer for 120 s to establish a baseline. Association was monitored by dipping sensors into wells containing 3xFlag-tagged Omicron BA.1 RBD for 120 s and dissociation was monitored by dipping wells into wells containing 1x kinetics buffer for at least 160 s. Titration experiments were performed at 25 °C while rotating at 1000 rpm. To measure *K*_d_, steady-state and global kinetic fits were performed using the manufacturer’s software (ForteBio Data Analysis 9.0) assuming a 2:1 heterogeneous ligand binding model.

### In cellulo K_d_ determination by yeast titrations

Monobody sequences were amplified from the print plate using primers appending 5’ and 3’ overhangs to pCTCON2. Reactions were PCR purified and assembled into a pre-digested (NheI/BamHI) pCTCON2 backbone via gibson assembly. Plasmids were miniprepped and sequence validated before being chemically transformed into EBY100 using a Frozen-EZ yeast transformation II kit (Zymo).

A single colony was selected per clone, passaged twice in SDCAA, and induced in SGCAA at a final seeding density of 1 × 10^7^ cells/mL. Expression was induced for 18–20 h at 30 °C in a shaking incubator. Using anti-c-myc FITC (1:100) and Omicron/BA.1 RBD Spy-AF647 as staining reagents, 3 × 10^6^ yeast were labeled per staining condition in a 30 µL volume according to a published protocol2, taking care to ensure the staining volume was modified when necessary to avoid the ligand titration regime. Following incubation, yeast cells were washed once in PBSA and kept on ice as pellets. Just before flowing, yeast cells were resuspended in PBSA and passed through a 35 μm mesh filter. Yeast cells were visualized using a Sony SH800S FACS instrument with 70 μm sorting chip.

### Omicron BA.1 RBD SSM library screening

The Omicron BA.1 library^33^ was ordered from Addgene (#1000000187) and propagated as recommended. Maxiprepped plasmids were digested with ScaI prior to transformation into electrocompetent EBY100 to improve the efficiency of homologous recombination. The transformed library was passed in SDCAA before being induced at 20 °C for 36–48 h. 2 × 10^7^ induced yeast cells per sort were washed three times with PBSA. For the screen against s14335, yeast cells were stained with anti-c-myc FITC (1:100) and 360 nM of biotinylated, His-Avi-tagged s14335 monobody in a 100 µL volume. For the screen against s19382, yeast cells were stained with anti-HA allophycocyanin (APC; 1:100; BioLegend) and 16 nM sfGFP-tagged s19382 monobody in a 520 µL volume. Cells were sorted as described previously. Following sorting, plasmids were miniprepped and prepared as before. Samples were submitted for either paired end 250 at Azenta (South Plainfield, NJ) or paired end150 at Neochromosome (Long Island City, NY) for NGS sequencing.

BBMerge^48^ (V39.01) was used to merge demultiplexed reads, Cutadapt^49^ (V2.6) was used to trim adapters, with a maximum error rate of 1/60. Custom Python scripts were used to filter and map barcodes, as well as to calculate enrichment scores^20,35^ from naive and enriched and for performing downstream analyses. Barcodes containing any per-base quality scores <30 (for paired-end 300 reads) or <40 (for paired-end 150 reads) were not considered.

### Cryo-EM sample preparation

A 3.5-fold molar excess of sfGFP-tagged monobody s19382 was added to SARS-CoV-2 Omicron prefusion stabilized Spike protein (Sino Biological Cat. V40589-V08H26) and incubated for overnight at 4 °C. The sample was concentrated and purified by size-exclusion chromatography on an AKTA Micro system using a Superose 6 Increase 3.2/300 column that had been pre-equilibrated in buffer containing 25 mM Tris pH 7.5, 150 mM NaCl. Fractions containing complex were pooled and concentrated to 1.8 mg/mL.

### Cryo-EM sample vitrification and data acquisition

Sample was vitrified by application of 3 µL onto a QuantiFoil Gold 1.2/1.3 300 mesh grid, which was blotted and plunge-frozen in liquid ethane using a Vitrobot Mk4 operated at 4 °C and 100% humidity (wait time 10 s, blot time 4 s, blot force 0). The vitrified sample was then imaged on a Titan Krios G3i electron microscope operated at 300 keV and equipped with a BioQuantum energy filter and a K3 Summit direct electron detector camera. Images were recorded at a 105,000x nominal magnification corresponding to a pixel size of 0.84 Å/pix using a 20 eV energy slit. Each image stack contained 40 frames with the dose fractionation on the specimen set to 1.31 electrons per Å^2^ for a total dose of 42.5 *e*^*-*^/Å^2^. Images were collected with a nominal defocus range of −0.8 to −2.8 µm.

### Cryo-EM data processing

All data were processed using CryoSPARC^62^. Raw movies were motion corrected, and their contrast transfer function (CTF) was estimated using patch motion correction and patch CTF estimation, respectively. Images were filtered to include only those with a detected fit resolution better than 5 Å. Initial particle picks using CryoSPARC’s blob picker were subjected to iterative rounds of reference-free 2D classification, and a subset of particles from the most well-defined 2D classes was used to generate two ab initio models with no symmetry imposed. One class contained Spike monomers that were primarily in the closed state, while the other contained Spike dimers with clear extra density at the dimer interface between the Spike RBDs. The full particle stack from all non-junk 2D classes was then subjected to iterative rounds of heterogeneous refinement using this dimeric ab initio model and several junk volumes as references. This cleaned particle stack was used to generate a second particle stack using cryoSPARC’s implementation of Topaz^63^. This stack was also subjected to iterative 2D classification and heterogeneous refinement, and both particle stacks were combined, removing duplicate particles. Non-uniform refinement was performed on the cleaned particle stack using the dimeric ab initio model as a reference. In this reconstruction, two RBDs from each spike protein were well resolved, with electron density corresponding to eight molecules of s19382 connecting the four RBDs. The third RBD from each spike protein was poorly resolved. In order to improve the resolution of the 4:8 RBD:s19382 dimer interface and understand the molecular nature of dimerization, a soft mask was generated around this region of the reconstruction. A second mask was then generated around the rest of the Spike dimer, and particle subtraction was performed to remove the signal not corresponding to the interface of interest. Local refinement was then performed to yield a final 3.16 Å map of the dimer interface (determined by the gold standard Fourier shell correlation threshold of 0.143). Map sharpening for model building was performed using EMReady^64^ v2.0 using default parameters and without specifying an input structure or mask.

### Model building and structural analysis

An initial model was generated in UCSF ChimeraX^65^ by rigid body fitting copies of the Omicron RBD from PDB 7U0N^66^ and a model of s19382 into the density. As expected, four RBD and eight s19382 protomers could be fit into the density. This initial model was subjected to iterative rounds of manual rebuilding in Coot^67^ and refinement in Phenix^68^, followed by molecular dynamics-assisted manual refinement with ISOLDE^69^ in ChimeraX and a final round of refinement in Phenix.

### Structure prediction with AlphaFold3

Structure predictions were generated using model weights and code released by Google DeepMind and Isomorphic Labs in early November 2024. For structure prediction of the s19382-RBD complex, 1000 seeds were predicted. For structure prediction of the 33,095 Sculptor designs, a single seed was predicted per target. In each case, five decoys were generated per seed. Top-scoring decoys across all seeds were selected for structure or metric analysis by taking the model(s) with the highest ‘ranking_score’. Pre-computed MSAs and templates (6YOR, 7L0N, 7MFU, 7OAQ) were used for RBD, identified via structure prediction of RBD with genetic and template search. MSAs and templates were similarly obtained for s19382 in s19382-RBD structure prediction runs. For the single-seed runs, the structures of 142 Sculptor-designed monobodies were first predicted using AlphaFold3 with genetic and template search enabled. Output MSAs were parsed to identify a set of 7250 unique Uniprot sequences, which were subsequently used to construct a reduced mmCIF database for monobody MSA generation and template search that significantly reduced genetic and template search times.

### Reporting summary

Further information on research design is available in the Nature Portfolio Reporting Summary linked to this article.

## Supplementary information

Supplementary Information

Reporting Summary

Transparent Peer Review file

## Source data

Source Data

## Data Availability

Designs targeting the Omicron BA.1 RBD were modeled using the structure deposited under PDB accession code 7TN0^44^ (). Relaxed models of this structure in complex with the designed monobodies were used throughout figures in this manuscript. The solved cryo-EM structure for s19382 bound to Omicron BA.1 RBD was deposited to the PDB under accession code 9Y5Y and the density map was deposited to the EMDB under accession code EMD-72524. pNVD006, the IVTT expression plasmid with C-terminal meGFP-tag used for expression with STAMMPPING, has been deposited on Addgene (Addgene ID: 229642, https://www.addgene.org/229642/). NGS data are available on the sequencing read archive under SRA accession code PRJNA1472999 (https://www.ncbi.nlm.nih.gov/bioproject/1472999). Processed affinity and kinetic measurements used in main text Figures are reproduced as Source Data; all other data (including quantified imaging data, fitted affinity and dissociation measurements, rosetta and AlphaFold3 metrics data) used to generate Supplementary Figs., Tables, and analyses are deposited as an Open Science Framework under accession code 7a8qg (https://osf.io/7a8qg/). Microfluidic wafer CAD design files, wafer fabrication protocols, and PDMS device fabrication protocols are freely available on the Fordyce lab website (www.fordycelab.com/microfluidic-design-files). CAD design files are also available on the Open Science Framework. Due to the large size of the imaging data, raw microscopy images are available upon request. Source data are provided with this paper.

## Code availability

Automation software associated with experimental and microscopy setups are available on Github (https://github.com/FordyceLab/RunPack) as well as on Zenodo^70^ (10.5281/zenodo.20948058). Software for processing images is available on Github (https://github.com/FordyceLab/ImageStitcher) as well as on Zenodo^71^ (10.5281/zenodo.20948118). Analytical software is available on Github (https://github.com/FordyceLab/ProcessingPack) as well as on Zenodo^72^ (10.5281/zenodo.20948138). Scripts used to generate Figures and Supplementary Figs. in this study are available as an Open Science Framework as well as on Zenodo^73^ (10.5281/zenodo.20483221).
