# Supplementary material for: ADAPT-M: a workflow for rapid, quantitative in vitro measurements of enriched protein libraries
Source: Nat Commun. 2026 Jul 15;17:8673. doi: 10.1038/s41467-026-75463-1 (PMC13490508; doi:10.1038/s41467-026-75463-1)
Supplement: Supplementary file 1 [file 41467_2026_75463_MOESM1_ESM.pdf]

Supplementary Note 1:

In FACS experiments, cells are sequentially labeled, first with biotinylated Omicron BA.1 receptor binding domain (RBD) and then with streptavidin (SA), with a wash in between to remove free RBD. This kind of sequential labeling is not possible for STAMMPPING experiments because of the microfluidic device's surface chemistry. Open neutravidin (NA) sites on the device (if biotinylated bovine serum albumin (bBSA) passivation was incomplete) could compete with immobilized monobody for binding to biotinylated-RBD. Additionally, false-positive binding signal could occur from the fluorescently labeled avidin probe binding accessible bBSA confounding accurate K_d determination. Therefore we reasoned the target protein must be pre-incubated with the avidin probe prior to introduction onto the device.

We first sought to use an iFluor 650 monomeric streptavidin conjugate (mSA650) to prevent avidity effects. However, we observed significant non-specific binding to empty chambers, meGFP-containing chambers, and to surfaces outside of the button region. This suggested at least one of three issues: (1) incomplete binding of RBD to mSA650 during incubation, (2) dissociation of RBD from mSA650 over the course of the measurements, and/or (3) excess open biotin sites on the device surface. To address these issues we (1) extended incubation of RBD-mSA650 to 40 hours, well over five half-lives recommended for proper binding equilibration, (2) simultaneously performed experiments using a standard, tetrameric NA-Dylight 650 (NA650) probe, which offers slower dissociation rates than mSA, and (3) experimented with increased blocking times to minimize available biotin sites, respectively. Increasing the length of blocking to two hours resulted in the largest reduction of non-specific binding, and swapping to a NA650 probe further improved selective patterning of button surfaces. Even so, overnight NA blocking with buttons open was necessary to reduce non-specific binding to acceptable levels.

While this reduced non-specific binding of the NA probe, leaving the buttons open while flowing NA overnight resulted in minor cross-contamination across chambers, as assessed from meGFP expression levels in empty chambers. To minimize the effects of cross-contamination, we implemented a very conservative expression threshold. As a result, measured affinities are primarily driven by the printed designs, not by any contaminating designs. Furthermore, since many replicates are printed across the device, each replicate is surrounded by different neighboring sequences, and any artifacts from small amounts of contamination average out across the replicates reducing their impact on the overall measurements.

Given the non-specific binding to "empty" chambers, we applied a t-test to compare cy5 fluorescence intensities in aggregated chambers for each design against those of all empty chambers. Designs with $p > 0.05$ were classified as binding below the limit of detection, and apparent dissociation constants ($K_{d,\ app}$) were reported for all other designs. The dynamic range of measured $K_{d,app}$s was limited, spanning 82 nM to 1.1 µM, with poor resolution among binders in the ~100 nM range. We observed expression above the expression threshold for 50/51 printed designs. All designs that showed detectable binding in this set of measurements were selected for validation with a monovalently labeled target (Figure 2f). Additionally, a subset of designs that appeared to bind below the limit of detection were included as negative controls, and to determine whether binding could be measured with an improved limit of detection (Supplementary Figure S10).

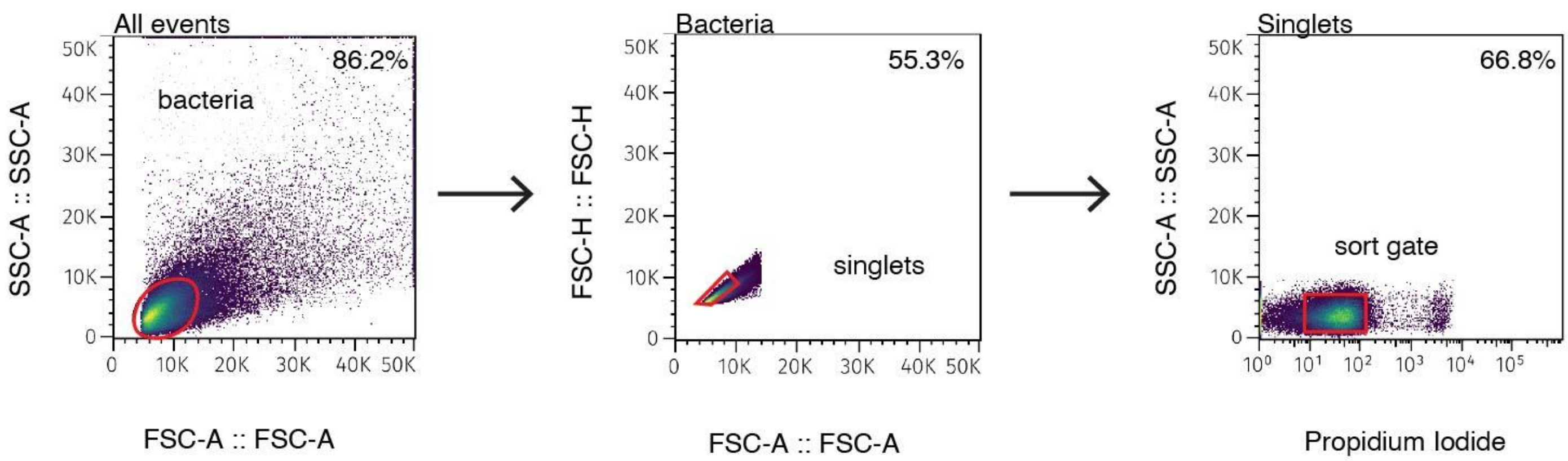

Supplementary Figure S1. FACS plots showing sorting strategy for isolating single bacterial cells on a Sony SH800. Bacterial cells were stained with PI prior to sorting to distinguish live/dead cells. Left: Bacterial cells were first gated by SSC and FSC. Center: FSC-H vs FSC-A was used as a gate for singlets. Right: PI^- cells were selected for sorting into individual wells of a multiwell plate and outgrowth. Approximate gates are outlined in red.

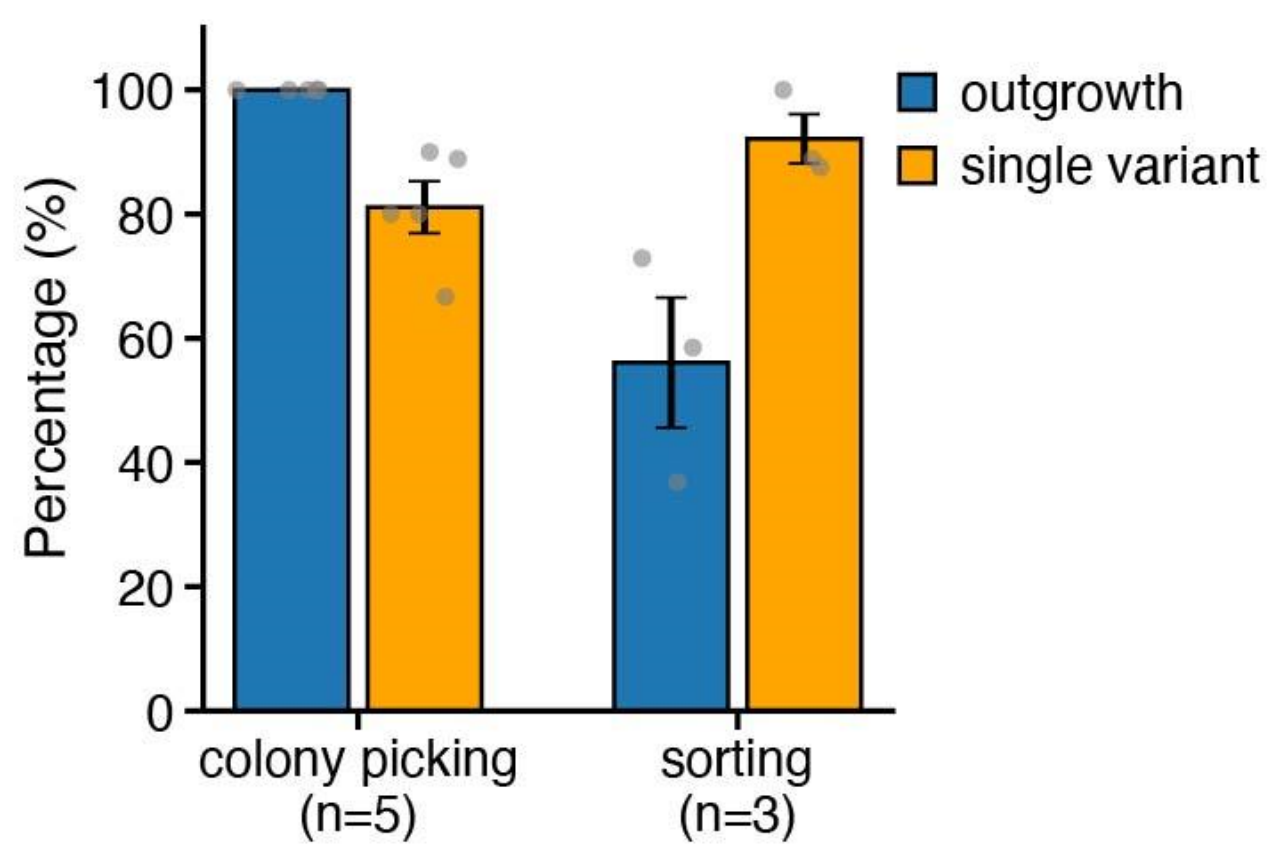

Supplementary Figure S2. Bar plots showing cell outgrowth and single variant isolation rates for manual colony picking versus single cell sorting. Data represent mean values; error bars indicate standard error from biological replicates.

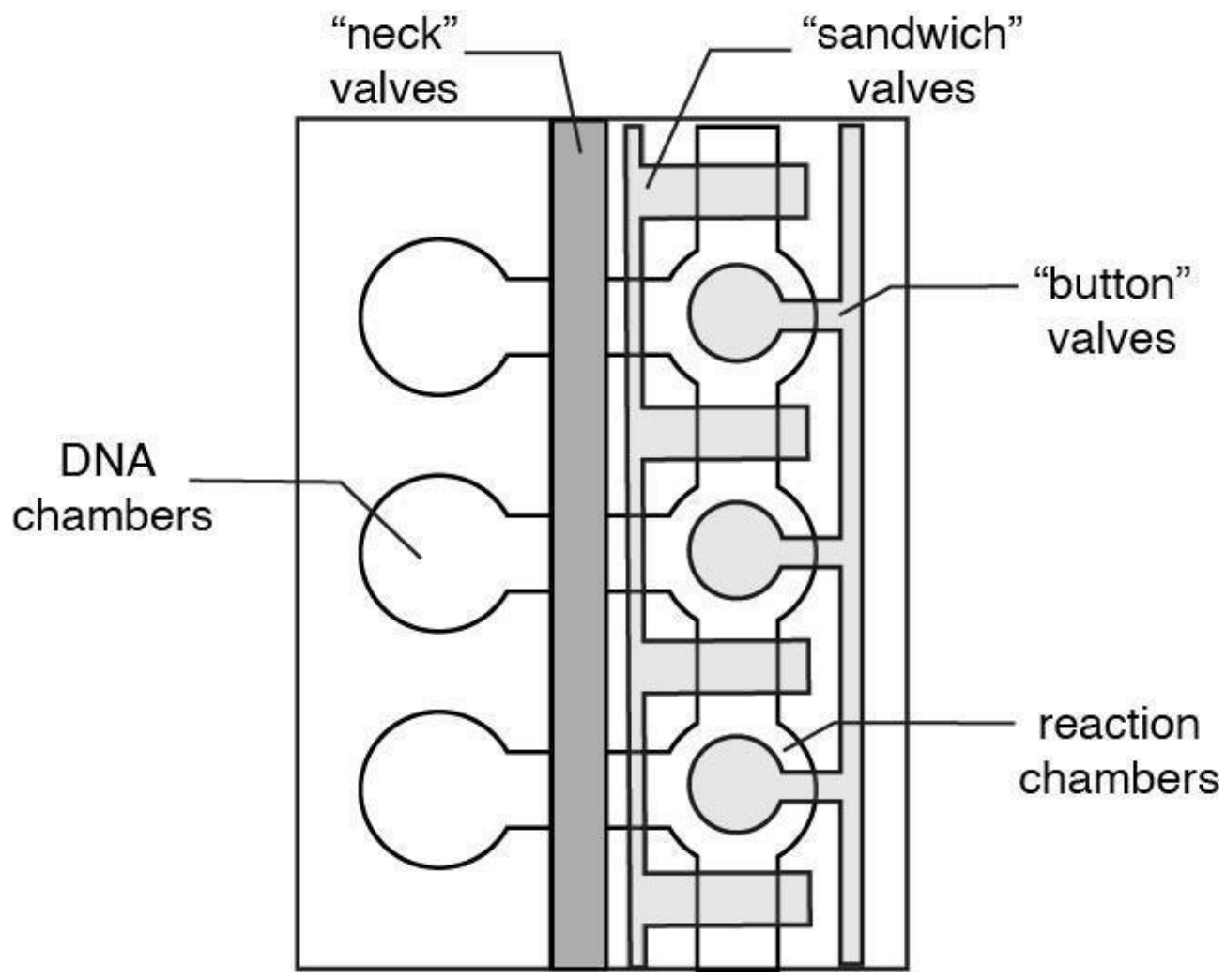

Supplementary Figure S3. Cartoon schematic of chambers and valves on the microfluidic device. Putative binders are expressed in DNA chambers that are precisely aligned to linear expression templates during device bonding. These DNA chambers are separated from reaction chambers by "neck" valves, where putative binders are immobilized and binding is surveyed. "Sandwich" valves separate reaction chambers from one another to prevent cross-chamber contamination, and "button" valves permit both selective surface patterning of reaction chambers with anti-GFP nanobodies and protection of binding interactions during imaging.

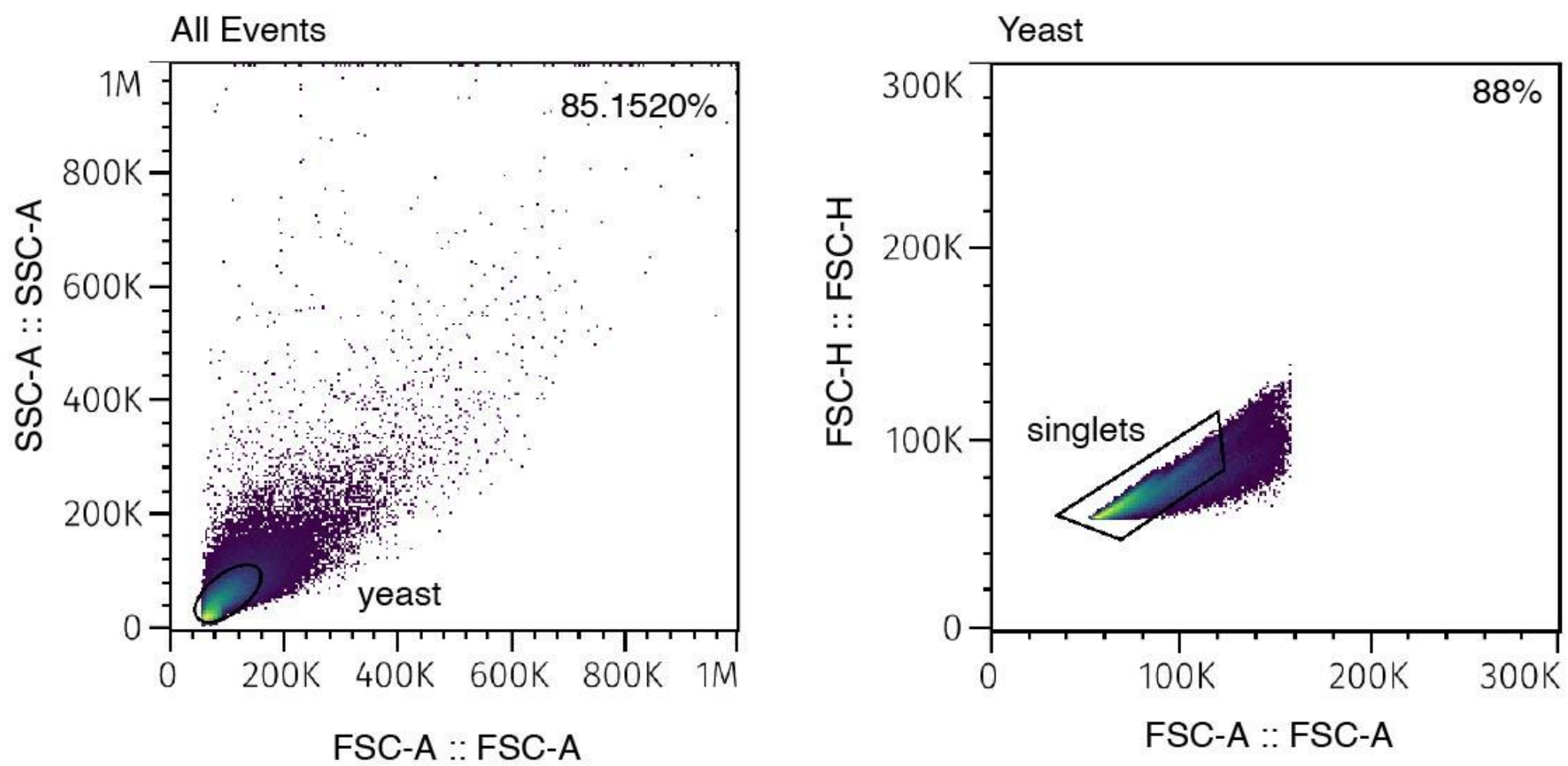

Supplementary Figure S4. Sample FACS gating strategy for isolating singlet yeast cells for yeast surface display experiments. Left: Yeast cells were first gated by SSC and FSC. Right: FSC-H vs FSC-A was used to gate for singlet yeast cells. Approximate sort gates are shown in black. Gating for singlets was performed before selection of binding populations. Selection in this manner was performed for all yeast sorting experiments throughout this manuscript.

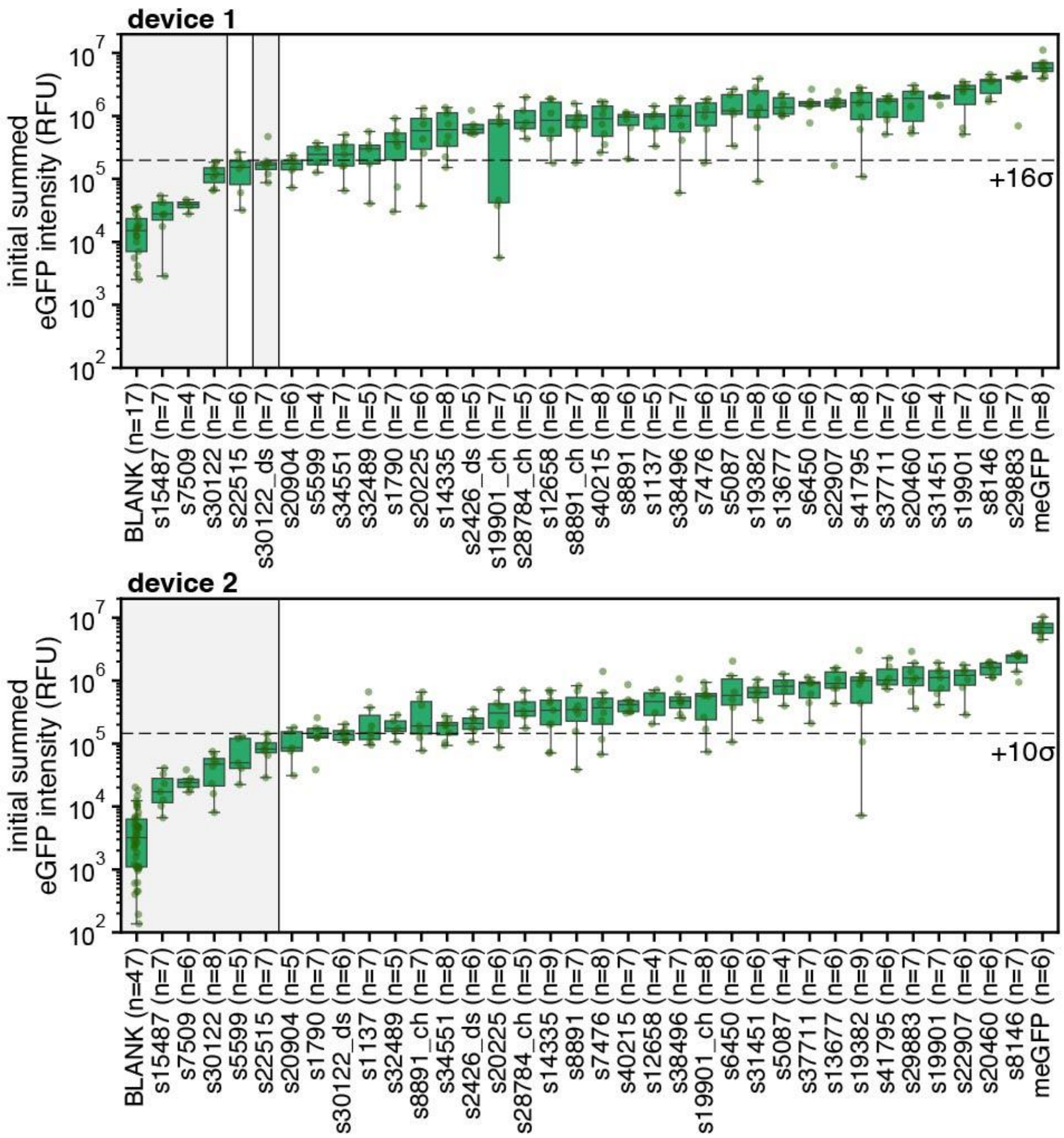

Supplementary Figure S5. Boxplots showing initial background-subtracted meGFP intensities across all replicates for each monobody design tested. Dashed black line represents the meGFP expression threshold, set as the annotated number of standard deviations above the mean meGFP intensity across blank chambers for each device. Replicates with meGFP intensities below the threshold were not used for K_d estimation. Grey shading indicates designs without at least 2 replicates expressing above this threshold. Box plots show the median, interquartile range (25th–75th percentiles), and whiskers extending to 1.5× IQR. Source data are provided as a Source Data file.

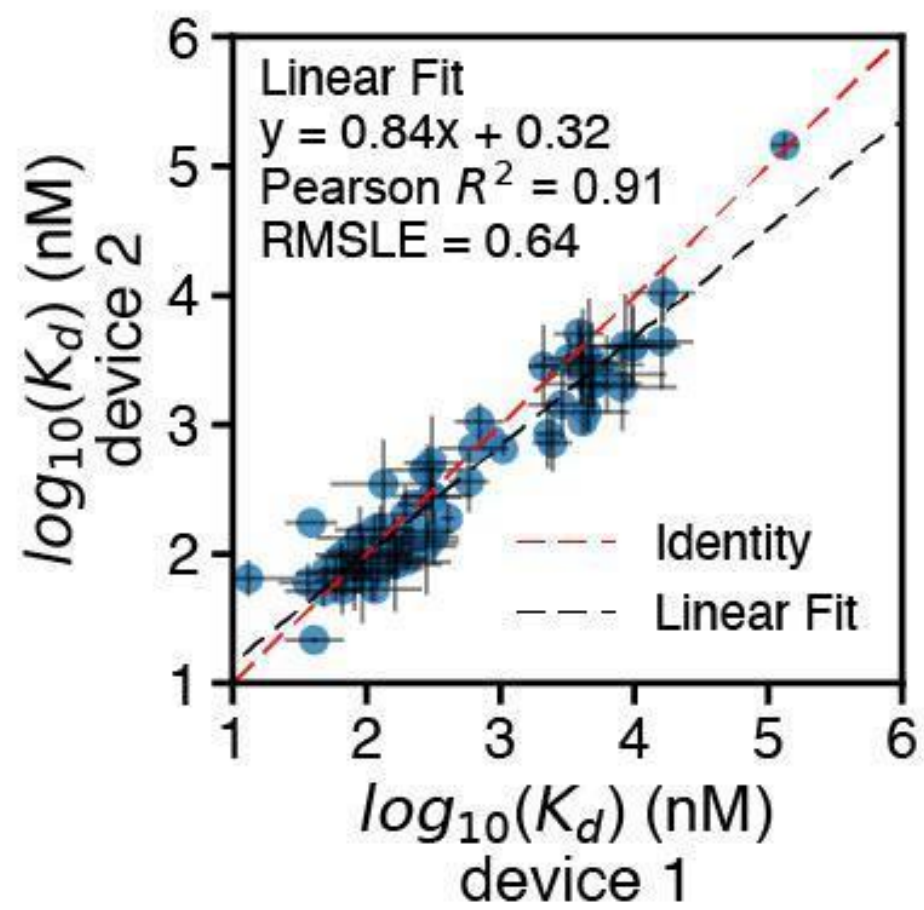

Supplementary Figure S6. Pairwise comparison of per variant K_ds for all designs with binding above the limit of detection (n=74) across 2 experiments. Points indicate median affinities (± SD) for each design. Dashed red line indicates the identity line (1:1) and the dashed black line represents the best fit linear regression. Source data are provided as a Source Data file.

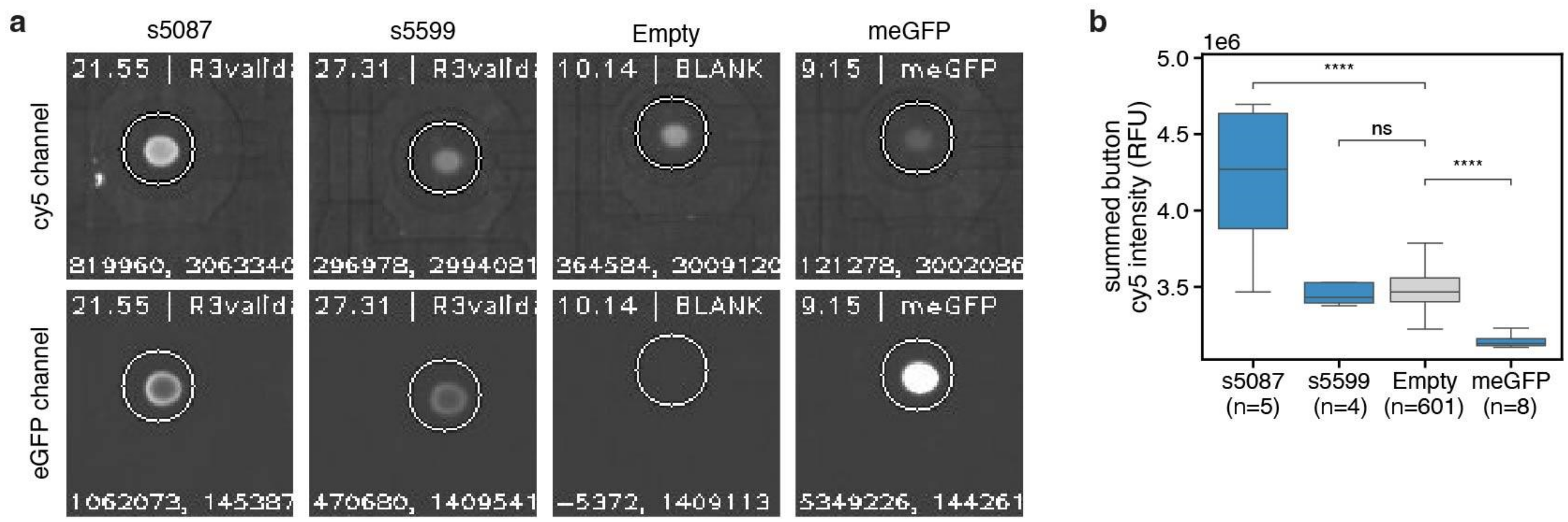

Supplementary Figure S7. Defining experimental limit of detection. **a**. Images showing measured cy5 intensities (top) and meGFP intensities (bottom) for a weakly binding (s5087) monobody-containing chamber and monobody-containing chambers with binding below the limit of detection (s5599), along with corresponding intensities for meGFP-only and empty chambers. Contrast is uniformly enhanced for optimal visibility. Empty chambers were used to calculate the limit of detection for monobody-containing chambers, providing a strict binding threshold since meGFP-only containing chambers typically bind much more weakly than empty chambers. **b**. Distribution of summed button cy5 intensities at the highest RBD concentration for meGFP-containing and empty chambers along with weakly binding (s5087) and nonbinding (s5599) monobody-containing chambers. Box plots show the median, interquartile range (25th–75th percentiles), and whiskers extending to 1.5× IQR. A two-sided t-test was applied, **** denotes $p < 0.0001$, ns denotes $p > 0.05$. P-values (left to right): p = 1.576e-11, p=9.595e-01, p=3.631e-24). Source data are provided as a Source Data file.

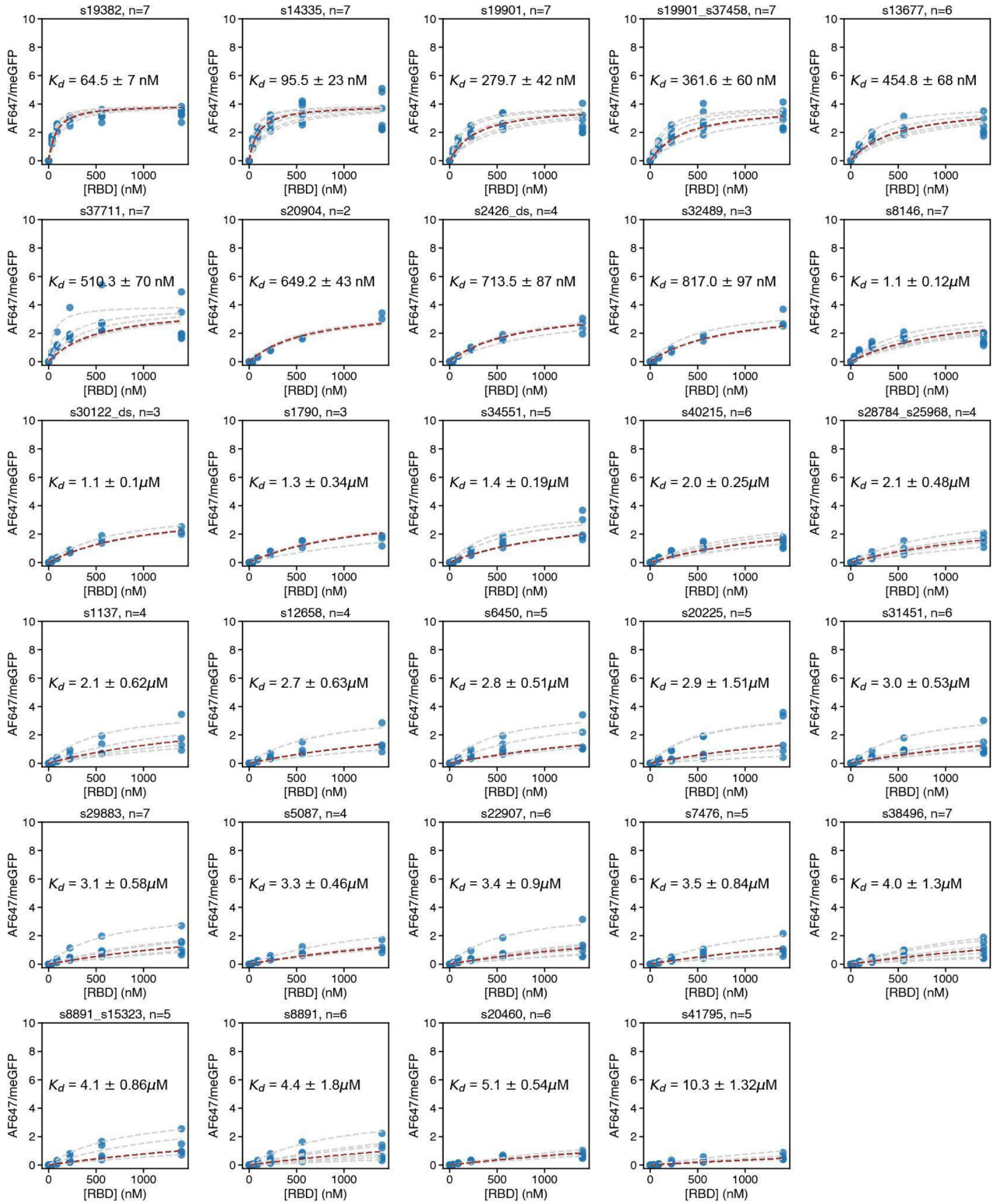

Supplementary Figure S8. Concentration-dependent binding curves for monobody library measured against monovalent, SpyTag/Catcher-AF647-labeled Omicron/BA.1 RBD. Light grey dashed lines indicate per-chamber Langmuir isotherm fits. The median K_d is annotated, and the dashed red line indicates the fit returning the median K_d. Blue markers indicate measured intensity ratios at each RBD concentration. Source data are provided as a Source Data file.

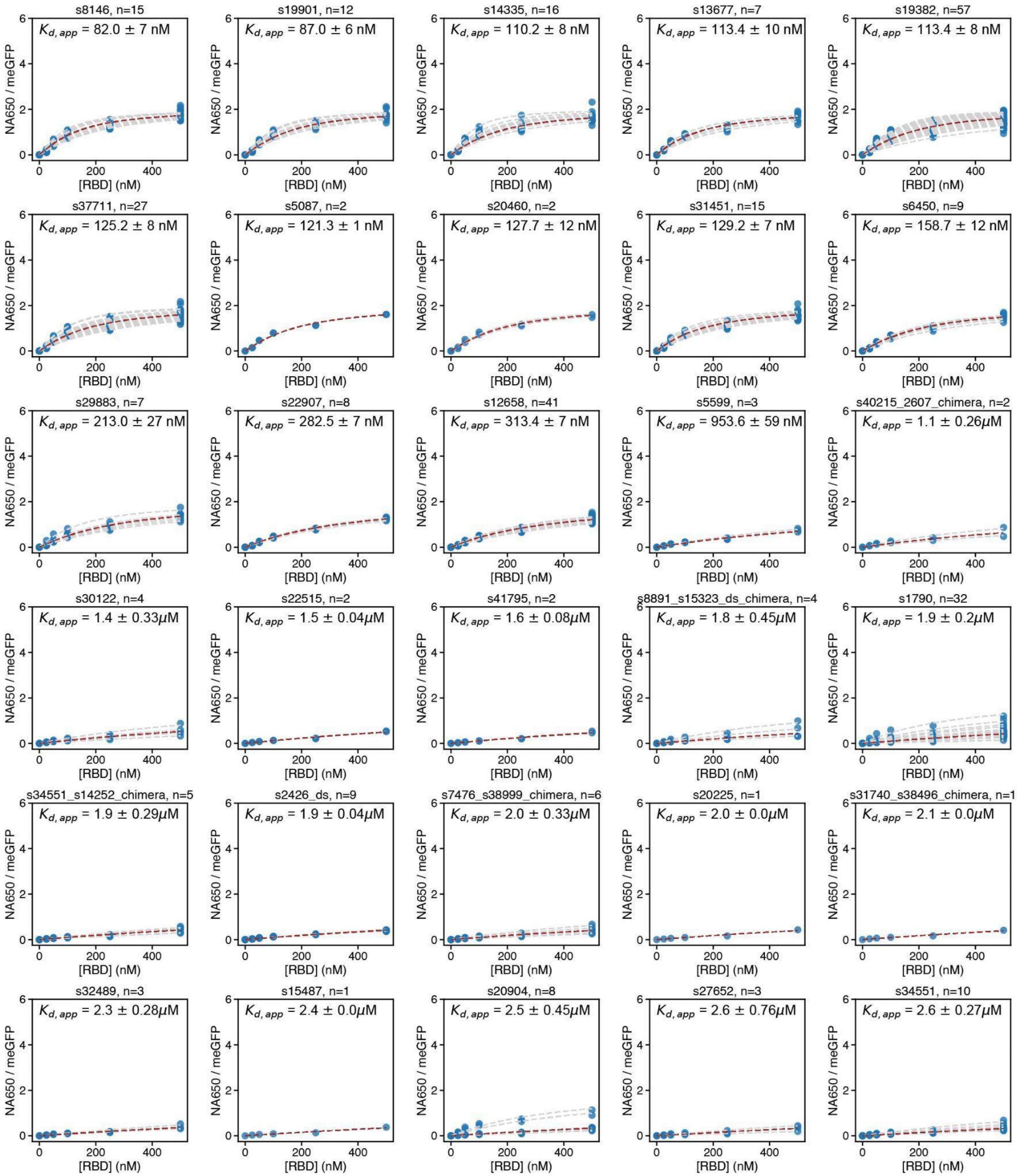
s8146, n=15
K_{d, app} = 82.0 ± 7 nM
s19901, n=12
K_{d, app} = 87.0 ± 6 nM
s14335, n=16
K_{d, app} = 110.2 ± 8 nM
s13677, n=7
K_{d, app} = 113.4 ± 10 nM
s19382, n=57
K_{d, app} = 113.4 ± 8 nM
s37711, n=27
K_{d, app} = 125.2 ± 8 nM
s5087, n=2
K_{d, app} = 121.3 ± 1 nM
s20460, n=2
K_{d, app} = 127.7 ± 12 nM
s31451, n=15
K_{d, app} = 129.2 ± 7 nM
s6450, n=9
K_{d, app} = 158.7 ± 12 nM
s29883, n=7
K_{d, app} = 213.0 ± 27 nM
s22907, n=8
K_{d, app} = 282.5 ± 7 nM
s12658, n=41
K_{d, app} = 313.4 ± 7 nM
s5599, n=3
K_{d, app} = 953.6 ± 59 nM
s40215_2607_chimera, n=2
K_{d, app} = 1.1 ± 0.26μM
s30122, n=4
K_{d, app} = 1.4 ± 0.33μM
s22515, n=2
K_{d, app} = 1.5 ± 0.04μM
s41795, n=2
K_{d, app} = 1.6 ± 0.08μM
s8891_s15323_ds_chimera, n=4
K_{d, app} = 1.8 ± 0.45μM
s1790, n=32
K_{d, app} = 1.9 ± 0.2μM
s34551_s14252_chimera, n=5
K_{d, app} = 1.9 ± 0.29μM
s2426_ds, n=9
K_{d, app} = 1.9 ± 0.04μM
s7476_s38999_chimera, n=6
K_{d, app} = 2.0 ± 0.33μM
s20225, n=1
K_{d, app} = 2.0 ± 0.0μM
s31740_s38496_chimera, n=1
K_{d, app} = 2.1 ± 0.0μM
s32489, n=3
K_{d, app} = 2.3 ± 0.28μM
s15487, n=1
K_{d, app} = 2.4 ± 0.0μM
s20904, n=8
K_{d, app} = 2.5 ± 0.45μM
s27652, n=3
K_{d, app} = 2.6 ± 0.76μM
s34551, n=10
K_{d, app} = 2.6 ± 0.27μM
NA650 / meGFP
[RBD] (nM)

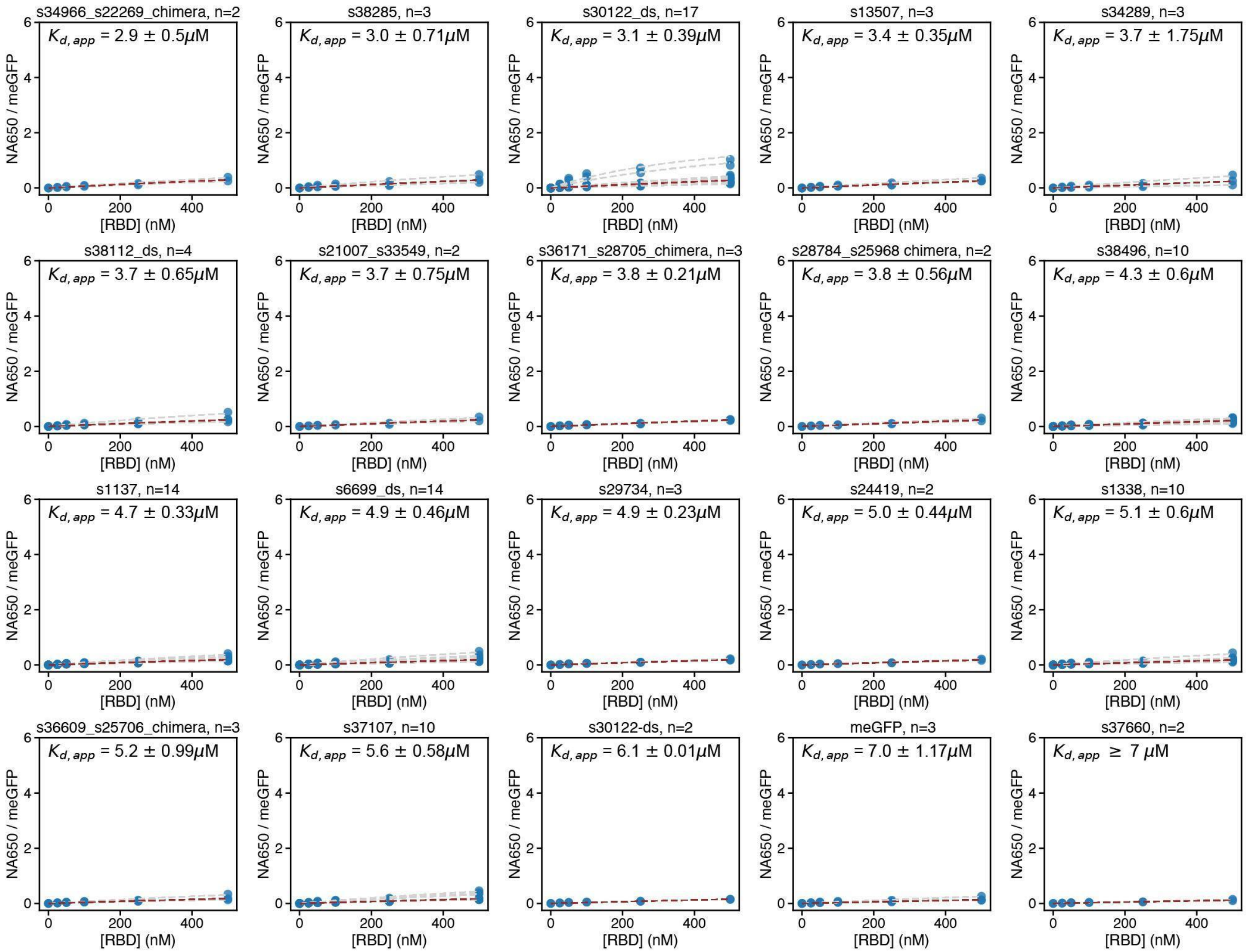

Supplementary Figure S9. Concentration-dependent binding curves for monobody library measured against a tetrameric, neutravidin-iFluor650-labeled RBD. Light grey dashed lines indicate per-chamber Langmuir isotherm fits. The median $K_{d,app}$ is annotated, and the dashed red line indicates the fit returning this $K_{d,app}$. Blue markers indicate measured intensity ratios at each RBD concentration. Source data are provided as a Source Data file.

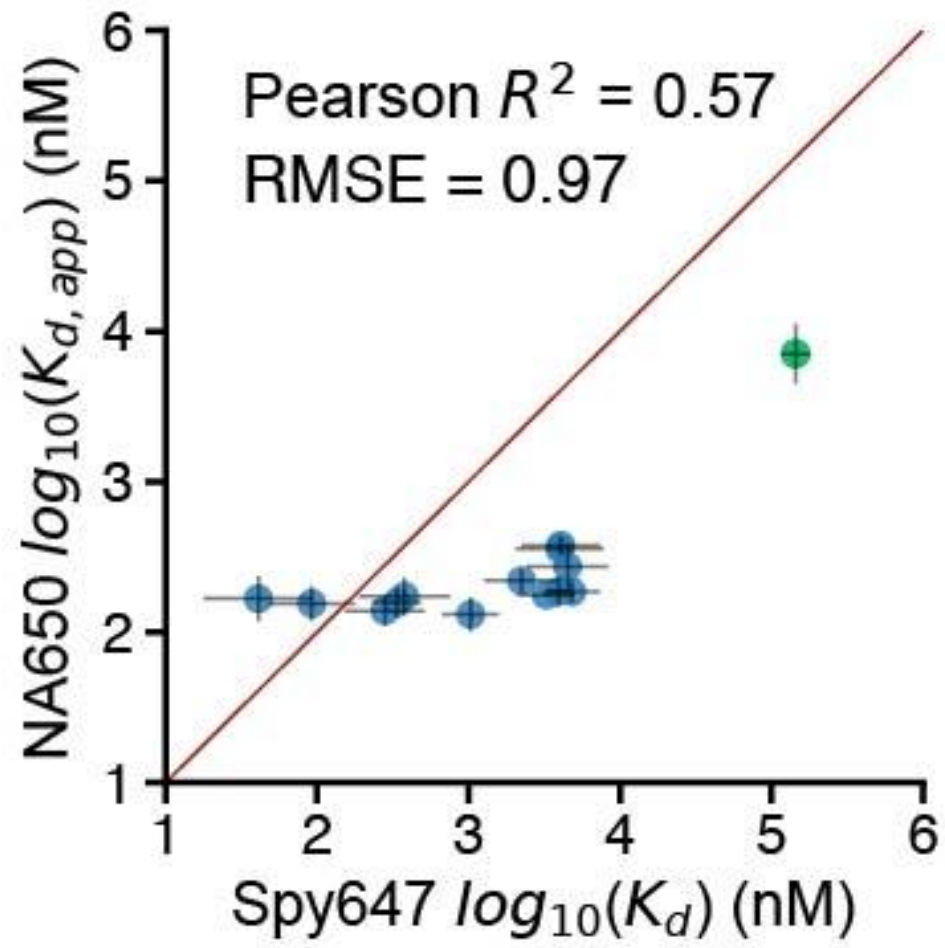

Supplementary Figure S10. K_d values of avid neutravidin-based STAMMPPING measurements are compressed compared to measurements made with non-avid Spy-647 labeled Omicron BA.1 RBD. Data are presented as median values +/- SD. The solid red line indicates identity line (y=x). Source data are provided as a Source Data file.

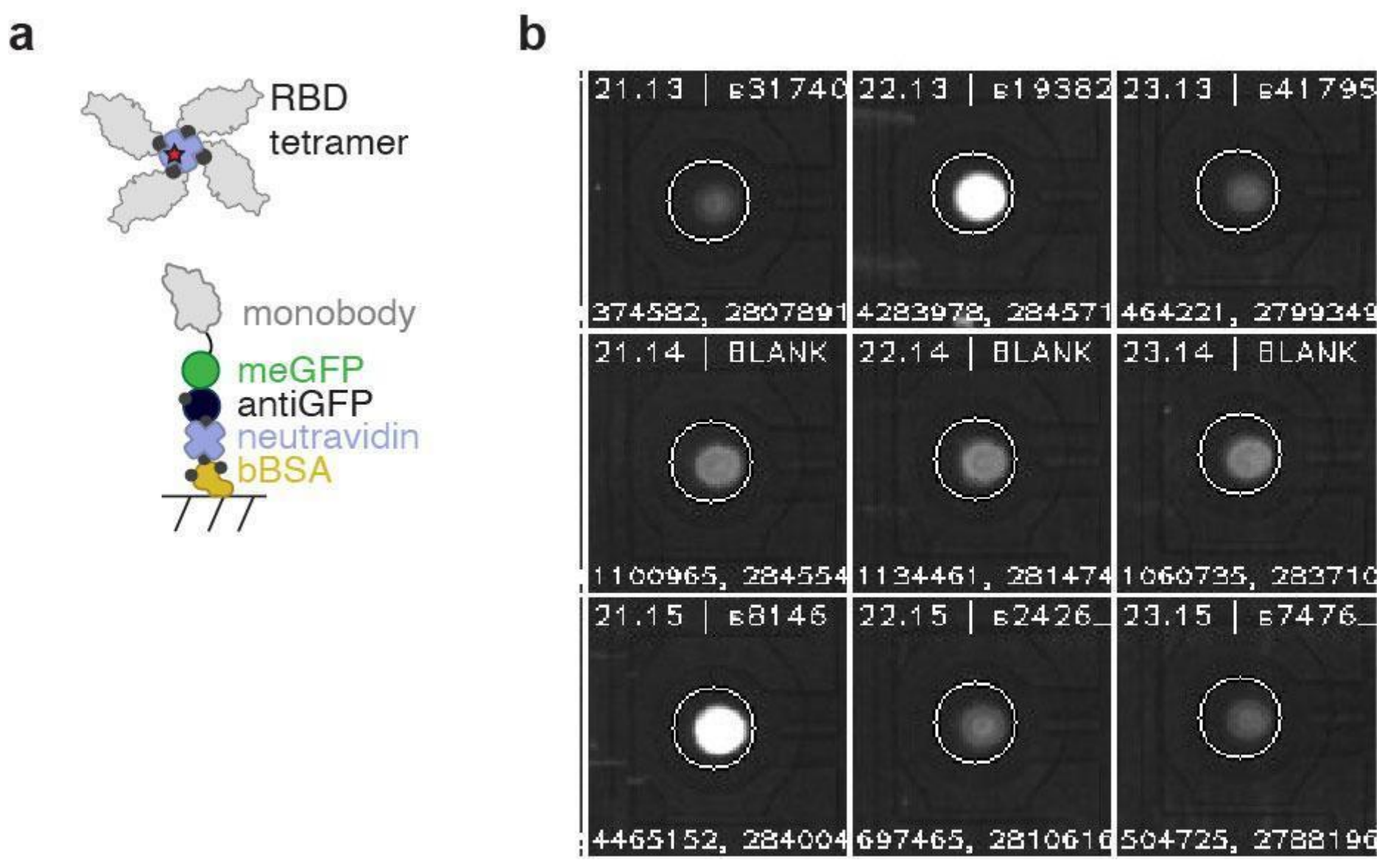

Supplementary Figure S11. Images showing cy5 intensities for subset of chambers across microfluidic device using neutravidin-647 RBD target.

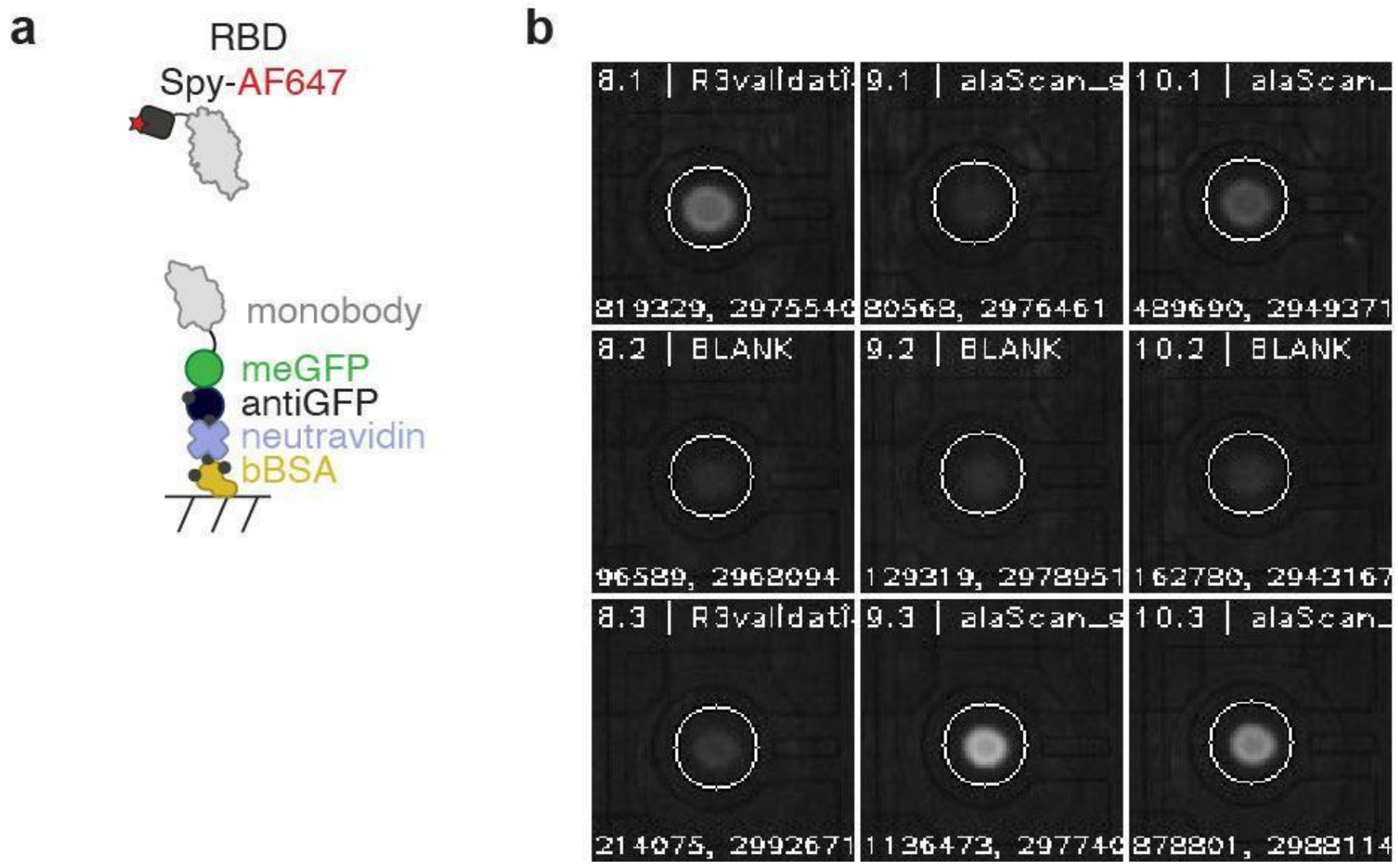

Supplementary Figure S12. Images showing cy5 intensities for subset of chambers across microfluidic device using AF-647 SpyTag RBD target.

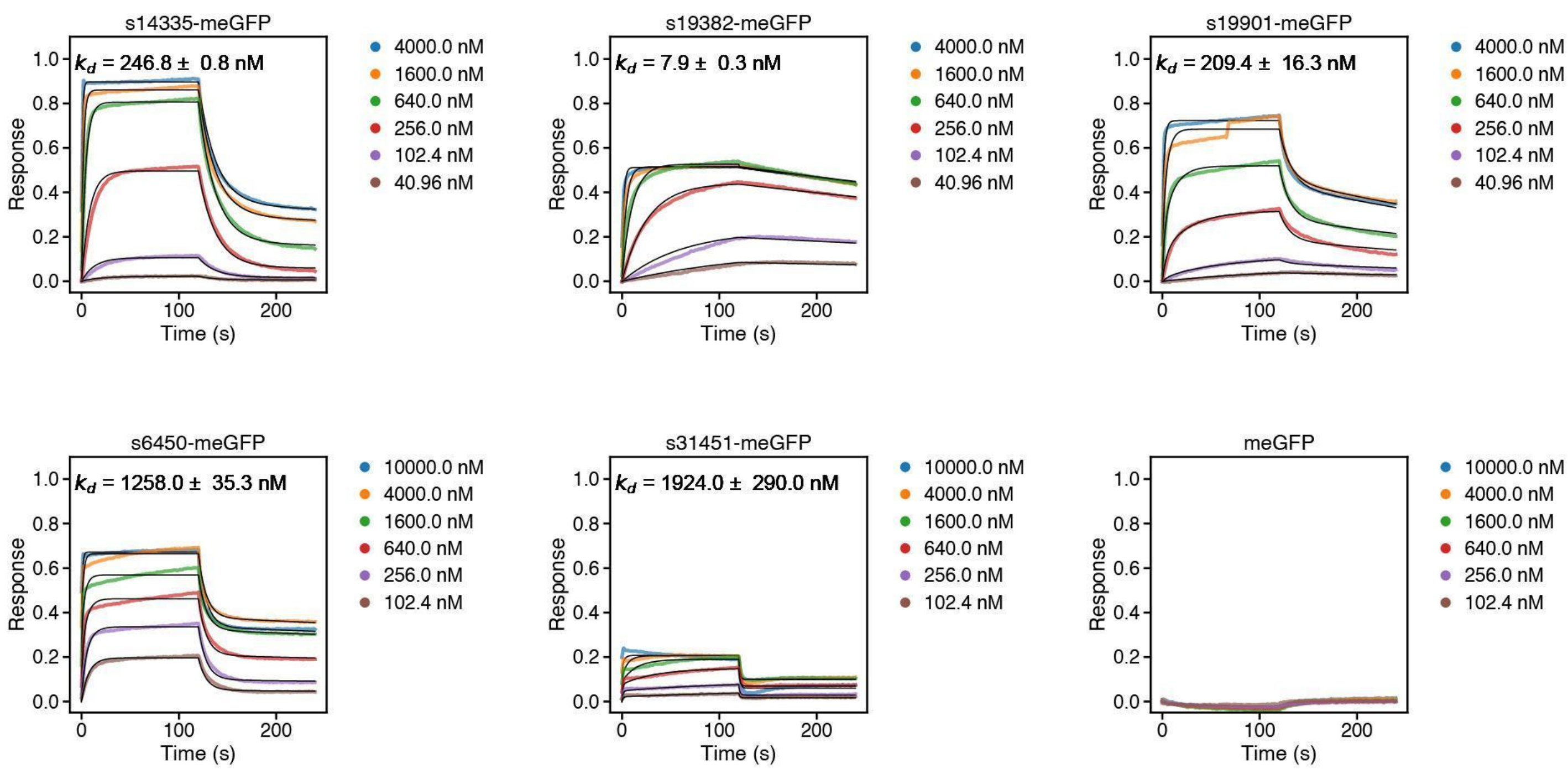

Supplementary Figure S13. Kinetic binding traces and global kinetic fits measured by Octet BLI for designed binders against Omicron BA.1 RBD. Source data are provided as a Source Data file.

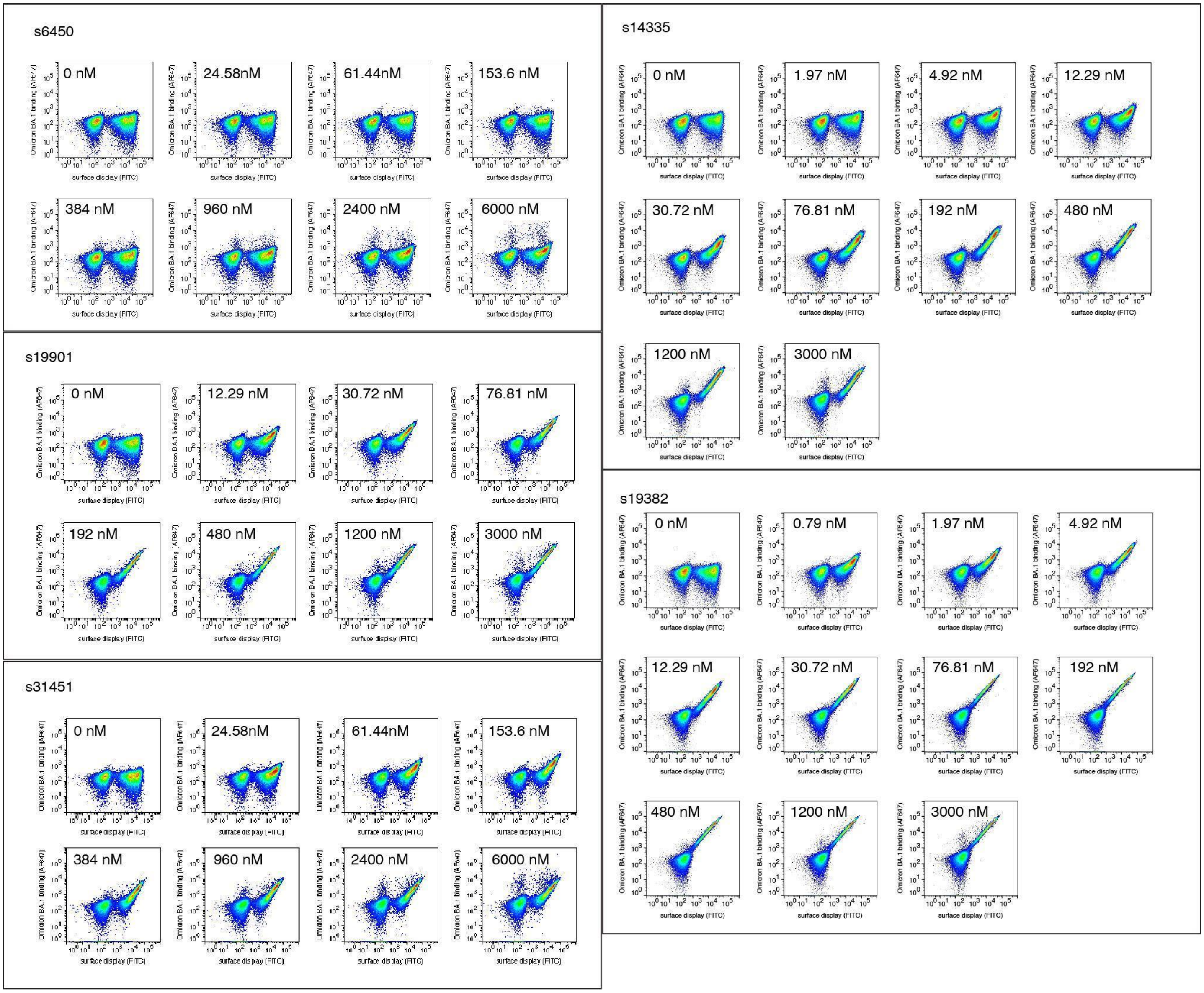

Supplementary Figure S14. FACS plots from one replicate of titration series for 5 designs. Staining concentrations are annotated. Plots display singlet-gated cell populations (see Supplementary Fig. 4 for the gating strategy).

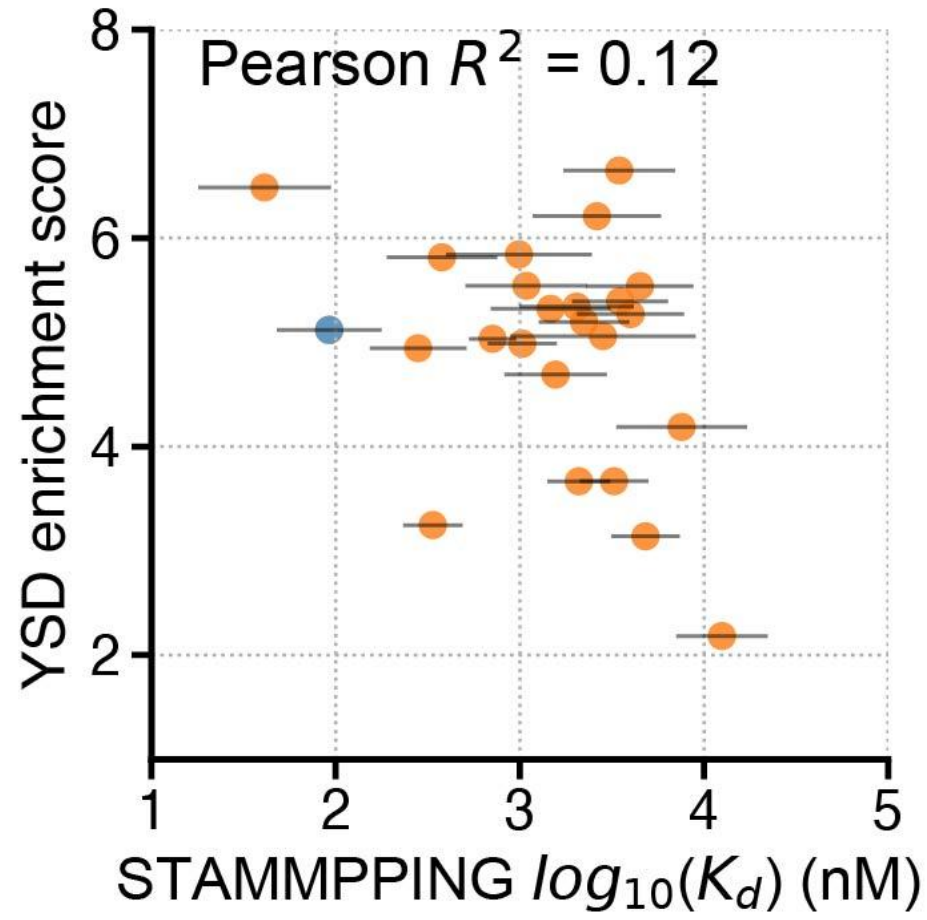

Supplementary Figure S15. Log enrichment scores from yeast surface display plotted as a function of K_d measured via STAMMPPING using an Omicron BA.1 RBD Spy647 antigen. Individual designs represented as orange scatter points; s14335 shown in blue. Data are presented as median values +/- SD. Source data are provided as a Source Data file.

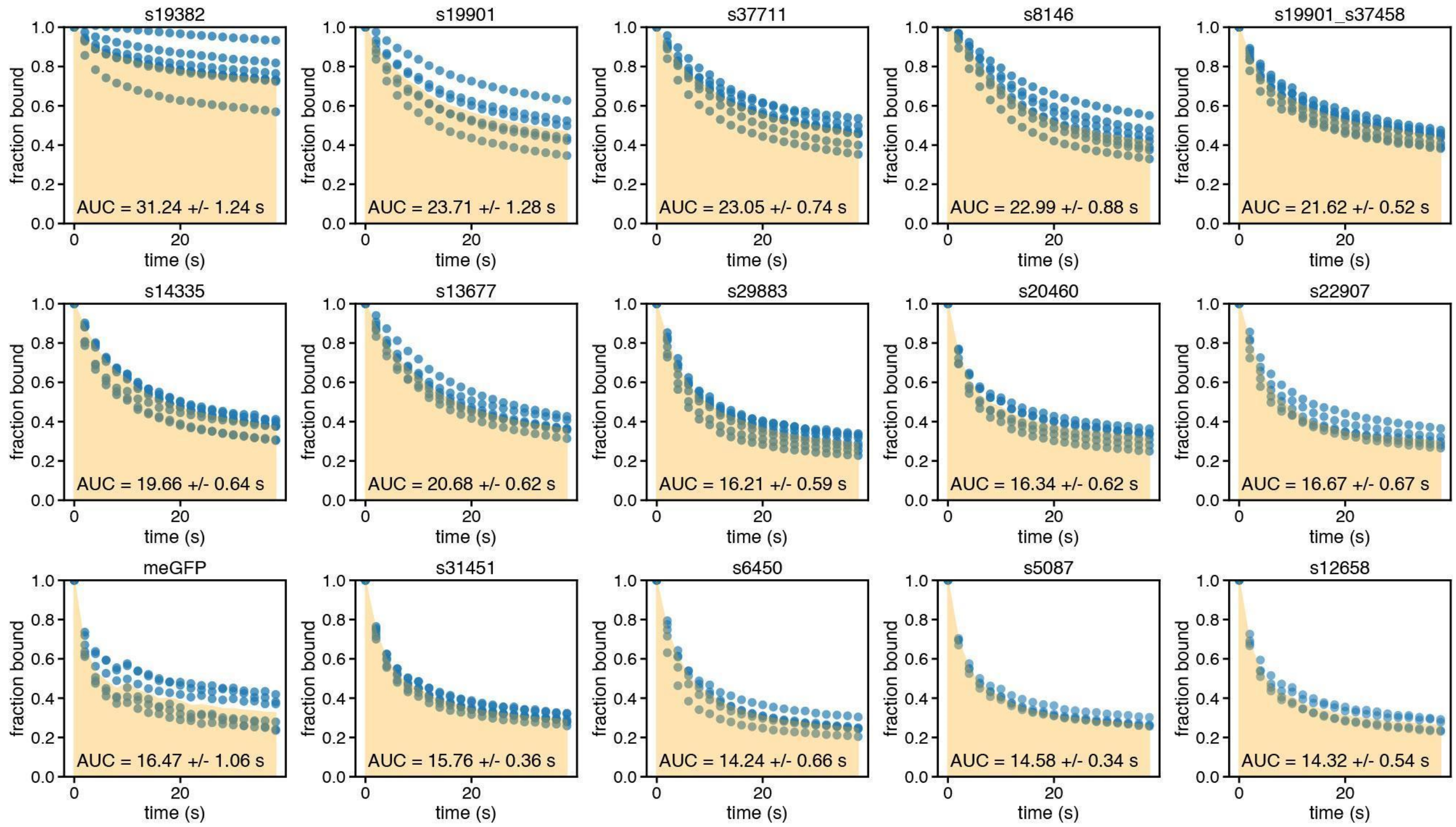

Supplementary Figure S16. Time-dependent dissociation curves for monobody library measured against Omicron RBD via STAMMPPING. Blue markers indicate normalized, measured AF647 intensities at each time point. AUC for median dissociation at each time point is shaded in orange for each designed binder. Median AUCs (± SEM) are annotated. Source data are provided as a Source Data file.

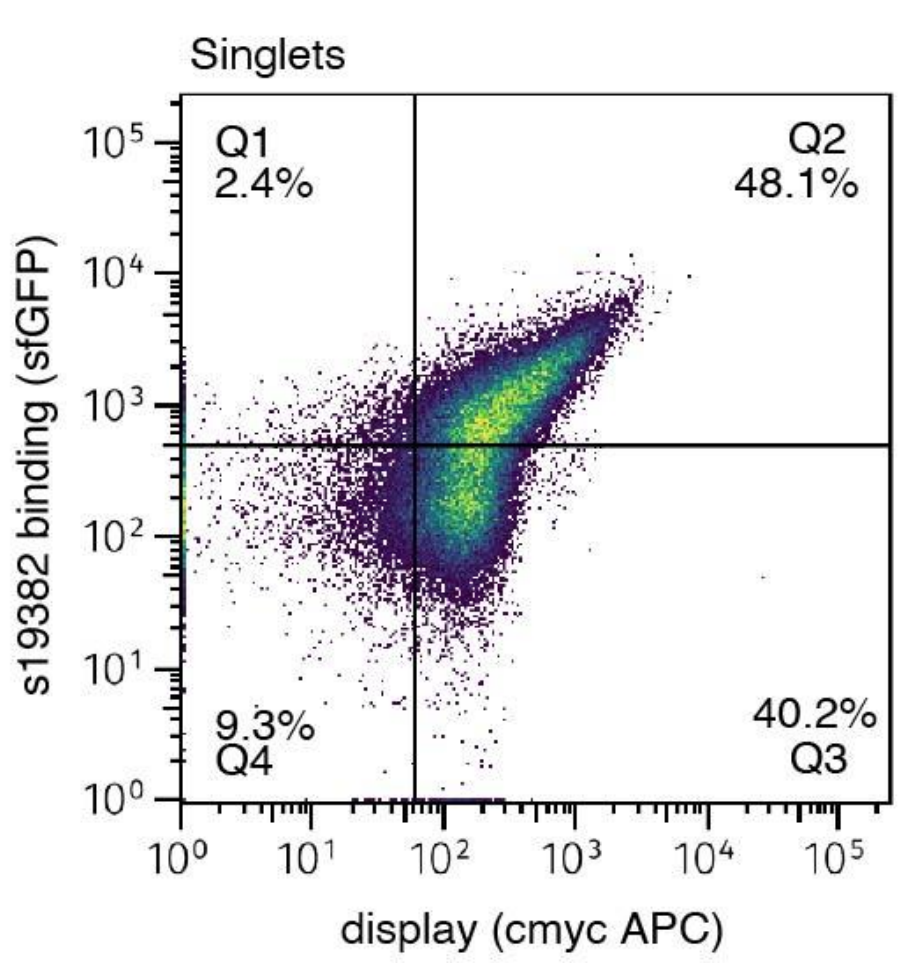

Supplementary Figure S17. FACS plots showing representative flow cytometry dot plots of Omicron BA.1 SSM yeast surface display screening against s19382. Approximate gates are outlined in black. Plots display singlet-gated cell populations (see Supplementary Fig. 4 for the gating strategy). 1000nM sfGFP-tagged s19382 was used as a primary stain, and anti-HA APC was used to detect the displaying Omicron BA.1 population. Target staining concentrations are indicated. Q2 represents the approximate sort gate.

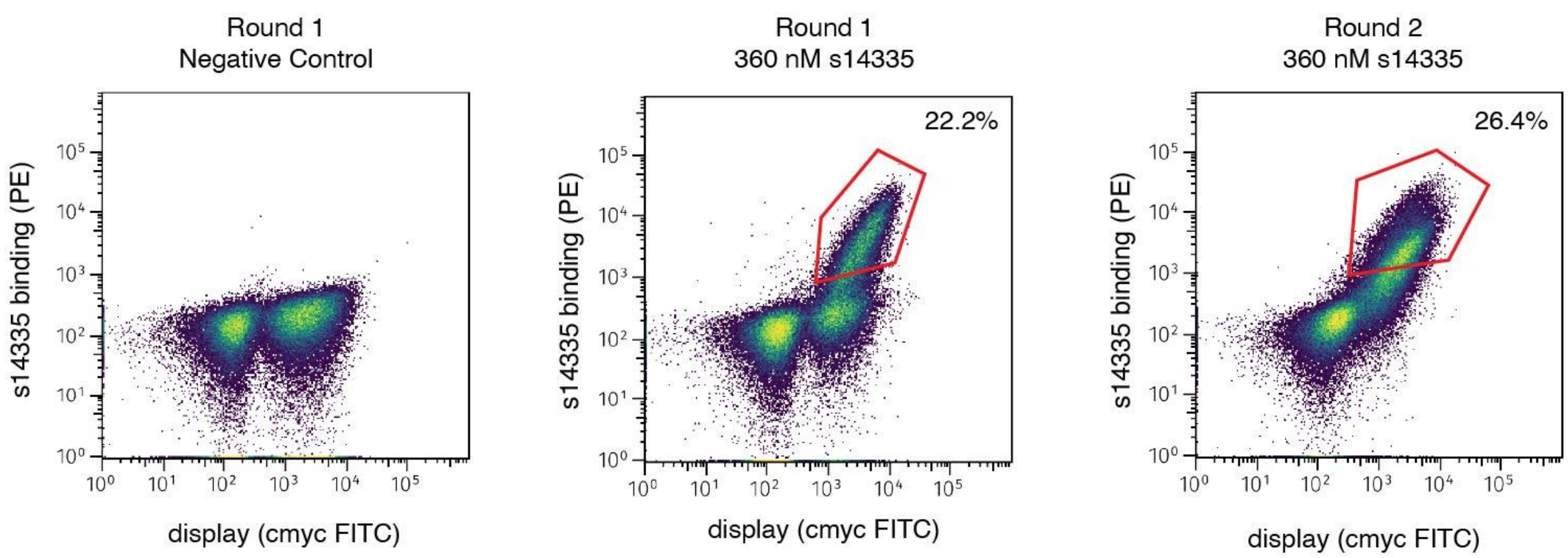

Supplementary Figure S18. Representative flow cytometry dot plots of Omicron BA.1 SSM yeast surface display screening binding to s14335. Yeast labeled with secondary stains and without any putative binder were used as a negative control to set binding gates (left). His- Avi-tagged s14335 was biotinylated and used as a primary stain, streptavidin-PE served as a secondary for detection of s14335-bound cells. Anti-cmyc FITC was used to detect the displaying Omicron BA.1 population. Target staining concentrations are indicated. Approximate sort gates for 2 iterative rounds of enrichment (center, right) are outlined in red. Plots display singlet-gated cell populations (see Supplementary Fig. 4 for the gating strategy).

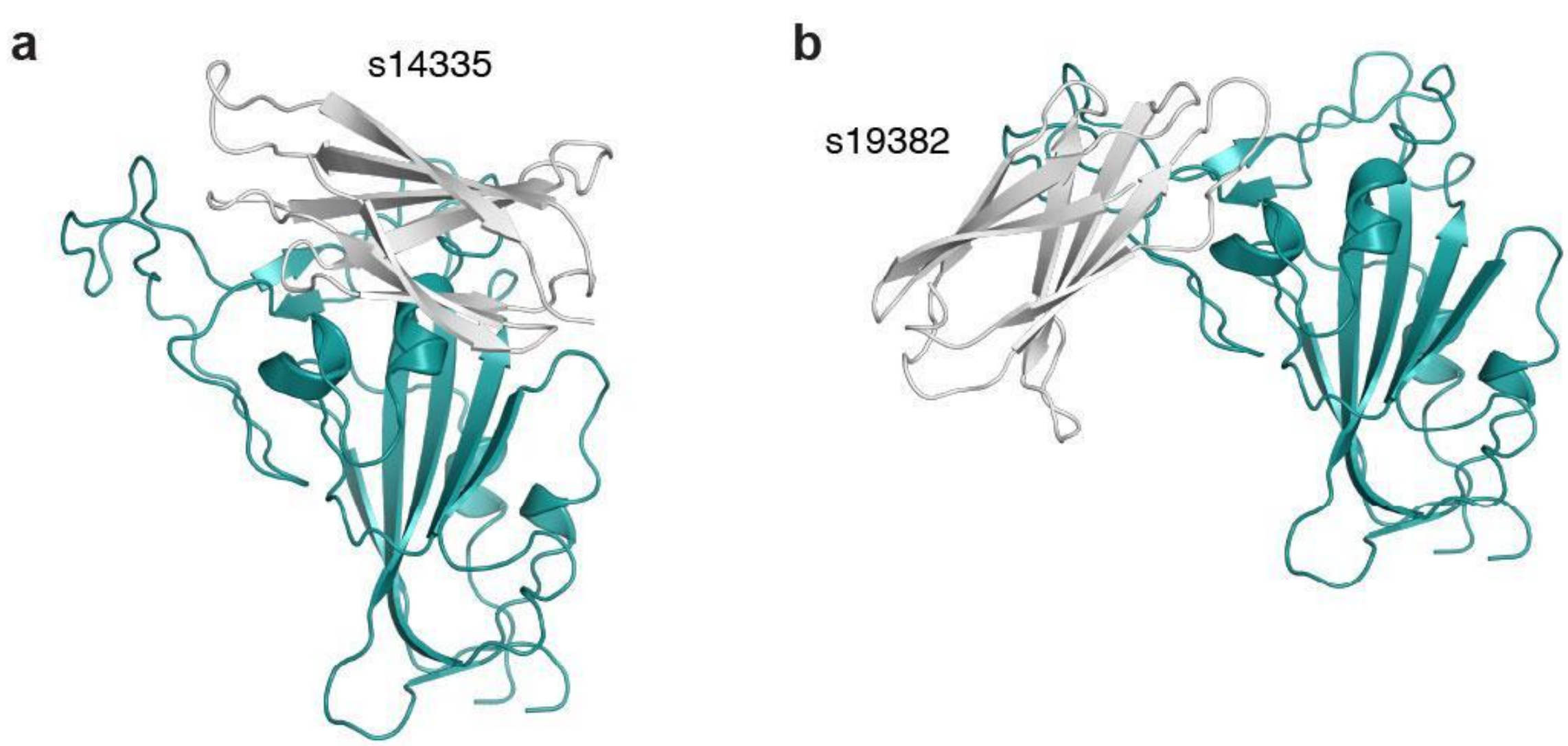

Supplementary Figure S19. Design models demonstrating RBD-monobody binding modes. **a** s14335 and **b** s19382 design models (grey, labeled) binding to Omicron BA.1 RBD (teal).

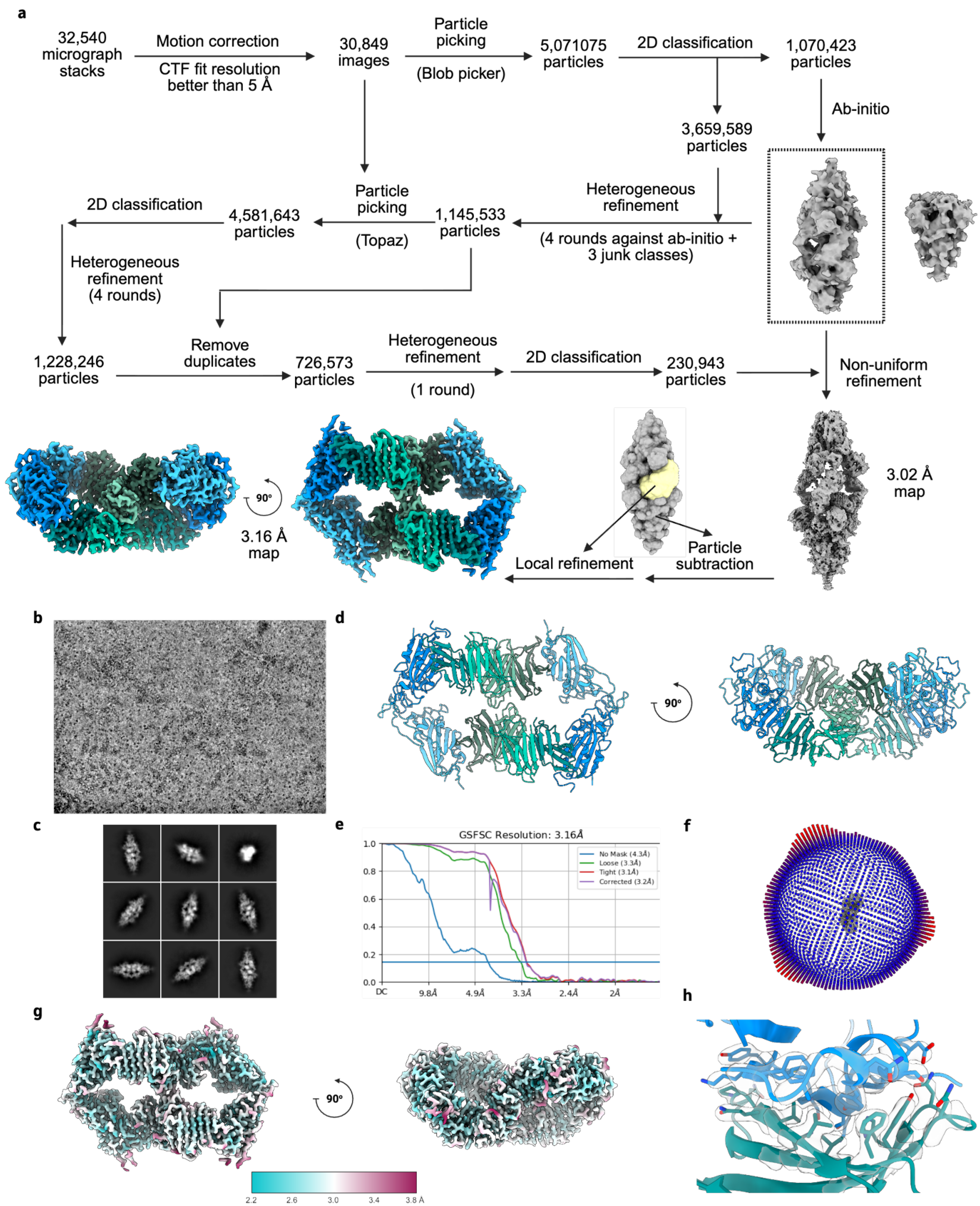

Supplementary Figure S20. Cryo-EM workflow for structure determination of the s19382-RBD (Omicron) complex. **a** Schematic of the cryo-EM data processing workflow for the s19382-Omicron Spike sample. RBDs in

the final reconstruction are colored in shades of blue and s19382 protomers are colored in shades of green. **b** Representative lowpass-filtered cryo-EM micrograph. **c** Representative 2D class averages. **d** Cartoon representation of the cryo-EM structure of the s19382-RBD (Omicron) complex, colored as in **a**. **e** Global resolution estimate based on the Fourier Shell Correlation (FSC) between two half datasets. **f** Angular distribution plot of all particles that contributed to the final map. The height and color (from blue to red) of the cylinder bars is proportional to the number of particles in those views. **g** Final reconstruction colored by local resolution. **h** Close-up view of the s19382-RBD (Omicron) interface with EMReady sharpened electron density map shown as a transparent surface. Structure is colored as in **a**.

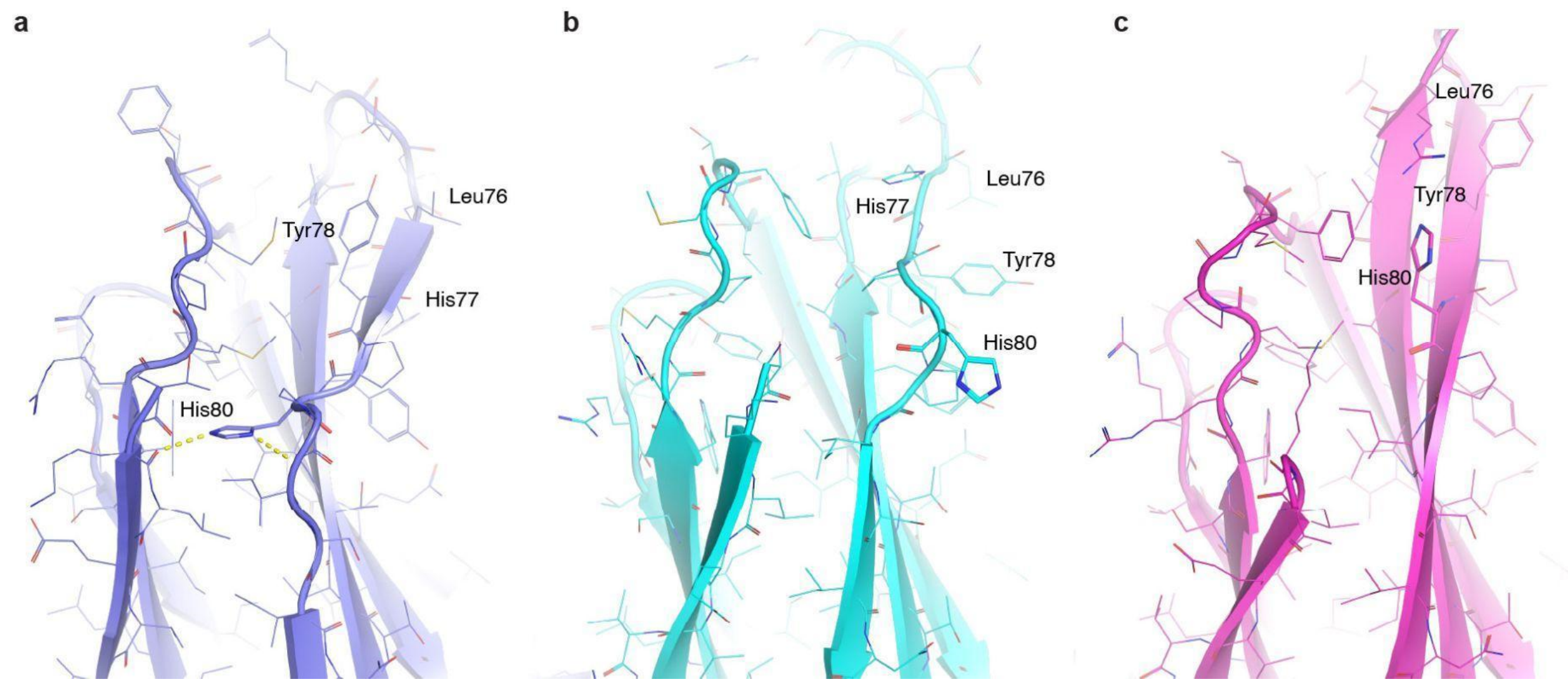

Supplementary Figure S21. His80 side chain packing in experimental, designed, and predicted s19382 models. **a** Cryo-EM structure of s19382, showing His80 contributes to packing between βA and βB. **b** Design model for s19382. His80 is solvent-facing, resulting in a βG register shift as compared to the experimental model. **c** AlphaFold3 prediction of s19382. His80 is more solvent-exposed than in the experimental model and does not contribute to packing of βA and βG.

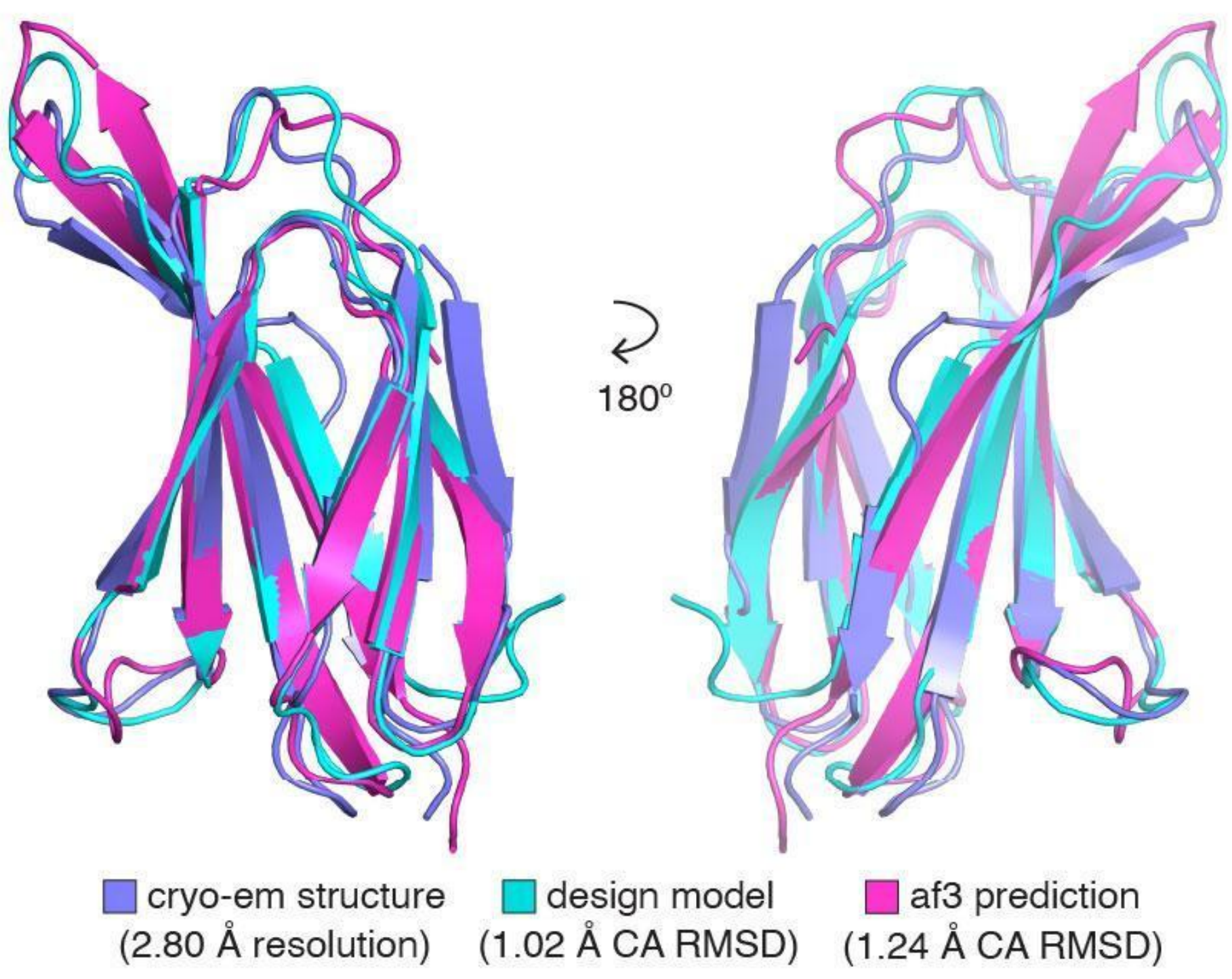

Supplementary Figure S22. Overlay of experimental, predicted, and designed structures of monobody s19382. Cryo-EM model of s19382 is shown in purple, design model is shown in cyan, and AlphaFold3 prediction is shown in magenta.

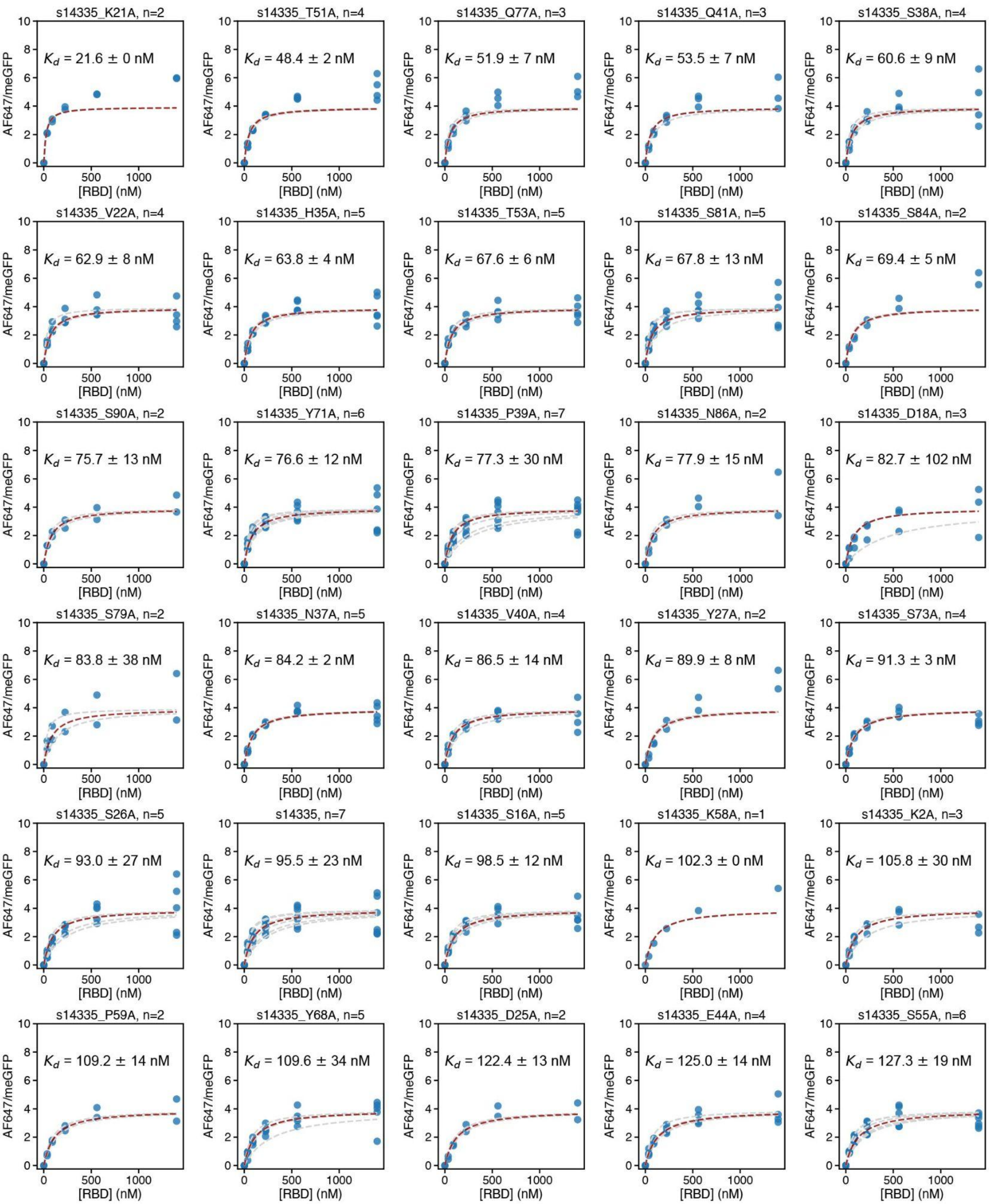
s14335_K21A, n=2
K_d = 21.6 ± 0 nM
s14335_T51A, n=4
K_d = 48.4 ± 2 nM
s14335_Q77A, n=3
K_d = 51.9 ± 7 nM
s14335_Q41A, n=3
K_d = 53.5 ± 7 nM
s14335_S38A, n=4
K_d = 60.6 ± 9 nM
s14335_V22A, n=4
K_d = 62.9 ± 8 nM
s14335_H35A, n=5
K_d = 63.8 ± 4 nM
s14335_T53A, n=5
K_d = 67.6 ± 6 nM
s14335_S81A, n=5
K_d = 67.8 ± 13 nM
s14335_S84A, n=2
K_d = 69.4 ± 5 nM
s14335_S90A, n=2
K_d = 75.7 ± 13 nM
s14335_Y71A, n=6
K_d = 76.6 ± 12 nM
s14335_P39A, n=7
K_d = 77.3 ± 30 nM
s14335_N86A, n=2
K_d = 77.9 ± 15 nM
s14335_D18A, n=3
K_d = 82.7 ± 102 nM
s14335_S79A, n=2
K_d = 83.8 ± 38 nM
s14335_N37A, n=5
K_d = 84.2 ± 2 nM
s14335_V40A, n=4
K_d = 86.5 ± 14 nM
s14335_Y27A, n=2
K_d = 89.9 ± 8 nM
s14335_S73A, n=4
K_d = 91.3 ± 3 nM
s14335_S26A, n=5
K_d = 93.0 ± 27 nM
s14335, n=7
K_d = 95.5 ± 23 nM
s14335_S16A, n=5
K_d = 98.5 ± 12 nM
s14335_K58A, n=1
K_d = 102.3 ± 0 nM
s14335_K2A, n=3
K_d = 105.8 ± 30 nM
s14335_P59A, n=2
K_d = 109.2 ± 14 nM
s14335_Y68A, n=5
K_d = 109.6 ± 34 nM
s14335_D25A, n=2
K_d = 122.4 ± 13 nM
s14335_E44A, n=4
K_d = 125.0 ± 14 nM
s14335_S55A, n=6
K_d = 127.3 ± 19 nM
AF647/meGFP
[RBD] (nM)

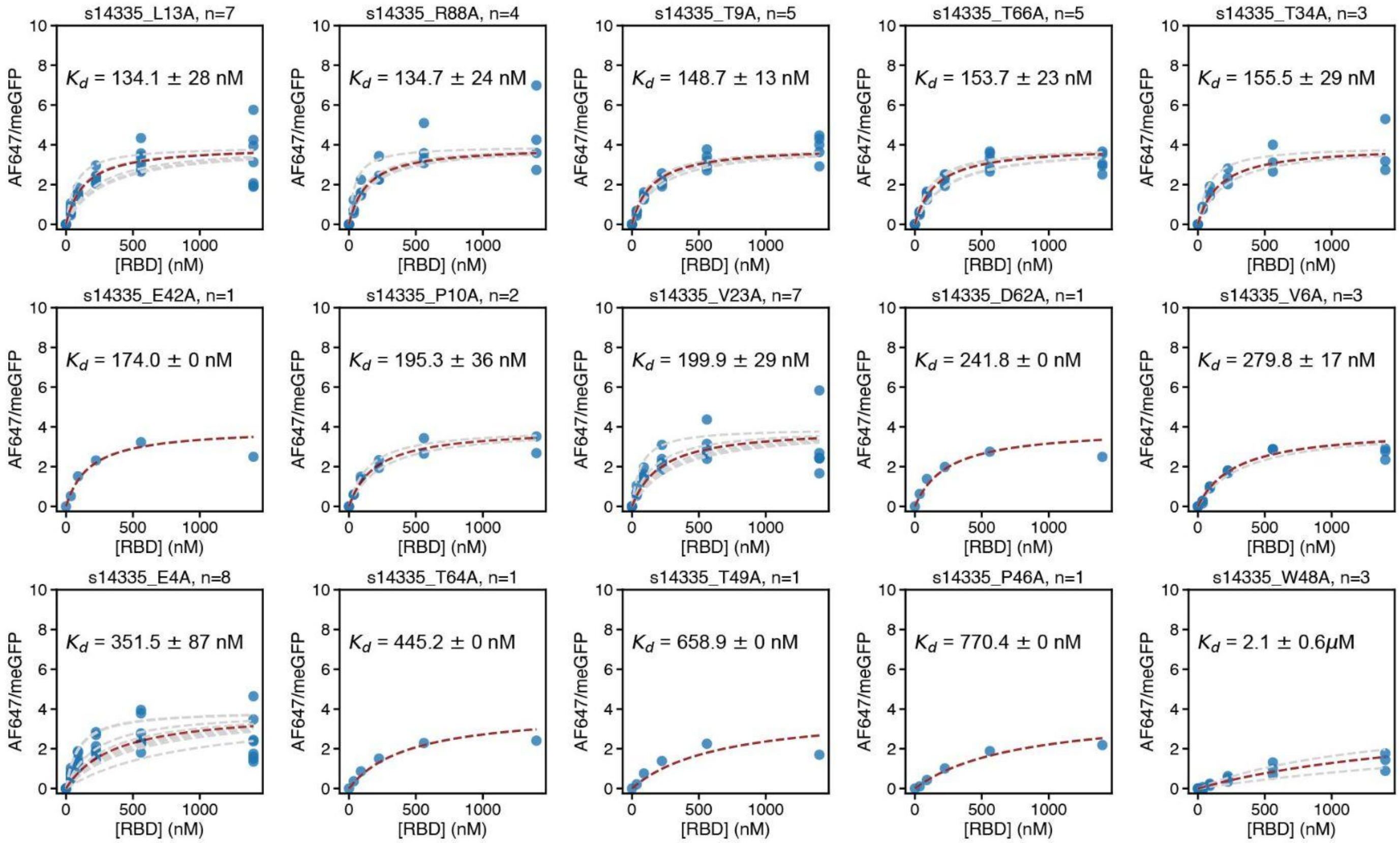

Supplementary Figure S23. Concentration-dependent binding curves for s14335 alanine variants. Light grey dashed lines indicate per-chamber Langmuir isotherm fits. The median K_d is annotated, and the solid red line indicates the fit returning this K_d. Blue markers indicate measured intensity ratios at each RBD concentration. Source data are provided as a Source Data file.

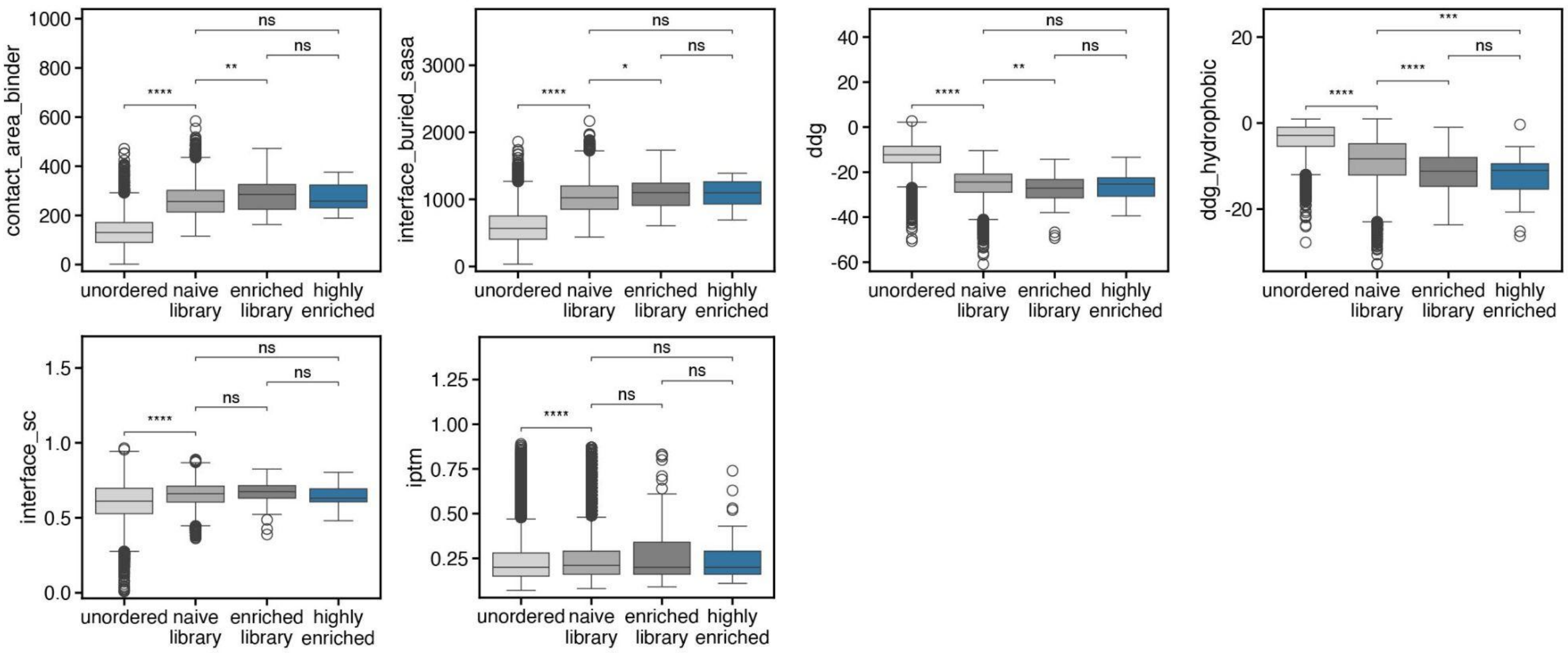

Supplementary Figure S24. Spread of scores for a range of computational metrics compared across the unordered (n=21,059), naive (n=10,867) enriched (n=92), and highly enriched (n=29) library subsets. Box plots show the median, interquartile range (25th–75th percentiles), and whiskers extending to 1.5× IQR. Two-sided t-tests were performed; ** denotes p<0.01, *** denotes p<0.001; **** denotes p<0.0001. Source data are provided as a Source Data file.

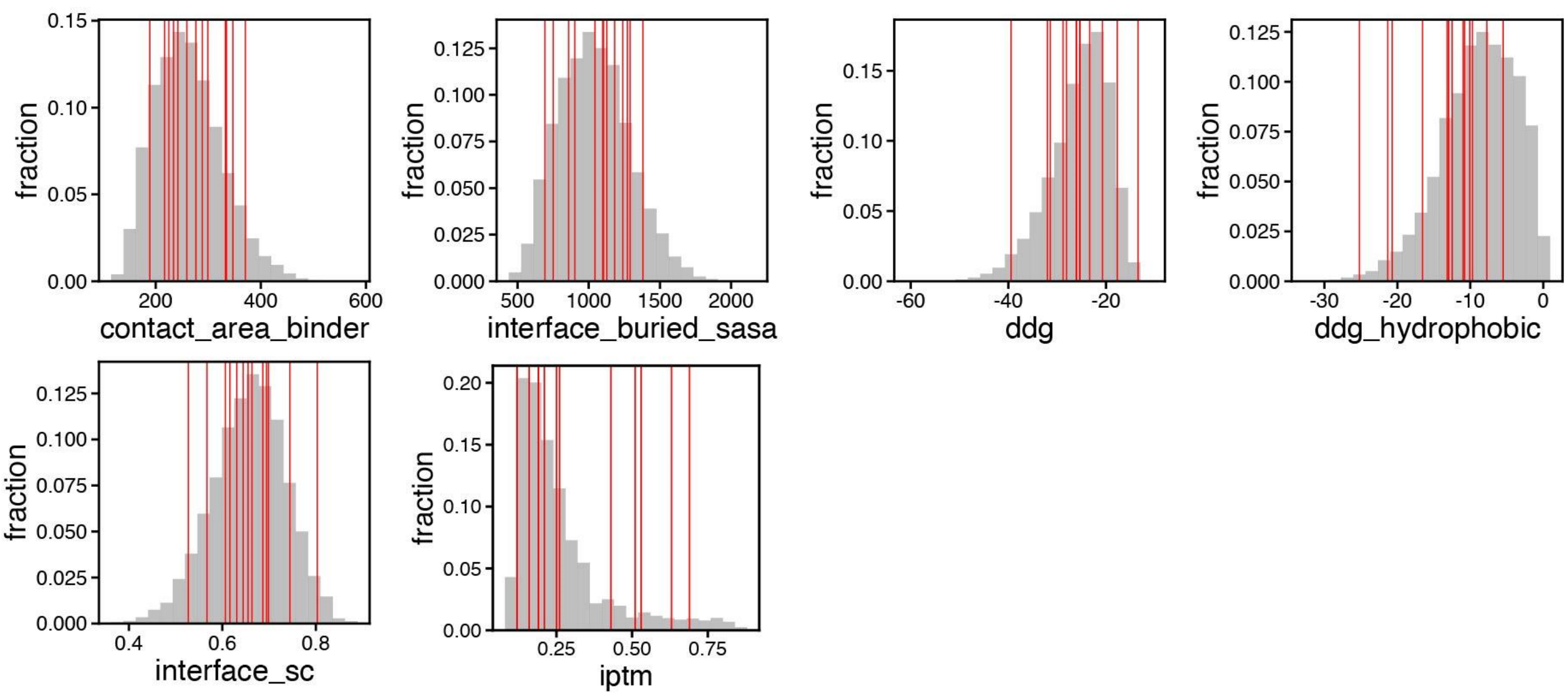

Supplementary Figure S25. Histogram demonstrating spread of computational metrics (Rosetta and AlphaFold3) across the designed library (grey, n=10,988 non-disulfide-stapled designs). Red vertical lines denote STAMMPPING validated binders. Source data are provided as a Source Data file.

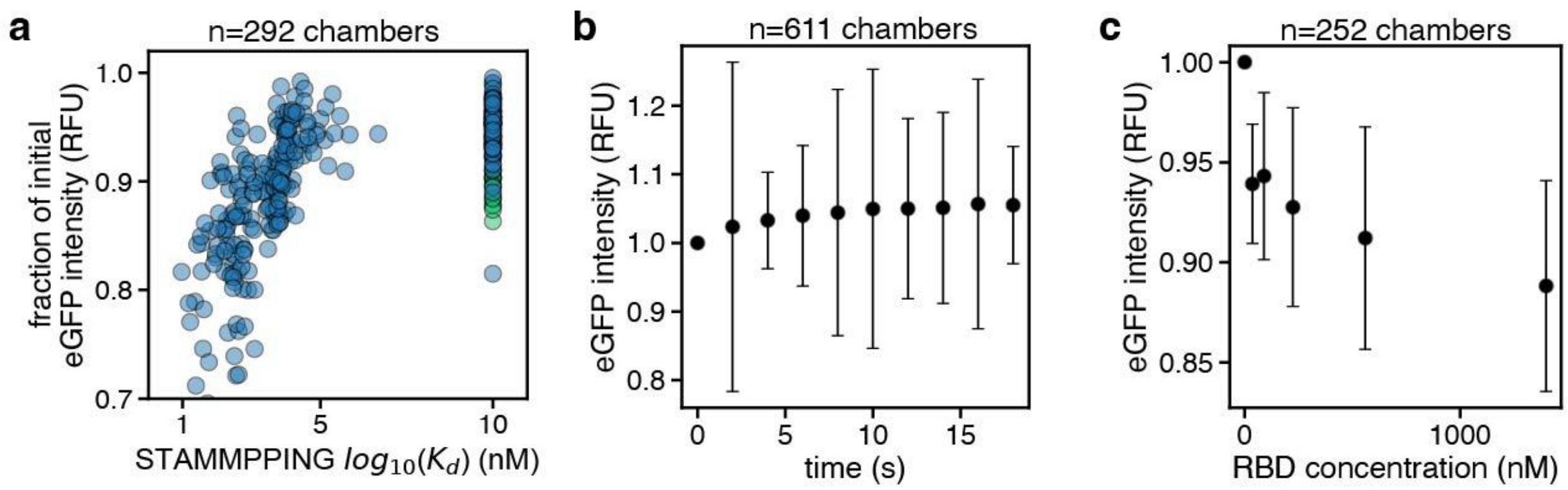

Supplementary Figure S26. meGFP quenching and recovery over the course of binding and kinetics measurements. **a.** Scatter plot of fraction of initial meGFP fluorescence intensity as a function of per chamber K_d measurements. Blue circles indicate monobody chambers, green circles indicate meGFP chambers. **b, c.** Black markers denote median intensity across all non-blank, non-culled sample chambers, and error bars denote standard deviation. **b.** Per chamber eGFP fluorescence as a function of time during dissociation kinetics measurements. **c.** Per chamber eGFP fluorescence as a function of RBD concentration during binding measurements. Source data are provided as a Source Data file.

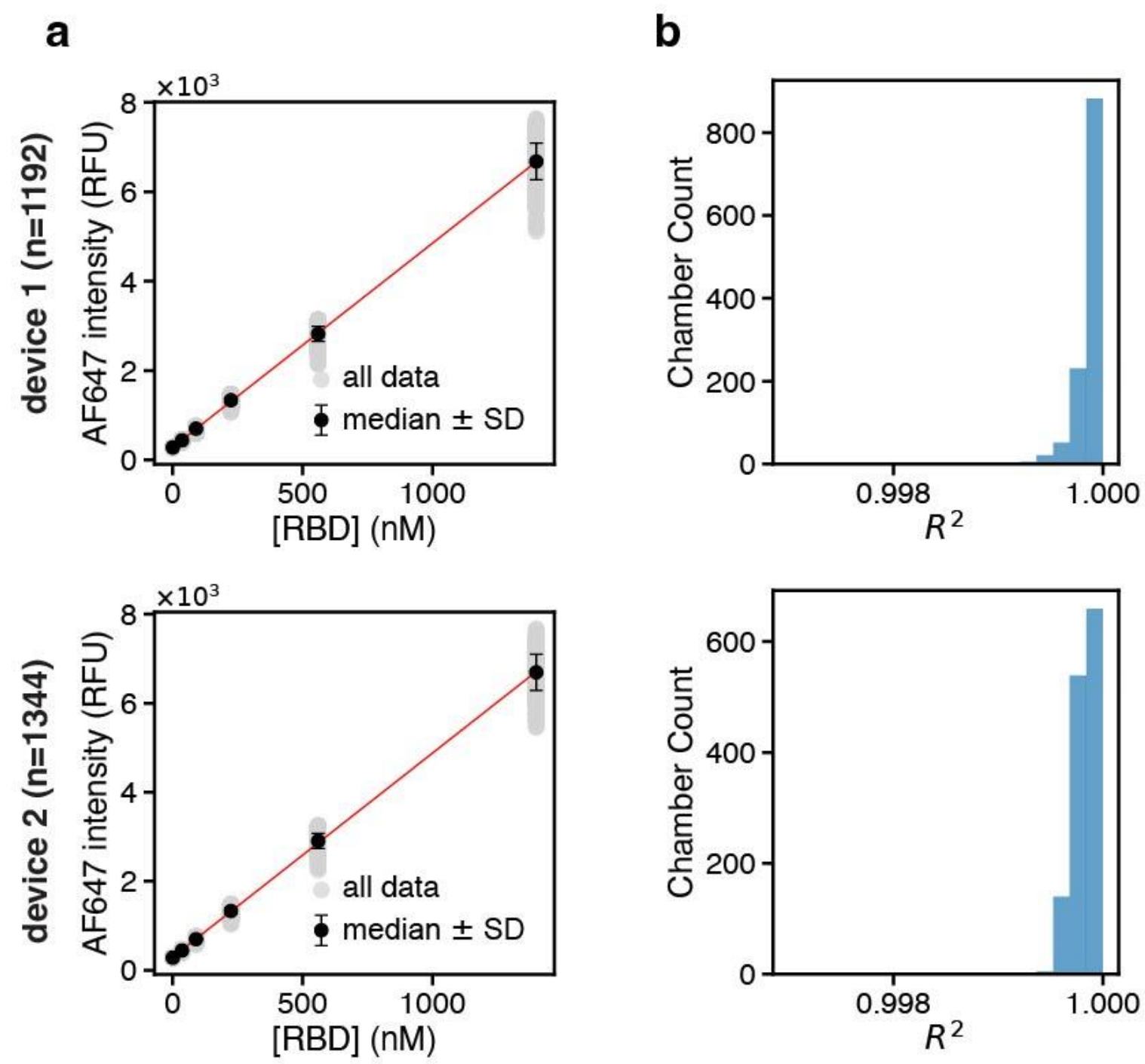

Supplementary Figure S27. Calibration curves relating AlexaFluor-647 fluorescence intensities to RBD concentration. **a.** Per chamber calibration curves across sample chambers used for analysis. Light grey markers denote median per chamber intensities following 500 ms exposure. Black markers denote median intensity across all chambers, and error bars denote standard deviation. Red line represents the best fit linear regression. **b.** Goodness of fit distributions for calibration curves. Source data are provided as a Source Data file.

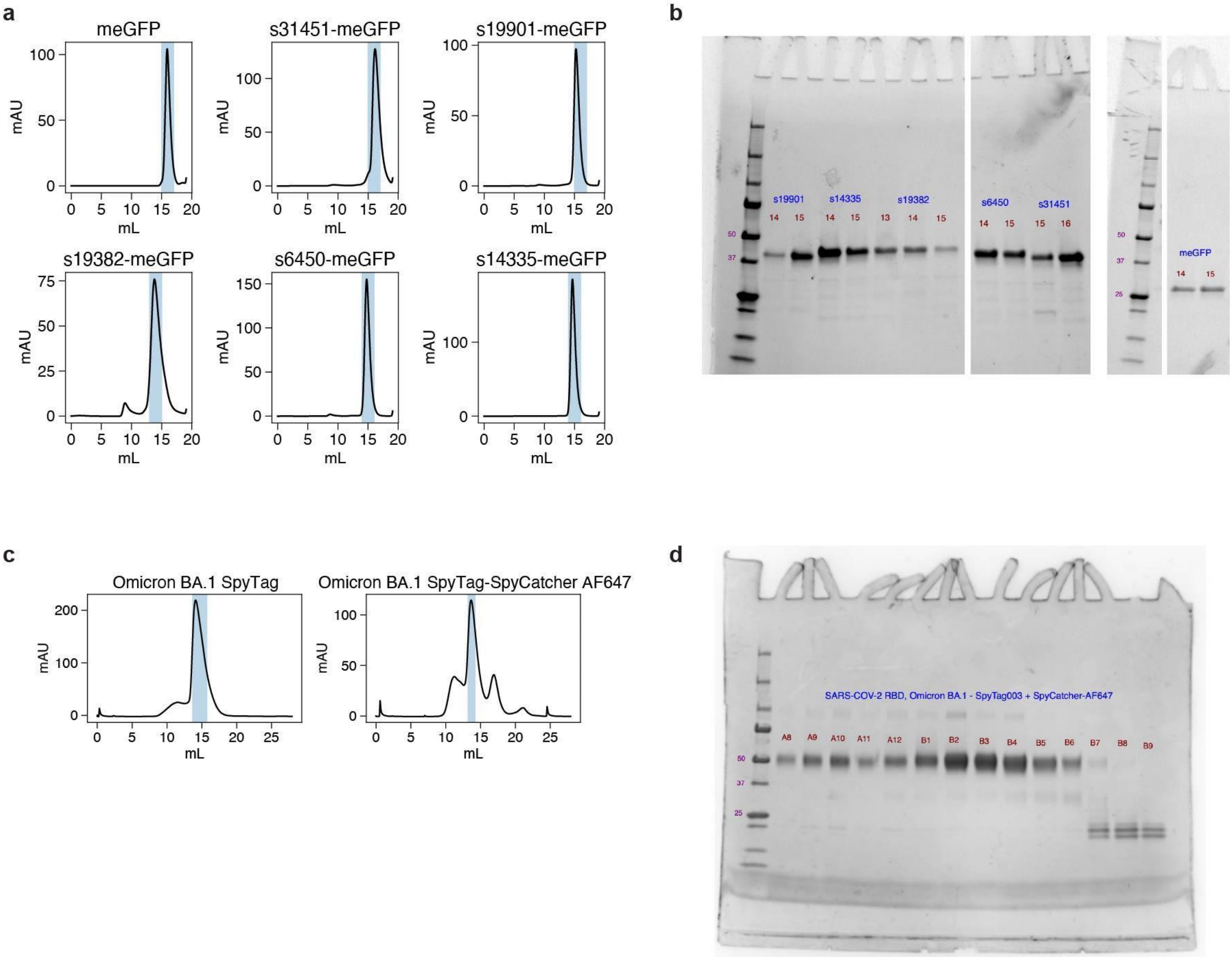

Supplementary Figure S28. SEC profiles (**a**, **c**) and corresponding SDS-PAGE gels (**b**, **d**) for SEC-purified monobodies and Omicron BA.1 RBD. Blue highlighted regions in SEC profiles correspond to fractions used for downstream experiments. SDS-PAGE was run under reducing conditions. For monobody-meGFP fusions, samples shown in SDS-PAGE gels were combined for each monobody and used for biolayer interferometry experiments. For Omicron BA.1 SpyTag-SpyCatcher AF647, samples B2 and B3 were pooled and used for STAMMPPING and YSD titration experiments. Source data are provided as a Source Data file.

Supplementary Table S1. Cost analysis for screening protein variants with either SPR or BLI versus our ADAPT-M + STAMMPPING strategy. Costs are based on screening 51 unique putative binders and exclude costs associated with replicate measurements, thereby representing an overestimate for ADAPT-M+STAMMPPING. CFPS costs were estimated using the commercially available PURExpress system. Source data are provided as a Source Data file.

Cost per screened variant	SPR / BLI	ADAPT-M + STAMMPPING
Gene synthesis	$34.79	$1.28
Antigen consumption (single cycle kinetics)	$21.98	$0.43
CFPS	$18.96	$1.24
Surface Patterning	NA	$0.25
Total cost	$75.73	$3.20

Supplementary Table S2. Comparison of traditional binding affinity methods (SPR, BLI) with the ADAPT-M + STAMMPPING workflow presented in this study.

	SPR / BLI	ADAPT-M + STAMMPPING
Cost per measurement	$75.73/variant	$3.20/variant
Gene synthesis	Individual gene fragments need to be ordered	No gene synthesis required, produced in-house by primers that can be shared across samples
Antigen Consumption	High antigen usage per reaction, even at 384-well plate scale	Low antigen quantity supports 1,000s of measurements (variants & replicates)
CFPS	Reactions can be scaled down to as low as 15uL[1] but limited by low-volume pipetting rather than protein yield	50uL reaction-scale sufficient for full microfluidic device (up to 1800 measurements) Not limited by low-volume pipetting constraints
Time to Measurement	10 days	5 days
Ordering Lead Time	8 day lead time required for sequencing, analysis, and gene fragment delivery before experiments can begin	No ordering required; measurements can begin immediately from existing YSD output
Measurement Throughput	8 samples at a time, but can be automated	~24 hours for ~1,200 measurements (including 600 blanks)
Hands-on Time	~4 hrs	~12 hrs
User Intervention	Primarily during plate setup; SPR/BLI protocols are more automated with minimal user intervention during runs. No hands-on time for DNA generation (ordered).	More user touchpoints during measurement, but largely automated. PCR steps required for DNA generation.
Multiplexing Capability	Yes, but more limited.	Yes
Multi-Antigen Testing	Multiple antigens possible, but must be done serially with surface regeneration between each antigen	Up to 8 antigens on a single device
Diversity Trade-off	Adding antigens proportionally increases required instrument	Additional antigens reduce per-antigen diversity, but parallel chip

	time	runs readily compensate
Binding Parameters	k_{on}, k_{off}, K_d	K_d
Kinetic Information	Full kinetic profiles (k_{on}, k_{off}, K_d) from a single experiment; gold standard for affinity characterization	steady state K_ds, apparent k_{off}

Supplementary Table S3: All monobody sequences recovered by ADAPT-M and measured by STAMMPPING. Source data are provided as a Source Data file.

monobody ID	protein sequence	# colonies picked
s8146	TKLEVVAATPTSLLIYWDHPMWDVDFYRITYGEVNGNSPVQSFNVPGS STVAWIYGLKPGVDYTITVYAYSGGSSQYFPSPISINYRTYNS	6
s19382	TKLEVVAATPTSLLISWRMPMFTVDFYVIQYGETGGNSPVQVQVVPGST RTATISGLKPGVDYTITVYAYVQRDNLHYPHPISINYRTGSK	23
s41795	TKLEVVAATPTSLLISWRHPMVYVDFYVITYGETHGNSPMQKFYVPGWK NTARISGLKPDVDYTITVYAYYSAPEWQKPSPISINYRTSGS	1
s22515	TKLEVVAATPTSLLISWHFPAVTVDFYVITYGETGGNSPTDVFIVPGSAS TATISGLYDFVDYTITVYAFVESGSKGAAKPISINMTGGGE	13
s6450	TKLEVVAATPTSLLISWDIPELQFDFYVITYGETFGNSPVQEFTVPGLATH ATISGLKPGVDYTITVYAYMSGSSYGSMIPISINYRTYGE	4
s5599	TKLEVVAATPTSLLISWDYPWYIFDFYRITYGEVGGNMPMQEYEVPGQI STATISGLKPGVDYTITVYAYVSIVEYYAPSPISINYRTRSG	2
s40215_2607	TKLEVVAATPTSLLISWDFPAVTIIFYFIEYGSTHGRDPIQSFRVPGSKST ATISGLKPGVDYTITVYAYHQRDNDHTPSPISINYRTGSG	1
s8891_s15323_ ds	TKLEVVAATPTSLLISWDAPAVWVFFYRITYGETGGNSPVQESFVPGDM STATISGLKPGVLYLITVYTMYHKNDEYPPNYHSPIFITYRT	3
s12658	TKLEVVAATPTSLLISWDAPAITVDWYTITYGETGGNMPMQSFFVPGSM STATISGLKRDVDYTITVYAQQITFEQYVPSPISINYRVGGE	15
s34551	TKLEVVAATPTSLLISWDHPAVDVNFYHITYGETGGNSPTYSFFVPGSV MTATISGLKPGVDYTITVYAHAASRYQAFPSPISINYRTGSP	7
s30122	TKLEVVAATPTSLLISWDFPAVTFDFYVITYGEVGGNSPVQEFVVPGSVS TATISGLKPYVDYTITVYAWYYRSDGGWGSPSSMKGHGSPG	3
s19901	TKLEVVAATPTSLLISWHWPAQTVDFYVITYGETGGNSPMQKFRVPGW VNTATISGLKPDVDYTITVYAHVVLPGHYSPPPISINYRPGPG	6
s7476_s38999	TKLEVVAATPTSLLISWDAPAVDFVYYVITYGETHGNSPVQEFRVPGRLS HATISGLKPGVDYTITVYAFYHSDDSYPAEFMHPISINYRT	2
s29883	TKLEVVAATPTSLLISWDMPMVTVVFYKITYGETGGNSPMQEFFVPGFL NTAKISGLKPGVDYTITVYAWLTGDEYYYSSPISINYRTESE	2
s1790	TKLEVVAATPTSLLISWDMPMVWVDFYVITYGEQGGNSPKWKFIVPGST STATISGLKPGVDYTITVYAYAYVDHSVKPIMWNPRFQSFGS	17
s37711	TKLEVVAATPTSLLISWDASQNHEYDFYRITYGQIGGVFPQYRFTVPGW TNTATITGLKPGVDYTITVYAYHKDGYLGNAFSSFNYRVGGG	11
s13677	TKLEVVAATPTSLLISWDAERNWVLFYIITYGETHGNKPVQSFRVPGWIS TAWITGLKPGVDYTITVYAAGSQDDGGAIWSPISINYRTGG	2

s32489	TKLEVVAATPTSLLISWHVPQSFVLFYVITYGETGGNSPVYHFFVPGSKN TATISGLKPGVDYTITVYAWQGGKDHFYGDSSGSYEGRPSS	2
s20904	TKLEVVAATPTSLLISWDAEAYTVDFYLITYGETGGNSPMQEFIVPGRVT TATISGLKPGVDYTITVYAFYYGHGEGSWSQSGGWGYYNEG	4
s2426_ds	TKLEVVAATPTSLLISWDAPMVTVDFYFIVYGEVGGTSPLQWFTVPGSK NTATISGLKPGVDYTICIYAFRTGSDWFIGSGHCSDGKGGNT	6
s30122_ds	TKLEVVAATPTSLLISWDFPAVTFDFYVITYGEVGGNSPVQEFVVPGSVS TATISGLKPYVDYTICVYAWYYRSDGGWGSPSCMKGHGSPG	8
s34551_s14252	TKLEVVAATPTSLLISWDHPAVDVNFYHITYGETGGNSPTYSFFVPGSV MTATISGLVPGVDYTITVYTWMSNYEDKSGSYYSPISINYRT	2
s31451	TKLEVVAATPTSLLISWDFPAVLVTFYLITYGETGMNSPMQEFWVPGSM STATISGLKPGVDYTITVYAWYYERDEYQLIESSPISINYRT	6
s15487	TKLEVVAATPTSLLISWDAPAVTVDFYLITYGEDGGNSPVQYFIVPGSVM TATISGLKPTVDYTITVYAYVVRSNFSFPSEISVYYSHGGG	4
s1137	TKLEVVAATPTSLLISWDQPMWTVDFYVITYGEQGRNQPMQKFIVPGSV STATISGLKPWVDYTITVQAFHFRNSRSQPSPISMYGSGGGG	6
s20460	TKLEVVAATPTSLLISWDQPNVDVVFYLITYGETGGNKPVQEHWVPGEK STATISGLKPGVDYTITVYTYVRDGDLYADKPPISINYRTEG	1
s14335	TKLEVVAATPTSLLISWDQSKVVVDSYVIAYGETHGNSPVQEFEVPGWT NTATISGLKPGVDYTITVYVFYVSAGDQMSGSPISINYRTSG	5
s22907	TKLEVVAATPTSLLISWDAPSSNVDFYLITYGETHGNHPVQEHVVPGSM STATISGLKPGVDYTITVYTMYWGGSNGGYSYGHPISINYRT	4
s38496	TKLEVVAATPTSLLISWDAPAVTVDYYVITYGERGGNSPVQEFIVPGSVR TATISGLKAGVDYTITVYAFVQHYDGHGSPYSSGHGSGGGS	6
s8891_s24482	TKLEVVAATPTSLLISWDAPAGWVFFYRITYGETGGNSPVQESFVPGDM STATISGLKPGVDYTIYVYAQYDGSGEVGGFSFEPHSQYYRG	3
s20225	TKLEVVAATPTSLLISWHAPNFYVDYYVITYGEHGYWPYYWQEDTVPGS MSTATISGLKPGVDYTITVYAGSNDDYNYYGSPISINYRTGG	1
s21007_s33549	TKLEVVAATPTSLLISWDHPWVDQDLYFIVYGVTGGNSPFQEFTVPGSF STATISGLKPGVDYLITVYAQYYRDGWMYSHPISIFYRTGES	3
s5087	TKLEVVAATPTSLLISWDMPALTVILYVITYGETGGNSPTQEFYVPGSKS TATISGLKPGVDYTITVYTVYEGDDETGYREDSPISINYRT	1
s7509	TKLEVVAATPTSLLISWDMPAVSVDFYVITYGEVGGVHDSGTYEVLGWF STATISGLRGMVDYTITVYAHMVYDDNNSPHPISGFGSGSGK	1
s37107	TKLEVVAATPTSLLISWMRPAVWVAFYVITYGEVGGNHPVQSFYVPGYV TTATISGLKPGVDYIITVYAWGTYSDEGFGSKSSGYGRAGGE	6
s22895	TKLEVVAATPTSLLISWDFPMVTVDLYHIIYGEVFGTSPIQEFTVPGSKST ATISGLKPGVDYQIVVYAMVSYRDWYYMSPIYINYRTGLS	4
s6699_ds	TKLEVVAATPTSLLISWDMPAVFVFFYLICYGEIGGNSPMQCFRVPGSKS TATISGLKPGVDYVITVYTWYYSDSDGGATYSSPIIILYQT	6

s27652	TKLEVVAATPTSLLISWWHPAVTVDFYVITYGETGGNSPTYRFIVPGSKN TATISGLSSDVDYTITVYAYMYGDGNKYPSPISINSGSGGG	1
s34966_s22269	TKLEVVAATPTSLLIEWSTPMVTVDFYHITYGETGGNSPMQEFTVPGFV TRATISGLQPGVDYTITVYAYVSYIEYQYESPISINYRPGDG	1
s28784_s25968	TKLEVVAATPTSLLISWDAPAVFVDFYLITYGETLGNSPVQEFFVPGSWS TATIEGLKPGVDYTITVYTWYYYDYHGLLYIYSPISINYRT	1
s1338	TKLEVVAATPTSLLISWHFPMQTVDFYLIEYGETGGNSPVQVFTVPGST STATISGLKPGVDYTITVYAYYVEIVFYFPSPISINYRTGLK	4
s38112_ds	TKLEVVAATPTSLLISWDAPAVTVEFYLITYGEQGGNSPWQSFLVPGSV STATISGLKPGVDYTICVEALVARDGNHARWSGGCGSGGGGK	1
s36609_s25706	TKLEVVAATPTSLLISWRHPAVTVQFYLITYGETGGNSPIQFHFVPGSKS TATISGLKPGVDYTITVYAYVYKYYPMFPSPISINYRTGPG	1
s37660	TKLEVVAATPTSLLISWDHPWVTVVYYVITYGETGGNSPVQEFLVPGSV NTAEITGLKPGVDYTITVYATYYSSKKYEYVYSHPISINYRT	1
s31740_s38496	TKLEVVAATPTSLLISWDKPAQTVDFYVITYGERGGNSPVQEFIVPGSVR TATISGLKAGVDYTITVYAFVQHYDGHGSPYSSGHGSGGGS	1
s38285	TKLEVVAATPTSLLISWDAPAVKVDYYVITYGEVGYWPYYWQEFTVPGS LSTAEISGLKPGVDYTITVYAGSYDQWYYWGKPISINYRTGG	1
s29734	TKLEVVAATPTSLLISWDHPAVDVDLYHITYGETFGNSPMQSDTVPGSS RTATISGLKPGVDYTITVYAYYSSSDYYQPSPISINYRTGMK	1
s24419	TKLEVVAATPTSLLISWDFPMVDVDFYRITYGETHENSPFQRDVVPGEK STATISGLKPGVDYTITVYAWYYGDSEKRIDTSHPISINYRT	1
s4171_s24471	TKLEVVAATPTSLLISWDAPAVWVRFYEILYGETGGDSPYYWFTVPGSK STATISGLKPGVDYVITVYATYYASTRGWYRRVVPVVIDYRT	1
s36171_s28705	TKLEVVAATPTSLLISWDAPALTVIFYVITYGETMGDSPLQQFVVPGSKS TATISGLKDGVDYLIIVYAYLHYSDDGYPPIISGGGGGGDG	1
s13507	TKLEVVAATPTSLLISWDFPNDTYDFYFIQYGWGWFGNGPHQEFTVPG SMNTATISGLKPGVDYTITVYAYYGSGGFSPDPVQINYRTGGT	1

Supplementary Table S4: Cryo-EM data collection, refinement and validation statistics

	#1 RBD-s19382<br>(EMDB EMD-72524)<br>(PDB 9Y5Y)
**Data collection and processing**	
Magnification	105,000
Voltage (kV)	300
Electron exposure (e–/Å^2)	42.5
Defocus range (μm)	0.8-2.8
Pixel size (Å)	0.84
Symmetry imposed	C1
Initial particle images (no.)	5,071,075
Final particle images (no.)	230,942
Map resolution (Å)	3.16
FSC threshold	0.143
Map resolution range (Å)	2.0-41.0
**Refinement**	
Initial model used (PDB code)	7U0N
Model resolution (Å)	3.31
FSC threshold	0.5
Model resolution range (Å)	2.0-41.0
Map sharpening *B* factor (Å^2)	-80.6
Model composition	
Non-hydrogen atoms	11,837
Protein residues	1492
Ligands	0
B factors (Å^2)	
Protein	64.80
Ligand	n/a
R.m.s. deviations	
Bond lengths (Å)	0.004
Bond angles (°)	0.668
Validation	
MolProbity score	1.00
Clashscore	0.94
Poor rotamers (%)	0.00
Ramachandran plot	
Favored (%)	96.59
Allowed (%)	3.27
Disallowed (%)	0.14

Supplementary Table S5: RBD interface residues identified by design, mutational mapping, and cryo-EM models for s19382-RBD. Residues validated by cryo-EM are marked in blue **bold** text.

Design model	R408, T415, G416, N417, D420, Y421, K424, L455, F456, R457, K458, S459, N460, Y473, Q474, A475, G476, N477
Mutational mapping	A344, A352, N354, **D364**, **N370**, P373, **F377**, G381, **P384**, **T385**, K386, D389, G413, Q414, R466, S469, P499
Cryo-EM	A363, D364, Y365, S366, Y369, N370, K376, F377, C379, S383, P384, T385

Supplementary Table S6: RBD interface residues identified by design model and mutational mapping for s14335-RBD.

Design model	R403, D405, R408, Q409, T415, G416, N417, Y421, L455, F456, R457, Y473, A475, F486, N487, Y489, H505
Mutational mapping	A344, Y351, N354, R355, K356, D364, S366, N370, L371, A372, P373, F374, F375, T376, F377, K378, G381, R403, Q414, G416, N437, P463, F464, E465, R466, D467, P499

Uncropped gel images.

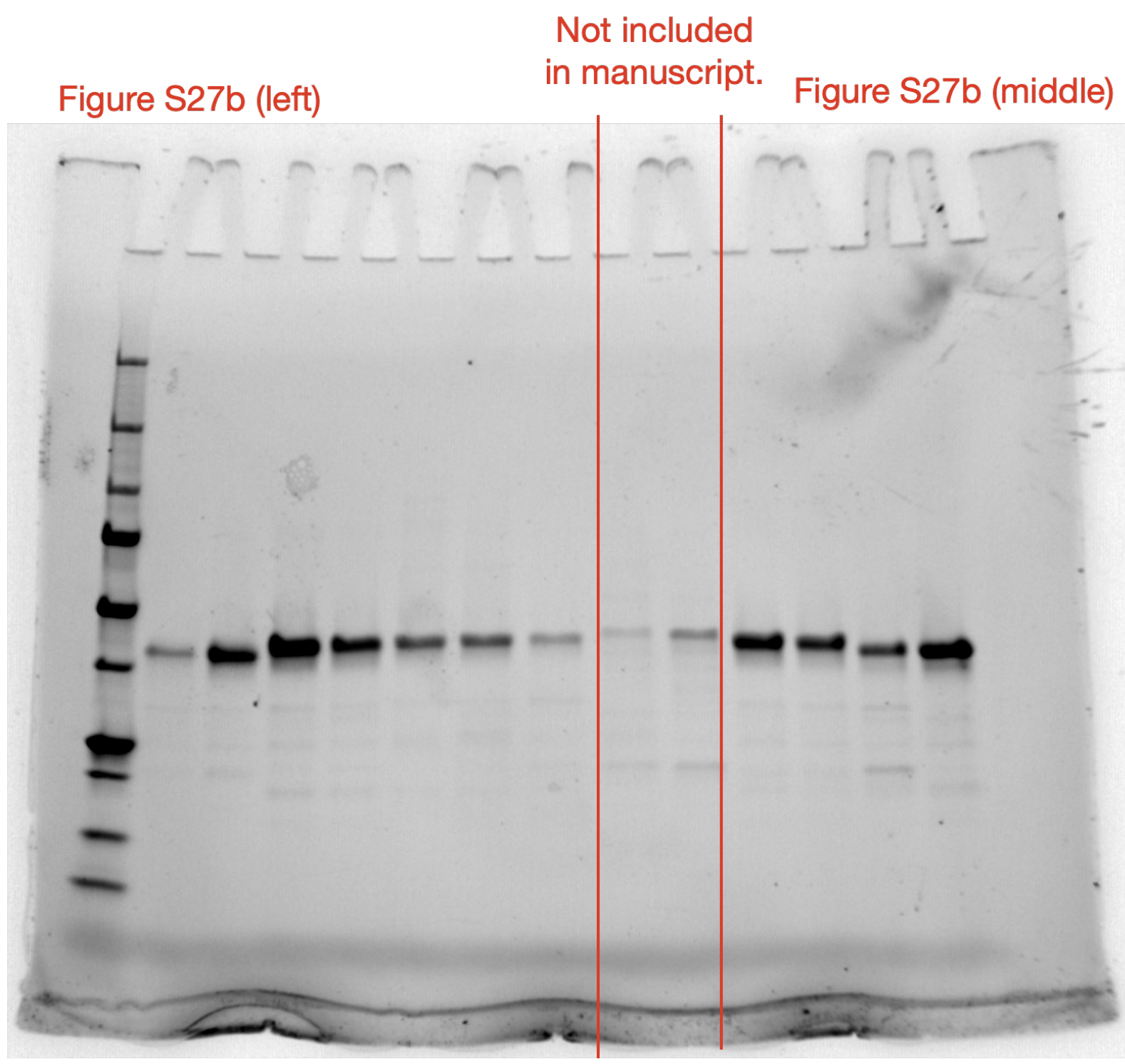

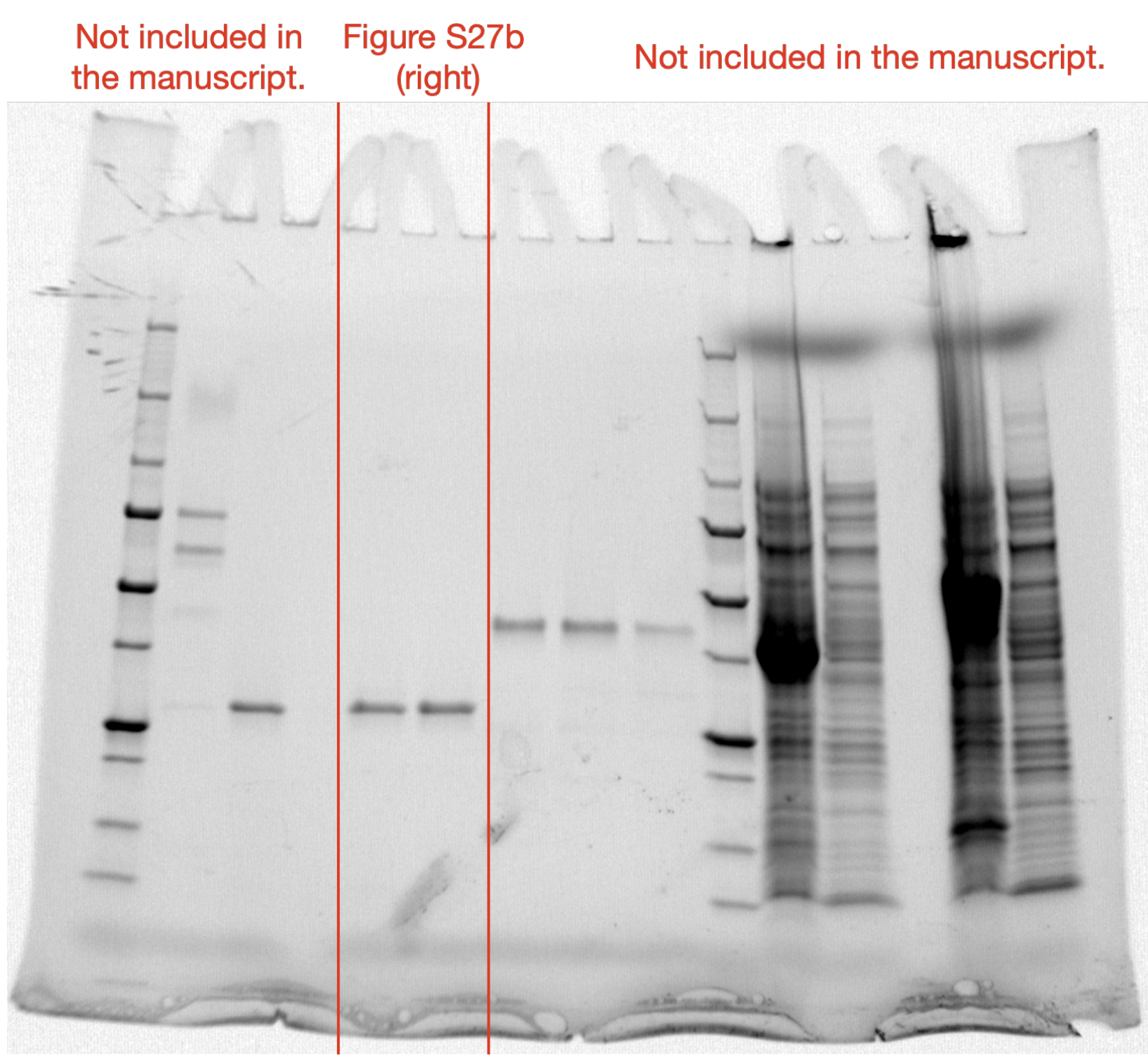

Figure S27d

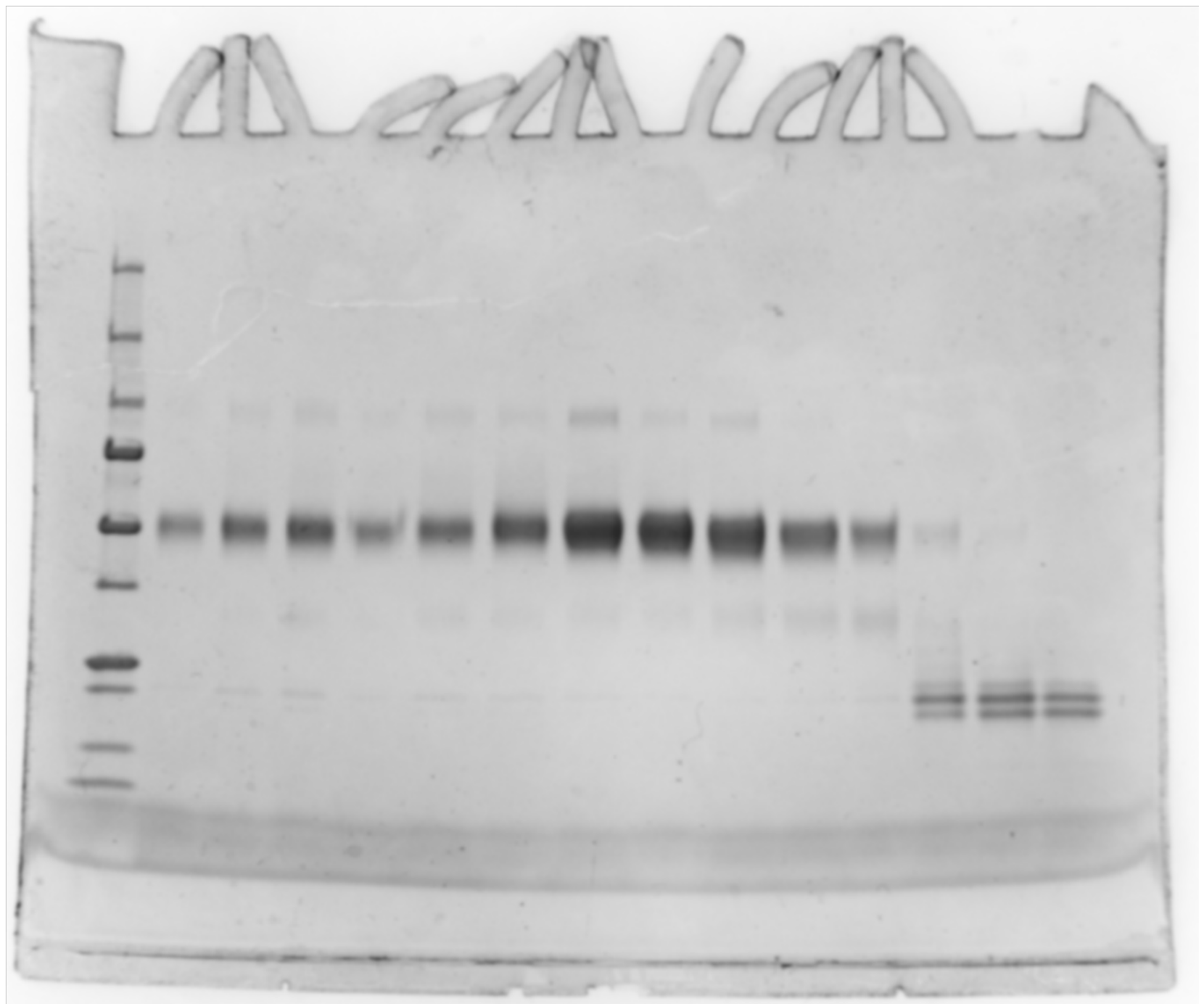

References

1. Warfel, K. F. *et al.* Cell-free expression and characterization of multivalent rhamnose-binding lectins using bio-layer interferometry. *Glycobiology* **33**, 358–363 (2023).
