# Supplementary material for: ADAPT-M: a workflow for rapid, quantitative in vitro measurements of enriched protein libraries
Source: Nat Commun. 2026 Jul 15;17:8673. doi: 10.1038/s41467-026-75463-1 (PMC13490508; doi:10.1038/s41467-026-75463-1)
Supplement: Supplementary file 2 [file 41467_2026_75463_MOESM2_ESM.pdf]

nature portfolio

Corresponding author(s): Possu Huang, Polly Fordyce, Nicole DelRosso

Last updated by author(s): May 31, 2026

Reporting Summary

Nature Portfolio wishes to improve the reproducibility of the work that we publish. This form provides structure for consistency and transparency in reporting. For further information on Nature Portfolio policies, see our Editorial Policies and the Editorial Policy Checklist.

Statistics

For all statistical analyses, confirm that the following items are present in the figure legend, table legend, main text, or Methods section.

n/a	Confirmed	
☐	☒	The exact sample size (*n*) for each experimental group/condition, given as a discrete number and unit of measurement
☐	☒	A statement on whether measurements were taken from distinct samples or whether the same sample was measured repeatedly
☐	☒	The statistical test(s) used AND whether they are one- or two-sided<br>*Only common tests should be described solely by name; describe more complex techniques in the Methods section.*
☒	☐	A description of all covariates tested
☐	☒	A description of any assumptions or corrections, such as tests of normality and adjustment for multiple comparisons
☐	☒	A full description of the statistical parameters including central tendency (e.g. means) or other basic estimates (e.g. regression coefficient) AND variation (e.g. standard deviation) or associated estimates of uncertainty (e.g. confidence intervals)
☐	☒	For null hypothesis testing, the test statistic (e.g. *F*, *t*, *r*) with confidence intervals, effect sizes, degrees of freedom and *P* value noted<br>*Give P values as exact values whenever suitable.*
☒	☐	For Bayesian analysis, information on the choice of priors and Markov chain Monte Carlo settings
☒	☐	For hierarchical and complex designs, identification of the appropriate level for tests and full reporting of outcomes
☐	☒	Estimates of effect sizes (e.g. Cohen's *d*, Pearson's *r*), indicating how they were calculated

Our web collection on statistics for biologists contains articles on many of the points above.

Software and code

Policy information about availability of computer code

Data collection	Automated control of pneumatic valves and microscopy was run through custom Python scripts (RunPack, AcqPack) as described previously (Markin et al. 2021).<br>Flow cytometry data were collected using Sony SH800 software.
Data analysis	STAMMPPING experiments were processed by ImageStitcher, an in-house publicly-available software package (https://github.com/FordyceLab/ImageStitcher) and ProcessingPack, an in-house publicly-available software package (https://github.com/FordyceLab/<br>All flow cytometry data were analyzed using FlowJo 10.8.1.<br>Bowtie version 2.5.4<br>BBMerge V39.01<br>Cutadapt V2.6<br>All statistical analyses and graphical displays were performed in Python version 3.12.9.<br>BLI data were analyzed using ForteBio Data Analysis 9.0. Structure visualization: PyMOL v2.5.2.

For manuscripts utilizing custom algorithms or software that are central to the research but not yet described in published literature, software must be made available to editors and reviewers. We strongly encourage code deposition in a community repository (e.g. GitHub). See the Nature Portfolio guidelines for submitting code & software for further information.

Data

Policy information about availability of data

All manuscripts must include a data availability statement. This statement should provide the following information, where applicable:

- Accession codes, unique identifiers, or web links for publicly available datasets
- A description of any restrictions on data availability
- For clinical datasets or third party data, please ensure that the statement adheres to our policy

Designs targeting the Omicron BA.1 RBD were modeled using the structure deposited under PDB accession code 7TN044 (https://doi.org/10.2210/pdb7TN0/pdb). Relaxed models of this structure in complex with the designed monobodies were used throughout figures in this manuscript. The solved cryo-EM structure for s19382 bound to Omicron BA.1 RBD was deposited to the PDB under accession code 9Y5Y (https://doi.org/10.2210/pdb9Y5Y/pdb) and the density map was deposited to the EMDB under accession code EMD-72524 (https://www.ebi.ac.uk/emdb/EMD-72524). pNVD006, the IVTT expression plasmid with C-terminal meGFP-tag used for expression with STAMMPPING, has been deposited on Addgene (Addgene ID: 229642, https://www.addgene.org/229642/). NGS data are available on the sequencing read archive under SRA accession code PRJNA1472999 (https://www.ncbi.nlm.nih.gov/bioproject/1472999). Processed affinity and kinetic measurements used in main text Figures are reproduced as Source Data; all other data (including quantified imaging data, fitted affinity and dissociation measurements, rosetta and AlphaFold3 metrics data) used to generate Supplementary Figures, Tables, and analyses are deposited as an Open Science Framework under accession code 7a8qg (https://osf.io/7a8qg/). Microfluidic wafer CAD design files, wafer fabrication protocols, and PDMS device fabrication protocols are freely available on the Fordyce lab website (www.fordycelab.com/microfluidic-design-files). CAD design files are also available on the Open Science Framework. Due to the large size of the imaging data, raw microscopy images are available upon request. Source Data are provided with this paper.

Research involving human participants, their data, or biological material

Policy information about studies with human participants or human data. See also policy information about sex, gender (identity/presentation), and sexual orientation and race, ethnicity and racism.

Reporting on sex and gender	*Use the terms sex (biological attribute) and gender (shaped by social and cultural circumstances) carefully in order to avoid confusing both terms. Indicate if findings apply to only one sex or gender; describe whether sex and gender were considered in study design; whether sex and/or gender was determined based on self-reporting or assigned and methods used.*<br>*Provide in the source data disaggregated sex and gender data, where this information has been collected, and if consent has been obtained for sharing of individual-level data; provide overall numbers in this Reporting Summary. Please state if this information has not been collected.*<br>*Report sex- and gender-based analyses where performed, justify reasons for lack of sex- and gender-based analysis.*
Reporting on race, ethnicity, or other socially relevant groupings	*Please specify the socially constructed or socially relevant categorization variable(s) used in your manuscript and explain why they were used. Please note that such variables should not be used as proxies for other socially constructed/relevant variables (for example, race or ethnicity should not be used as a proxy for socioeconomic status).*<br>*Provide clear definitions of the relevant terms used, how they were provided (by the participants/respondents, the researchers, or third parties), and the method(s) used to classify people into the different categories (e.g. self-report, census or administrative data, social media data, etc.)*<br>*Please provide details about how you controlled for confounding variables in your analyses.*
Population characteristics	*Describe the covariate-relevant population characteristics of the human research participants (e.g. age, genotypic information, past and current diagnosis and treatment categories). If you filled out the behavioural & social sciences study design questions and have nothing to add here, write "See above."*
Recruitment	*Describe how participants were recruited. Outline any potential self-selection bias or other biases that may be present and how these are likely to impact results.*
Ethics oversight	*Identify the organization(s) that approved the study protocol.*

Note that full information on the approval of the study protocol must also be provided in the manuscript.

Field-specific reporting

Please select the one below that is the best fit for your research. If you are not sure, read the appropriate sections before making your selection.

- [x] Life sciences
- [ ] Behavioural & social sciences
- [ ] Ecological, evolutionary & environmental sciences

For a reference copy of the document with all sections, see nature.com/documents/nr-reporting-summary-flat.pdf

Life sciences study design

All studies must disclose on these points even when the disclosure is negative.

Sample size	Sample size was determined based on similar biochemical studies, and >=2 biological replicates were performed to sufficiently demonstrate data reproducibility.
Data exclusions	STAMMPPING data were first filtered on the basis of eGFP expression as has been previously done using similar microfluidic approaches (Hastings, R., Aditham, A. K., DelRosso, N., Suzuki, P. H. & Fordyce, P. M. Mutations to transcription factor MAX allosterically increase DNA

selectivity by altering folding and binding pathways. Nat. Commun. 16, 636 (2025))

Additionally, binding data fits for variants where cy3 fluorescence (binding signal) was not significantly higher than blank (control) chambers (Supplementary Figure 6) were excluded as has previously been does for similar microfluidic approaches (Hastings, R., Aditham, A. K., DelRosso, N., Suzuki, P. H. & Fordyce, P. M. Mutations to transcription factor MAX allosterically increase DNA selectivity by altering folding and binding pathways. Nat. Commun. 16, 636 (2025); Aditham, A. K., Markin, C. J., Mokhtari, D. A., DelRosso, N. & Fordyce, P. M. High-Throughput Affinity Measurements of Transcription Factor and DNA Mutations Reveal Affinity and Specificity Determinants. Cell Syst. 12, 112-127.e11 (2021)).

Binding data collected via STAMMPPING without at least two replicates were not reported.

Replication	Each chamber within a microfluidic device on a STAMMPPING experiment represents a unique instance of an expressed an purified protein and can therefore be considered biological replicates. All binding and dissociation curves measured by STAMMPPING reported here had >=2 replicates. Results are further consistent across microfluidic devices (Supplementary Figure 5). At least three biological replicates of single cell sorting experiments and single colony picking experiments (an essential step of the ADAPT-M protocol), each, were completed (Supplementary Figure 2). BLI analysis was performed once for each reported interaction. At a minimum, two biological replicates were performed for each YSD titration experiment. Two biological replicates were performed for the s14335 yeast display SSM library, and a single replicate for the s19382 yeast display SSM library (Figure 5). Deep sequencing of all yeast libraries was performed once per library/library replicate.
Randomization	For STAMMPPING measurements, each variant appears at multiple, randomized locations across the device to prevent spatial bias.
Blinding	Analysis was not blinded as spatial identity of variants arrayed across microfluidic devices must be preserved for downstream analyses. However, since the data generated in this work are quantifiable values such as binding affinities, the risk from not blinding is negligible.

Reporting for specific materials, systems and methods

We require information from authors about some types of materials, experimental systems and methods used in many studies. Here, indicate whether each material, system or method listed is relevant to your study. If you are not sure if a list item applies to your research, read the appropriate section before selecting a response.

Materials & experimental systems

n/a	Involved in the study
☐	☒ Antibodies
☒	☐ Eukaryotic cell lines
☒	☐ Palaeontology and archaeology
☒	☐ Animals and other organisms
☒	☐ Clinical data
☒	☐ Dual use research of concern
☒	☐ Plants

Methods

n/a	Involved in the study
☒	☐ ChIP-seq
☐	☒ Flow cytometry
☒	☐ MRI-based neuroimaging

Antibodies

Antibodies used	Anti-c-myc-FITC (1:100, mouse, Clone: SH1-26E7.1.3, Miltenyi Biotec, 130-116-485) was used for detecting monobody expression on yeast surface. anti-GFP nanobody (Proteintech, gtb-250) was used for purification in STAMMPPING experiments.
Validation	All antibodies used in this study have been validated by the respective vendor and have been used in published studies. Validation details can be found at the following locations:<br>130-116-485: https://www.miltenyibiotec.com/US-en/products/c-myc-antibody-anti-human-mouse-rat-sh1-26e7-1-3.html#conjugate=fitc:size=100-tests-in-200-ul<br>gtb-250: https://www.ptglab.com/products/GFP-VHH-biotinylated-recombinant-binding-protein-gtb.htm?srsltid=AfmBOooPHIqmtNmQ2rw8oeffeQFnXYlyuyf8kvjq7dyKJKwVUdhvQ7qe

Plants

Seed stocks	*Report on the source of all seed stocks or other plant material used. If applicable, state the seed stock centre and catalogue number. If plant specimens were collected from the field, describe the collection location, date and sampling procedures.*
Novel plant genotypes	*Describe the methods by which all novel plant genotypes were produced. This includes those generated by transgenic approaches, gene editing, chemical/radiation-based mutagenesis and hybridization. For transgenic lines, describe the transformation method, the number of independent lines analyzed and the generation upon which experiments were performed. For gene-edited lines, describe the editor used, the endogenous sequence targeted for editing, the targeting guide RNA sequence (if applicable) and how the editor was applied.*
Authentication	*Describe any authentication procedures for each seed stock used or novel genotype generated. Describe any experiments used to assess the effect of a mutation and, where applicable, how potential secondary effects (e.g. second site T-DNA insertions, mosiacism, off-target gene editing) were examined.*

Flow Cytometry

Plots

Confirm that:

- [x] The axis labels state the marker and fluorochrome used (e.g. CD4-FITC).
- [x] The axis scales are clearly visible. Include numbers along axes only for bottom left plot of group (a 'group' is an analysis of identical markers).
- [x] All plots are contour plots with outliers or pseudocolor plots.
- [x] A numerical value for number of cells or percentage (with statistics) is provided.

Methodology

Sample preparation	To prepare yeast cells for sorting: 1-2 x 10^7 induced yeast cells were washed three times with PBSA (PBS containing 0.5% BSA). Yeast cells were labeled with anti-c-myc fluorescein isothiocyanate (FITC, Miltenyi Biotec) (1:100) and RBD for 60 minutes at room temperature on a rotating platform. Yeast cells were then washed once, resuspended in PBSA, and labeled with Streptavidin, R-Phycoerythrin (SAPE, Invitrogen) (1:100) for 10-15 minutes at room temperature on a rotating platform. Following secondary labeling, yeast cells were washed once in PBSA and kept on ice as pellets. Just before sorting, yeast cells were resuspended in PBSA and passed through a 35µm mesh filter.<br><br>To prepare bacteria for sorting: After cultures reached the target OD, 5 x 10^7 cells (assuming 1 x 10^8 cells/mL at OD=1) were washed once in PBSA (PBS supplemented with 0.5% BSA). Cells were resuspended in a 1mL volume and labeled with 5 µL of 1 mg/mL Propidium Iodide (PI) and incubated for 5 minutes on a tube rotator, protected from light. Following incubation, cells were washed once in PBSA and resuspended to a final cell concentration of 0.25 x 1^06 cells/mL before being passed through a 35 µm mesh filter.
Instrument	Sony SH800
Software	Raw data were collected from instrument supplied software. Data were analyzed using FlowJo X (10.9) and FlowJo 11 (11.1.1).
Cell population abundance	Cell populations are gradually enriched after each round of sorting. Abundance is measured by presence of double positive binding and expression signal. Using yeast surface display as an engineering step does not require population analysis. We sequence sorted populations by NGS.
Gating strategy	For yeast: after gating for singlets, cells were gated for positive binding (either PE or AF647 labeled antigen) and positive expression (FITC labelled myc).<br><br>For bacteria: after gating for singlets, cells without Propidium Iodide signal were gated.

- [x] Tick this box to confirm that a figure exemplifying the gating strategy is provided in the Supplementary Information.
