# Supplementary material for: ADAPT-M: a workflow for rapid, quantitative in vitro measurements of enriched protein libraries
Source: Nat Commun. 2026 Jul 15;17:8673. doi: 10.1038/s41467-026-75463-1 (PMC13490508; doi:10.1038/s41467-026-75463-1)
Supplement: Supplementary file 3 [file 41467_2026_75463_MOESM3_ESM.pdf]

nature portfolio

Peer Review File

ADAPT-M: A workflow for rapid, quantitative in vitro measurements of enriched protein libraries

Corresponding Author: Dr Po-Ssu Huang

This file contains all reviewer reports in order by version, followed by all author rebuttals in order by version.

Version 0:

Reviewer comments:

Reviewer #1

(Remarks to the Author)
I co-reviewed this manuscript with one of the reviewers who provided the listed reports. This is part of the Nature Communications initiative to facilitate training in peer review and to provide appropriate recognition for Early Career Researchers who co-review manuscripts.

Reviewer #2

(Remarks to the Author)
Summary:
The authors present ADAPT-M (Affinity Determination by Adaptation of ProTein binders for Microfluidics), which combines an initial screening using yeast surface display (YSD) with quantitative evaluation of selected binders using the STAMMPPING microfluidic platform. The authors demonstrate the ability to measure affinity and dissociation kinetics for hundreds of enriched protein variants in a matter of days, without the need for traditional protein purification
This development is timely; with the increasingly rapid development of machine-learning methods for de novo binder generation, there is a growing demand for fast, high-throughput, and quantitative experimental validation methods. Several methods have been developed for this purpose, including TITE-seq, MAGMA-seq, and AlphaSeq, though these often remain labor-intensive or lack precise biophysical parameter measurement.
The authors optimize the method by selecting a monobody library against the Omicron SARS-CoV-2 RBD. They then validate the workflow by comparing in cellulo and in vitro affinities, and by characterizing two lead binders using cryo-EM and epitope/paratope mapping.

General Assessment:
Pérez et al. present ADAPT-M, a new method for fast and quantitative analysis of protein–protein interactions in enriched protein libraries. The approach combines the power of an initial yeast-display screening to enrich candidate binders with a second quantitative validation step using STAMPPING. The amount of work presented is substantial, and the experiments are carefully executed. Furthermore, the validation of the results using multiple orthogonal techniques convincingly demonstrates the robustness of the method for identifying protein binders. This combination of technologies is timely and valuable for the field, especially given the increasing demand for high-throughput binder discovery and kinetic characterization tools.
The writing is clear and the figures are of high quality. I recommend publication after the authors address a few minor points and clarify specific technical aspects. This will improve the article and it clearer and more accessible to readers.

Minor points
1. A new supplementary figure with information about the last purification step (SEC profile) and SDS-PAGE should be included in the paper to ensure reproducibility.
2. Data and text related to Figure 5. The authors perform epitope mapping of s19382 and s14335, using deep mutational scanning via yeast display. The results indicate that both selected monobodies for validation bind different epitopes than the ones used during the design and the ones predicted using AF3. Then, they use cryo-EM to solve the structure of RBD-bound monobody s19382, confirming these results. I think the authors make a good job with this validation workflow, but somehow the representation of the results is not good enough to give the reader a clear picture of the RBD epitope and how it compares to the residues identified by mutational analysis. I would suggest the authors to:

A. Include somewhere in the text, figures or supplementary figures, a list with the identified residues forming the RBD epitopes, and which amino acids forming this experimental epitope could be identified using mutational scanning.
B. Panel D should be improved to give a clear picture of the RBD epitope and the monobody paratope, or at least to show the major amino acids involved in binding. Below we show an idea on how this part of the figure could look (just a suggestion using ChimeraX, see attached), and labels should be added to the main amino acids at the interface to make it easier for the reader to follow it.

3. Figure S18 could be improved if each designed model again RBD would be represented in independent panels. As the two models are represented at the same time, there are chain clashes and could be confusing to the reader.
It would be clearer if its represented (A) s14335 binding to RBD and then (B) s19382 binding to RBD.
4. Figure S20 should be remade highlighting only (with a different color, for example) the amino acids that form the interface between the monobody and the RBD.
5. In figure S25, it mentions AlphaFold2 but probably it would be AlphaFold3?
6. More information about the sonication conditions could be given in the Materials and methods.
7. More detailed information about Ni-NTA and SEC purifications could be added to facilitate reproducibility.
8. The authors state that AlphaFold3 predictions were unsuccessful. The use of AlphaFold3, even with the standard parameters, should be described in the methods.
9. In Figure S19, a low pass filtered image of a micrograph could be shown to check particle distribution of the dataset as well as the projections from the 2D classes obtained in the 2D classification step.
10. In Figure S19, panel e) add the resolution units (Å) to the colored bar, the units (Å) can also be added in the figure legend.

Reviewer #3

(Remarks to the Author)
Review of NCOMMS-25-82922

“ADAPT-M: A workflow for rapid, quantitative in vitro measurements of enriched protein libraries” by Perez et al.

Summary:

Perez et al. introduce ADAPT-M, an integrated experimental workflow that connects yeast surface display-based enrichment with rapid microfluidic affinity measurements using their STAMMPPING platform. The authors demonstrate the approach by evolving monobodies against SARS-CoV-2 Omicron BA.1 RBD, assessing enriched variants without purification, validating selected hits with orthogonal biophysical assays, and presenting structural and mutational analyses of lead binders. The manuscript is clearly structured and technically well executed, and the demonstrated application is compelling. The following points should be addressed before publication:

1) While the authors repeatedly emphasize a “high-throughput” capability, the demonstrated quantitative characterization scale (33 variants after enrichment from an initial library of ~12,000) may not, in itself, constitute high throughput in terms of the measurement that is performed on the 33 variants. The throughput claims should be moderated and/or more clearly contextualized relative to existing, comparably scalable methods.

2) The discussion strongly highlights the limitations of alternative affinity-determination methods (ITC, BLI, etc.), yet provides comparatively little critical reflection on the limitations intrinsic to the ADAPT-M and STAMMPPING approach. Also, as a surface-based assay, effects such as surface heterogeneity, mass-transport limitations, avidity, and probe-density dependence should be acknowledged.

3) Broader adoption of ADAPT-M may be limited by platform accessibility, given the specialized microfluidic infrastructure and automation required. It would substantially support dissemination if the authors make microfluidic chip designs, operating parameters, and setup guidelines openly available and provide detailed instructions on how to setup and operate these measurements.

4) The workflow does not extract quantitative kinetic constants (kon/koff) and appears limited to equilibrium binding measurements plus qualitative dissociation behaviour. The authors should explain the origin of this limitation, whether it is fundamental to the current architecture, and whether temperature-dependent measurements (and thermodynamic analyses) are feasible.

5) The authors should clarify the dynamic range realistically accessible with this format, including upper and lower measurable affinity limits, and whether very strong binders (low-nM or pM) or weak binders can be accurately resolved.

6) Some false positives remain after YSD enrichment. It would be valuable to discuss why these persist mechanistically and whether adjustments in the enrichment workflow could mitigate them.

7) Because quantification relies on display-linked readout, there may be a bias toward variants with higher expression rather than strictly higher affinity. It is worth clarifying whether low-expressor high-affinity clones could be underrepresented, and whether the platform can experimentally disentangle expression level from true affinity.

8) Reproducibility across devices, days, and operators is not discussed or shown in depth. Presenting such assessment,

even briefly, would increase confidence in robustness.

Reviewer #4

(Remarks to the Author)
The manuscript by Perez et. al. presents a new methodlogy for high throughput screening of protein-protein interactions using yeast suface display and a microfluidic platform. The proposed pipeline has the advantage of being capable of determining Kds in unpurified samples. In this case the authors use a library of computationally designed binders against the SARS-CoV-2 Omicron BA.1 protein to validate the methodology, thoug it holds the potential to screen other binder molecular such as VHHs.
Subject to minor revisions focused on clarity, scalability arguments, and cost considerations, the manuscript is suitable for publication.
Introduction and motivation
- Lines 50–52: SPR and BLI indeed have lower throughput in their standard formats, but high-throughput variants exist (automated 96-well or 384-well arrays). The manuscript should acknowledge these developments and clearly articulate the comparative advantages of ADAPT-M beyond scaling (e.g., cost per measurement, hands-on time, multiplexing capability).
- Line 55: The term neutralization is too broad in this context. Please specify whether the authors refer to measuring competitive inhibition, ligand blocking, epitope occlusion, or another operational definition within the ADAPT-M workflow.
- Lines 70–71: Only 14 binders ultimately yielded measurable Kd values above the detection threshold. Although the platform itself is scalable, the results provided do not yet demonstrate high-throughput capability compared to cell-free synthesis coupled with SPR/BLI, where tens to hundreds of binders can be screened routinely. The authors should temper the claim or provide quantitative rationale for throughput scaling (e.g., number of chambers, device multiplexing, total measurement capacity).
- Line 88: Please specify the total number of days required for Stage 1. The text states 2–3 weeks for YSD enrichment, while line 93 indicates 3 weeks. A consolidated timeline per stage would improve clarity.
- Line 103: To strengthen the argument for high throughput, automated colony picking should be mentioned as a compatible option. Manual colony picking is not scalable and limits the practical utility of the pipeline.
- Lines 109–110: The authors should comment on the number of colonies picked versus unique sequences obtained, and whether diversity loss occurred at this step. This is relevant for assessing bottlenecks in the pipeline.
- Line 115: "Supplementary*".
- Lines 117–118: Because the pipeline relies on capture via an anti-eGFP nanobody and uses microfluidic devices with patterned surfaces, the authors should provide an approximate cost per screened variant and indicate whether patterned slides or devices can be reused. For adoption as an HT methodology, cost efficiency is critical.
- Line 132: Given the strongly methodological nature of the manuscript, please report the time required for oligo pool cloning and whether any QC step was performed to evaluate representation of the designed library in the YSD format. Recovery rate or dropout analysis would be useful.
Discussion
- The manuscript should more explicitly discuss the mismatch between in-cellulo and in-vitro affinities. The strong correlation between STAMMPPING and BLI contrasts with the weaker correspondence to YSD titrations. This supports a known bias in YSD where epitope accessibility, multivalent avidity, and cell surface context can inflate apparent affinity. This should be acknowledged and framed as motivation for the ADAPT-M workflow.
Figure 1
- Panel a: The STAMMPPING diagram is visually dense. Enlarging or simplifying it would improve readability and support broader adoption of the method.
- Panel b: The caption refers to "meGFP binding to the VHH," which is misleading. This step corresponds to immobilization or capture, not binding. Rephrase accordingly.
- Panel c: The methods imply expression in bacteria prior to STAMMPPING, not in a cell-free transcription–translation system. The backbone used should be clearly indicated to avoid confusion between IVTT and bacterial expression.
- Panel b–c: This is a picky detail so feel free to ignore. If the amplicon map is the same in both panels, the T7 promoter should be represented using the same color for consistency.
Additional points
- Clarify whether PDMS–glass devices, once patterned, can be washed and reused. This has practical impact on throughput, cost, and reproducibility
- The authors use meGFP-only and empty chambers, but nonspecific interactions appear variable. They should comment on how variability in background binding affects Kd estimation at the lower affinity limit.
- The global fitting approach presumes similar stoichiometry and surface presentation. The authors should briefly justify this assumption, especially since monobodies differ in expression levels and may exhibit different immobilization efficiencies.
- The compressed dynamic range observed when RBD is labeled via the His-Avi/avidin system deserves a brief mechanistic interpretation (e.g., avidity, probe geometry). A short cautionary note in the discussion would guide users applying ADAPT-M to different targets.

Version 1:

Reviewer comments:

Reviewer #1

(Remarks to the Author)
I co-reviewed this manuscript with one of the reviewers who provided the listed reports. This is part of the Nature Communications initiative to facilitate training in peer review and to provide appropriate recognition for Early Career Researchers who co-review manuscripts.

Reviewer #2

(Remarks to the Author)
In the revised manuscript the authors have addressed my previous comments satisfactorily.

Reviewer #3

(Remarks to the Author)
The authors have adequately addressed all comments. The manuscript is suitable for publication.

Reviewer #4

(Remarks to the Author)
The authors have made an excellent revision of the manuscript, addressing all my comments thoroughly.

Open Access This Peer Review File is licensed under a Creative Commons Attribution 4.0 International License, which permits use, sharing, adaptation, distribution and reproduction in any medium or format, as long as you give appropriate credit to the original author(s) and the source, provide a link to the Creative Commons license, and indicate if changes were made.
In cases where reviewers are anonymous, credit should be given to 'Anonymous Referee' and the source.
The images or other third party material in this Peer Review File are included in the article's Creative Commons license, unless indicated otherwise in a credit line to the material. If material is not included in the article's Creative Commons license and your intended use is not permitted by statutory regulation or exceeds the permitted use, you will need to obtain permission directly from the copyright holder.
To view a copy of this license, visit https://creativecommons.org/licenses/by/4.0/

Reviewer #1 (Remarks to the Author):

I co-reviewed this manuscript with one of the reviewers who provided the listed reports. This is part of the Nature Communications initiative to facilitate training in peer review and to provide appropriate recognition for Early Career Researchers who co-review manuscripts.

Reviewer #2 (Remarks to the Author):

Summary:
The authors present ADAPT-M (Affinity Determination by Adaptation of ProTein binders for Microfluidics), which combines an initial screening using yeast surface display (YSD) with quantitative evaluation of selected binders using the STAMMPPING microfluidic platform. The authors demonstrate the ability to measure affinity and dissociation kinetics for hundreds of enriched protein variants in a matter of days, without the need for traditional protein purification
This development is timely; with the increasingly rapid development of machine-learning methods for de novo binder generation, there is a growing demand for fast, high-throughput, and quantitative experimental validation methods. Several methods have been developed for this purpose, including TITE-seq, MAGMA-seq, and AlphaSeq, though these often remain labor-intensive or lack precise biophysical parameter measurement.
The authors optimize the method by selecting a monobody library against the Omicron SARS-CoV-2 RBD. They then validate the workflow by comparing in cellulo and in vitro affinities, and by characterizing two lead binders using cryo-EM and epitope/paratope mapping.

General Assessment:
Pérez et al. present ADAPT-M, a new method for fast and quantitative analysis of protein–protein interactions in enriched protein libraries. The approach combines the power of an initial yeast-display screening to enrich candidate binders with a second quantitative validation step using STAMPPING. The amount of work presented is substantial, and the experiments are carefully executed. Furthermore, the validation of the results using multiple orthogonal techniques convincingly demonstrates the robustness of the method for identifying protein binders. This combination of technologies is timely and valuable for the field, especially given the increasing demand for high-throughput binder discovery and kinetic characterization tools.
The writing is clear and the figures are of high quality. I recommend publication after the authors address a few minor points and clarify specific technical aspects. This will improve the article and it clearer and more accessible to readers.

Minor points
1. A new supplementary figure with information about the last purification step (SEC profile) and SDS-PAGE should be included in the paper to ensure reproducibility.
We thank the reviewer for their suggestion. In response, we have added Supplementary Fig. 27 which contains SEC profiles and SDS-PAGE gels for proteins purified in this study.

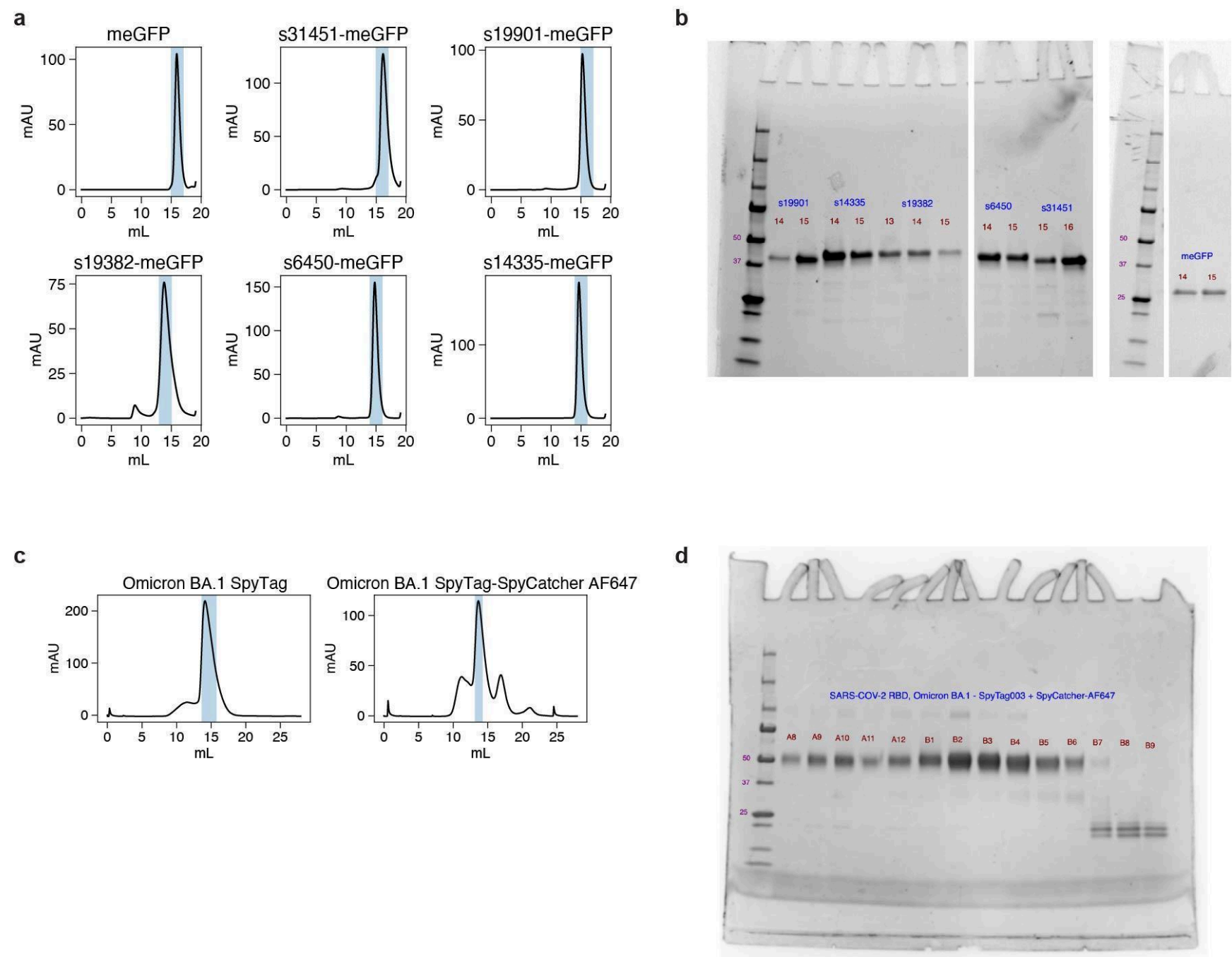

"**Supplementary Fig. S27**. SEC profiles (**a**, **c**) and corresponding SDS-PAGE gels (**b**, **d**) for SEC-purified monobodies and Omicron BA.1 RBD. Blue highlighted regions in SEC profiles correspond to fractions used for downstream experiments. SDS-PAGE was run under reducing conditions."

2. Data and text related to Figure 5. The authors perform epitope mapping of s19382 and s14335, using deep mutational scanning via yeast display. The results indicate that both selected monobodies for validation bind different epitopes than the ones used during the design and the ones predicted using AF3. Then, they use cryo-EM to solve the structure of RBD-bound monobody s19382, confirming these results. I think the authors make a good job with this validation workflow, but somehow the representation of the results is not good enough to give the reader a clear picture of the RBD epitope and how it compares to the residues identified by mutational analysis. I would suggest the authors to:

A. Include somewhere in the text, figures or supplementary figures, a list with the identified residues forming the RBD epitopes, and which amino acids forming this experimental epitope could be identified using mutational scanning.

We thank the reviewer for their suggestion and have included a list of residues forming the RBD epitopes in Supplementary Table S5 and Supplementary Table S6 for s19382-RBD and s14335-RBD, respectively. We bolded the s19382 residues that are validated by cryo-EM to highlight the correspondence.

Supplementary Table S5: RBD interface residues identified by design, mutational mapping, and cryo-EM models for s19382-RBD. Residues validated by cryo-EM are marked in blue **bold** text.

Design model	R408, T415, G416, N417, D420, Y421, K424, L455, F456, R457, K458, S459, N460, Y473, Q474, A475, G476, N477
Mutational mapping	A344, A352, N354, **D364**, **N370**, P373, **F377**, G381, **P384**, **T385**, K386,

	D389, G413, Q414, R466, S469, P499
Cryo-EM	A363, D364, Y365, S366, Y369, N370, K376, F377, C379, S383, P384, T385

Supplementary Table S6: RBD interface residues identified by design model and mutational mapping for s14335-RBD.

Design model	R403, D405, R408, Q409, T415, G416, N417, Y421, L455, F456, R457, Y473, A475, F486, N487, Y489, H505
Mutational mapping	A344, Y351, N354, R355, K356, D364, S366, N370, L371, A372, P373, F374, F375, T376, F377, K378, G381, R403, Q414, G416, N437, P463, F464, E465, R466, D467, P499

B. Panel D should be improved to give a clear picture of the RBD epitope and the monobody paratope, or at least to show the major amino acids involved in binding. Below we show an idea on how this part of the figure could look (just a suggestion using ChimeraX, see attached), and labels should be added to the main amino acids at the interface to make it easier for the reader to follow it.
We have updated Panel D as suggested.

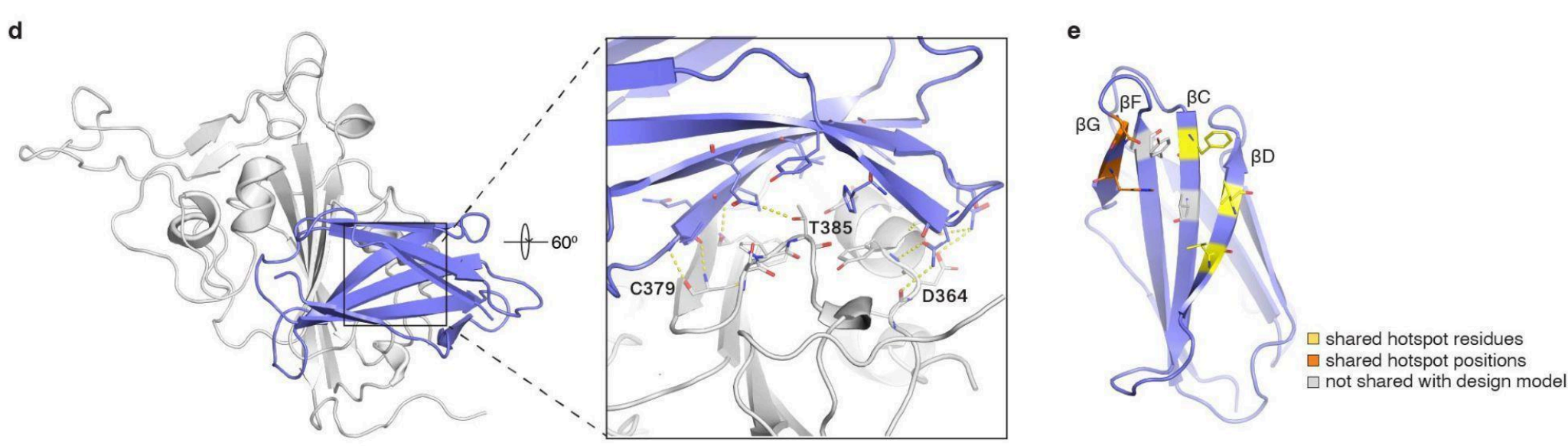

3. Figure S18 could be improved if each designed model again RBD would be represented in independent panels. As the two models are represented at the same time, there are chain clashes and could be confusing to the reader.
It would be clearer if its represented (A) s14335 binding to RBD and then (B) s19382 binding to RBD.
We thank the reviewer for their helpful suggestion and have updated Supplementary Fig. S18 accordingly.

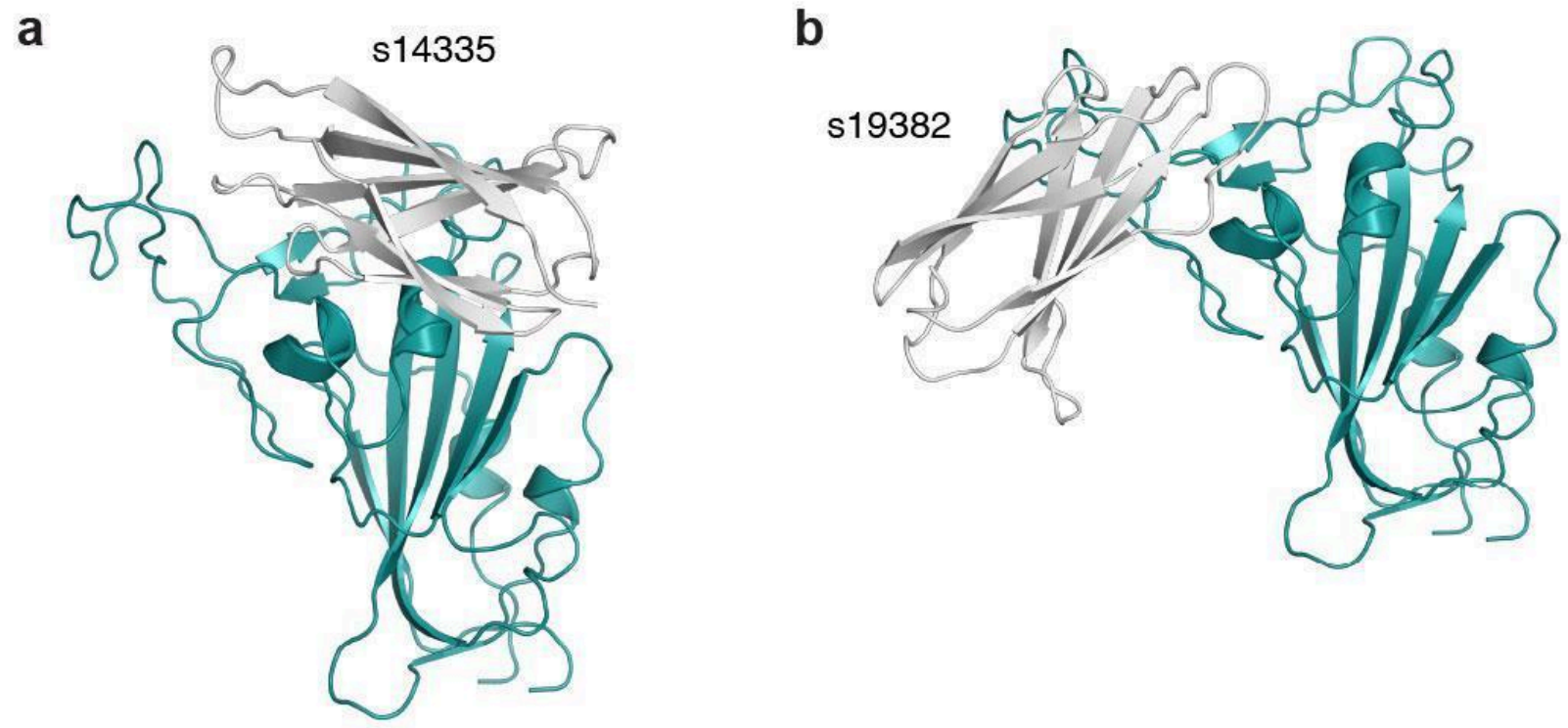

4. Figure S20 should be remade highlighting only (with a different color, for example) the amino acids that form the interface between the monobody and the RBD.
We thank the reviewer for their suggestions. As these changes have been incorporated into Fig. 5, panel D we have opted to remove Supplementary Fig. 20.

5. In figure S25, it mentions AlphaFold2 but probably it would be AlphaFold3?
We thank the reviewer for noting this error, and have updated the text to reflect the correct method, AlphaFold3.

6. More information about the sonication conditions could be given in the Materials and methods.
We thank the reviewer for this suggestion and have modified the text as follows:

Thawed pellets were sonicated on ice in a ~35mL volume over 12 minutes using 30s ON / 59s OFF pulses with a ¼" probe on a Q500 sonicator (Qsonica) operating at 20% power, ensuring the sample remained cold throughout sonication.

7. More detailed information about Ni-NTA and SEC purifications could be added to facilitate reproducibility.
We agree with the reviewer and have expanded the description of Ni-NTA and SEC procedures throughout the methods section. One such example under "SARS-CoV-2 Omicron/BA.1 protein expression and purification" is provided below:

Filtered supernatants were diluted 2-fold in ice-cold PBS and supplemented to a final 30mM imidazole concentration before being loaded onto a pre-packed nickel affinity column (HisPur resin, Thermo Fisher Scientific) pre-equilibrated with PBS containing 50mM imidazole. Resin was then washed with 10-20 column volumes of PBS containing 50mM imidazole before being eluted over 12 0.5mL fractions using PBS containing 500mM imidazole. Eluted fractions were analyzed by SDS-PAGE. Fractions containing protein of interest were pooled and concentrated using Amicon Ultra-4 10kDa MWCO centrifugal filters (Sigma Aldrich) to a 2mL volume. Concentrated protein was purified by size-exclusion chromatography (Superdex 75 Increase 10/300 GL or Superdex 200 Increase 10/300 GL, GE Healthcare) into PBS. SEC was performed at 4ºC on an ÄKTA chromatography system at a flow rate of 0.5 mL/min, and 1 mL of concentrated protein was injected per run. Elution was monitored by UV absorbance at 280 nm, and fractions corresponding to the major monodisperse peak were collected and analyzed by SDS–PAGE. Concentrations were determined by absorbance at 280 nm

measured with a NanoDrop8000 spectrophotometer (Thermo Fisher Scientific) using predicted extinction coefficients.

8. The authors state that AlphaFold3 predictions were unsuccessful. The use of AlphaFold3, even with the standard parameters, should be described in the methods.
We agree with the reviewer and have added a new methods subsection detailing AlphaFold3 structure predictions as follows:

"Structure prediction with AlphaFold3
Structure predictions were generated using model weights and code released by Google DeepMind and Isomorphic Labs in early November 2024. For structure prediction of the s19382-RBD complex, 1000 seeds were predicted. For structure prediction of the 33,095 Sculptor designs, a single seed was predicted per target. In each case, five decoys were generated per seed. Top scoring decoys across all seeds were selected for structure or metric analysis by taking the model(s) with the highest 'ranking_score'. Pre-computed MSAs and templates (6YOR, 7L0N, 7MFU, 7OAQ) were used for RBD, identified via structure prediction of RBD with genetic and template search. MSAs and templates were similarly obtained for s19382 in s19382-RBD structure prediction runs. For the single seed runs, the structures of 142 Sculptor-designed monobodies were first predicted using AlphaFold3 with genetic and template search enabled. Output MSAs were parsed to identify a set of 7250 unique Uniprot sequences, which were subsequently used to construct a reduced mmCIF database for monobody MSA generation and template search that significantly reduced genetic and template search times."

9. In Figure S19, a low pass filtered image of a micrograph could be shown to check particle distribution of the dataset as well as the projections from the 2D classes obtained in the 2D classification step.
The requested images have been added to Supplementary Fig. S19 b and c.

b

c

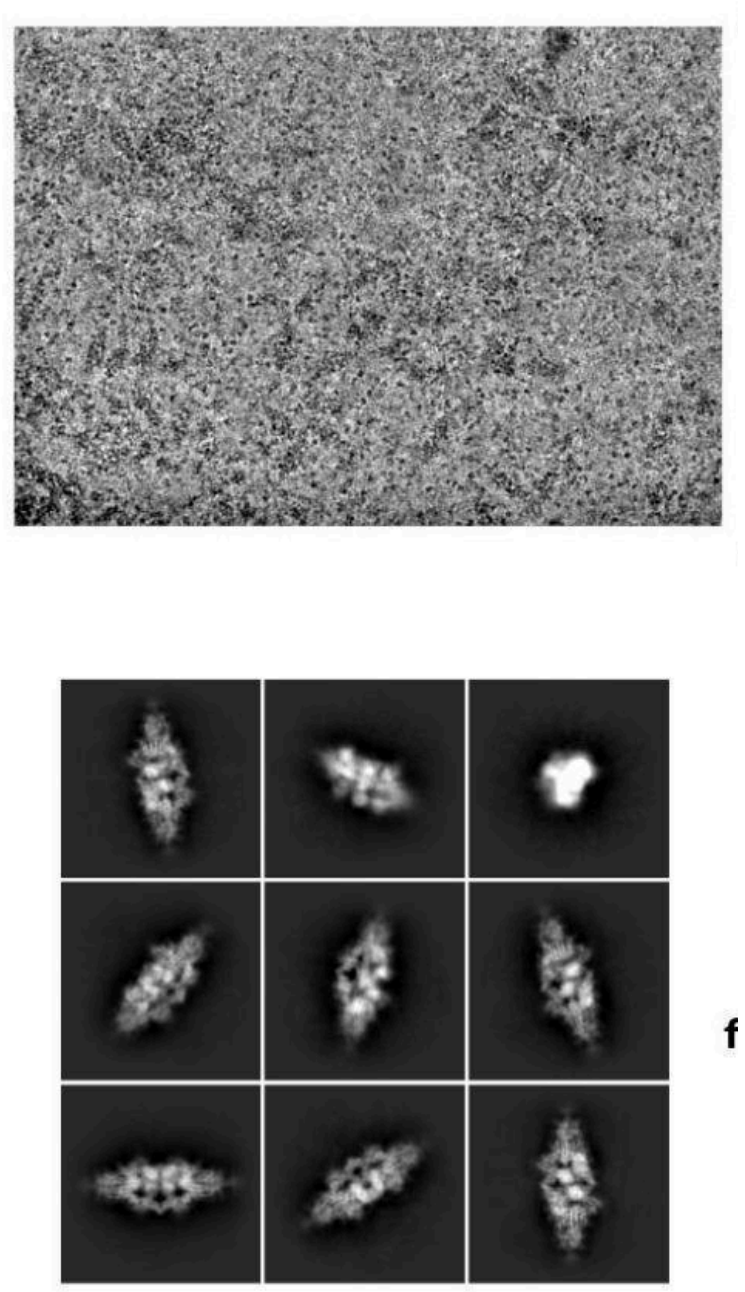

f

10. In Figure S19, panel e) add the resolution units (Å) to the colored bar, the units (Å) can also be added in the figure legend.]
Resolution units have been added as suggested.

Reviewer #3 (Remarks to the Author):

Review of NCOMMS-25-82922

“ADAPT-M: A workflow for rapid, quantitative in vitro measurements of enriched protein libraries” by Perez et al.

Summary:

Perez et al. introduce ADAPT-M, an integrated experimental workflow that connects yeast surface display-based enrichment with rapid microfluidic affinity measurements using their STAMMPPING platform. The authors demonstrate the approach by evolving monobodies against SARS-CoV-2 Omicron BA.1 RBD, assessing enriched variants without purification, validating selected hits with orthogonal biophysical assays, and presenting structural and mutational analyses of lead binders. The manuscript is clearly structured and technically well executed, and the demonstrated application is compelling. The following points should be addressed before publication:

1) While the authors repeatedly emphasize a “high-throughput” capability, the demonstrated quantitative characterization scale (33 variants after enrichment from an initial library of ~12,000) may not, in itself, constitute high throughput in terms of the measurement that is performed on the 33 variants. The throughput claims should be moderated and/or more clearly contextualized relative to existing, comparably scalable methods.
We agree with the reviewer, and in response, have removed all claims that ADAPT-M is high-throughput with the data presented here. In particular, we removed the words high-throughput in the abstract, results, and headings.

2) The discussion strongly highlights the limitations of alternative affinity-determination methods (ITC, BLI, etc.), yet provides comparatively little critical reflection on the limitations intrinsic to the ADAPT-M and STAMMPPING approach. Also, as a surface-based assay, effects such as surface heterogeneity, mass-transport limitations, avidity, and probe-density dependence should be acknowledged.
We agree with the reviewer, and in response, have added a paragraph to the discussion section to discuss the two main limitations to our approach: surface immobilization and use of fluorescent tags as follows:
As with other surface-based affinity assays, factors such as surface heterogeneity, density-dependent effects, avidity, and potential mass-transport limitations could influence ADAPT-M and STAMMPPING affinity and kinetic measurements. In particular, high surface densities may promote re-binding or multivalent interactions that deviate from 1:1 binding models. To mitigate these effects, our platform

allows deliberate tuning of surface density, our analyses are performed by comparing variants under similar surface conditions, and we included 'dark' competitor in dissociation assays to minimize rebinding.

ADAPT-M and STAMMPPING are also limited by factors intrinsic to all fluorescence-based assays. As with all assays that label ligands, the presence of the label may impact measured affinities and dissociation kinetics. In addition, the minimum quantifiable surface density of the immobilized protein (here, low nanomolar) defines the limit for strong interactions that can be measured without entering a titration regime, while the maximum concentration of the protein that is flowed on defines the limit for quantifying weak interactions. In the results presented here, STAMMPPING measurements for monovalent-labeled RBD correlated well with measurements made via label-free BLI (R^2 = 0.83), suggesting that monovalent tags do not substantially impact the affinities.

3) Broader adoption of ADAPT-M may be limited by platform accessibility, given the specialized microfluidic infrastructure and automation required. It would substantially support dissemination if the authors make microfluidic chip designs, operating parameters, and setup guidelines openly available and provide detailed instructions on how to setup and operate these measurements.
We completely agree with the reviewer and have already made the microfluidic wafer CAD design files, wafer fabrication protocol, and PDMS device fabrication protocol freely available on our website (www.fordycelab.com/microfluidic-design-files). CAD design files will additionally be deposited as an Open Science Framework. Our device setup and operation are detailed in the methods section and our analysis processing pipelines are freely available on our GitHub page.

4) The workflow does not extract quantitative kinetic constants (kon/koff) and appears limited to equilibrium binding measurements plus qualitative dissociation behaviour. The authors should explain the origin of this limitation, whether it is fundamental to the current architecture, and whether temperature-dependent measurements (and thermodynamic analyses) are feasible.
We agree with the reviewer, and in response, have added the following text to the Discussion section:

Finally, while ADAPT-M and STAMMPPING can, in principle, be used to quantify dissociation rate constants (k_{off}), the presence of multiple kinetic microstates in the RBD-monobody interactions studied here produced multi-phase dissociation behaviors (also visible in BLI measurements) that could not be reliably fit with a single exponential model. Since similar microfluidic devices have previously been used to quantify dissociation rate constants for DNA oligonucleotides bound to surface-immobilized transcription factor proteins[28–30], STAMMPPING should also be able to quantify these interactions for single-state dissociation of protein-protein interactions.

5) The authors should clarify the dynamic range realistically accessible with this format, including upper and lower measurable affinity limits, and whether very strong binders (low-nM or pM) or weak binders can be accurately resolved.
We agree with the reviewer, and in response, we have added a sentence describing the exact affinity range that was measured (64 nM - 10 µM) to the Discussion, "*In addition, the minimum quantifiable surface density of the immobilized protein (here, low nanomolar) defines the limit for strong interactions that can be measured without entering a titration regime, while the maximum concentration of the protein that is flowed on defines the limit for quantifying weak interactions.*"

6) Some false positives remain after YSD enrichment. It would be valuable to discuss why these persist mechanistically and whether adjustments in the enrichment workflow could mitigate them.
In response to the reviewer we have added the following to the Discussion section:

Despite YSD's scalability, false positives can persist post-selection due to non-specific binding at high analyte concentrations, multivalent avidity effects arising from surface display, and/or unexpected glycosylation events. In principle, these false positives can be reduced through adjustments to the enrichment workflow (e.g. lower analyte concentrations, negative selection with off-target analytes). ADAPT-M offers an orthogonal quantitative workflow that filters out false positives post-enrichment, enabling rapid discrimination between true high affinity binders and YSD artifacts.

7) Because quantification relies on display-linked readout, there may be a bias toward variants with higher expression rather than strictly higher affinity. It is worth clarifying whether low-expressor high-affinity clones could be underrepresented, and whether the platform can experimentally disentangle expression level from true affinity.
The reviewer is correct. Low expressor, high affinity clones may be underrepresented in the ADAPT-M workflow because the probability of sampling individual clones is dependent on expression levels in yeast. To some extent, this can be mitigated by enforcing large expression and affinity gates during sorting as is standard practice. Increasing sampling during the ADAPT-M workflow would also help increase the probability of sampling.

It is always important to perform NGS on input and output rounds of YSD selection. If enriched variants are identified by NGS but not by ADAPT-M, those sequences should be ordered and manually added before step 5 (when PCR amplification of linear IVTT templates occur). A key advantage of ADAPT-M is its ability to recover and simultaneously measure a greater number of sequences than would otherwise be feasible with traditional methods. We have updated the text as follows to reflect the importance of continuing to perform these QC steps:
"To assess the number of enriched sequences recovered by ADAPT-M, NGS sequencing analysis of input and enriched YSD populations was performed, revealing a recovery rate of 92% (33/36) for the 36 highly enriched sequences (Fig. 2d, Supplementary Table S3). This type of quality control is essential to ensure variants of interest are adequately represented by the ADAPT-M protocol and identify missing variants that can be ordered and screened separately or during replicate STAMMPPING measurements."

If successfully sampled, STAMMPPING can disentangle expression level from true affinity as binding responses are normalized by expression level.

8) Reproducibility across devices, days, and operators is not discussed or shown in depth. Presenting such assessment, even briefly, would increase confidence in robustness.
We agree with the reviewer. We have included device replicate data in the supplement and have clarified the main text to now read, "*Fitting fractional occupancies as a function of introduced Omicron RBD concentration to a quadratic binding equation yielded K_d values that were highly replicable across two devices (Pearson R^2=0.91, RMSLE= 0.64, **Fig. 2i, Supplemental Fig. 5**).*"

Reviewer #4 (Remarks to the Author):

The manuscript by Perez et. al. presents a new methodlogy for high throughput screening of protein-protein interactions using yeast suface display and a microfluidic platform. The proposed pipeline has the advantage of being capable of determining Kds in unpurified samples. In this case the authors use a library of computationally designed binders against the SARS-CoV-2 Omicron BA.1 protein to validate the methodology, though it holds the potential to screen other binder molecular such as VHHs.

Subject to minor revisions focused on clarity, scalability arguments, and cost considerations, the manuscript is suitable for publication.

Introduction and motivation
- Lines 50–52: SPR and BLI indeed have lower throughput in their standard formats, but high-throughput variants exist (automated 96-well or 384-well arrays). The manuscript should acknowledge these developments and clearly articulate the comparative advantages of ADAPT-M beyond scaling (e.g., cost per measurement, hands-on time, multiplexing capability).
We have revised the introduction, as the reviewer suggested, to acknowledge high-throughput SPR and BLI variants.

"Traditional in vitro approaches for binding quantification such as surface plasmon resonance[7] (SPR), biolayer interferometry[8,9] (BLI), or isothermal titration calorimetry[10] (ITC) have historically been low-throughput, typically requiring purified recombinant proteins which are often labor-intensive to produce. Recent advancements have improved the scalability of these techniques. For example, advancements in instrumentation have increased throughput by enabling larger numbers of samples to be measured in parallel (e.g. plate-based systems such as the Octet RED384 and Biacore 8k+), and coupling of these techniques to cell-free expression allows binding measurements to be performed directly on unpurified expression reactions[11,12]. However, these approaches require significant amounts of reagents (i.e. purified target protein to distribute across every well and expensive store-bought or challenging-to-prepare house-made cell-free reaction mixtures), and rely on gene synthesis of DNA templates for expression, which can be cost-prohibitive for large sequence libraries. As a result, only a subset of candidate sequences can be finely characterized, limiting insight into sequence-function relationships and the availability of quantitative affinity data for training predictive models."

We have additionally added Supplementary Table S2 directly comparing cost per measurement, hands-on time, and multiplexing capability of SPR and ADAPT-M.

Supplementary Table S2. Comparison of traditional binding affinity methods (SPR, BLI) with the ADAPT-M + STAMMPPING workflow presented in this study.

	SPR / BLI	ADAPT-M + STAMMPPING
Cost per measurement	$75.73/variant	$3.20/variant

Gene synthesis	Individual gene fragments need to be ordered	No gene synthesis required, produced in-house by primers that can be shared across samples
Antigen Consumption	High antigen usage per reaction, even at 384-well plate scale	Low antigen quantity supports 1,000s of measurements (variants & replicates)
CFPS	Reactions can be scaled down to as low as 15uL[1] but limited by low-volume pipetting rather than protein yield	50uL reaction-scale sufficient for full microfluidic device (up to 1800 measurements) Not limited by low-volume pipetting constraints
Time to Measurement	10 days	5 days
Ordering Lead Time	8 day lead time required for sequencing, analysis, and gene fragment delivery before experiments can begin	No ordering required; measurements can begin immediately from existing YSD output
Measurement Throughput	8 samples at a time, but can be automated	~24 hours for ~1,200 measurements (including 600 blanks)
Hands-on Time	~4 hrs	~12 hrs
User Intervention	Primarily during plate setup; SPR/BLI protocols are more automated with minimal user intervention during runs. No hands-on time for DNA generation (ordered).	More user touchpoints during measurement, but largely automated. PCR steps required for DNA generation.
Multiplexing Capability	Yes, but more limited.	Yes
Multi-Antigen Testing	Multiple antigens possible, but must be done serially with surface regeneration between each antigen	Up to 8 antigens on a single device
Diversity Trade-off	Adding antigens proportionally increases required instrument time	Additional antigens reduce per-antigen diversity, but parallel chip runs readily compensate
Binding Parameters	k_{on}, k_{off}, K_d	K_d
Kinetic Information	Full kinetic profiles (k_{on}, k_{off}, K_d) from a single experiment; gold standard for affinity	steady state K_ds, apparent k_{off}

	characterization	

- Line 55: The term neutralization is too broad in this context. Please specify whether the authors refer to measuring competitive inhibition, ligand blocking, epitope occlusion, or another operational definition within the ADAPT-M workflow.
Neutralization in this context is measured by competitive inhibition. However, in our reworking of the text to include mention of BLI and SPR, we have removed this sentence altogether.

- Lines 70–71: Only 14 binders ultimately yielded measurable Kd values above the detection threshold. Although the platform itself is scalable, the results provided do not yet demonstrate high-throughput capability compared to cell-free synthesis coupled with SPR/BLI, where tens to hundreds of binders can be screened routinely. The authors should temper the claim or provide quantitative rationale for throughput scaling (e.g., number of chambers, device multiplexing, total measurement capacity).
This point is similar to a point made by Reviewer #3. As mentioned above as well, we agree with both reviewers, and in response, have removed all claims that ADAPT-M is high-throughput with the data presented here. In particular, we removed the words high-throughput in the abstract, results, and headings.

- Line 88: Please specify the total number of days required for Stage 1. The text states 2–3 weeks for YSD enrichment, while line 93 indicates 3 weeks. A consolidated timeline per stage would improve clarity.
We thank the reviewer for pointing out this inconsistency. Yeast transformation followed by three rounds of sorting can typically be completed in 14-15 days. The previously stated 2-3 weeks was meant to reflect differences in scheduling (business weeks vs continuous daily work) as well as variability in the workflow. For example, slower yeast growth (either from growth at lower temperatures or from lower seeding densities after sorting) or additional rounds of selection may extend the timeline.

To improve clarity, and maintain consistency across the manuscript, we report the time required for Stage 1 as 14+ days, which provides a lower bound for the time required. We have updated the relevant text accordingly.

- Line 103: To strengthen the argument for high throughput, automated colony picking should be mentioned as a compatible option. Manual colony picking is not scalable and limits the practical utility of the pipeline.
Yes, we agree manual colony picking is not scalable for larger libraries. Most research labs have a FACS sorter, whereas many institutions do not have an automated colony picker. In the text, we mention that FACS sorting single bacteria into 384 well plates can provide a mode scalable method; we also include data in the supplement comparing the two approaches.

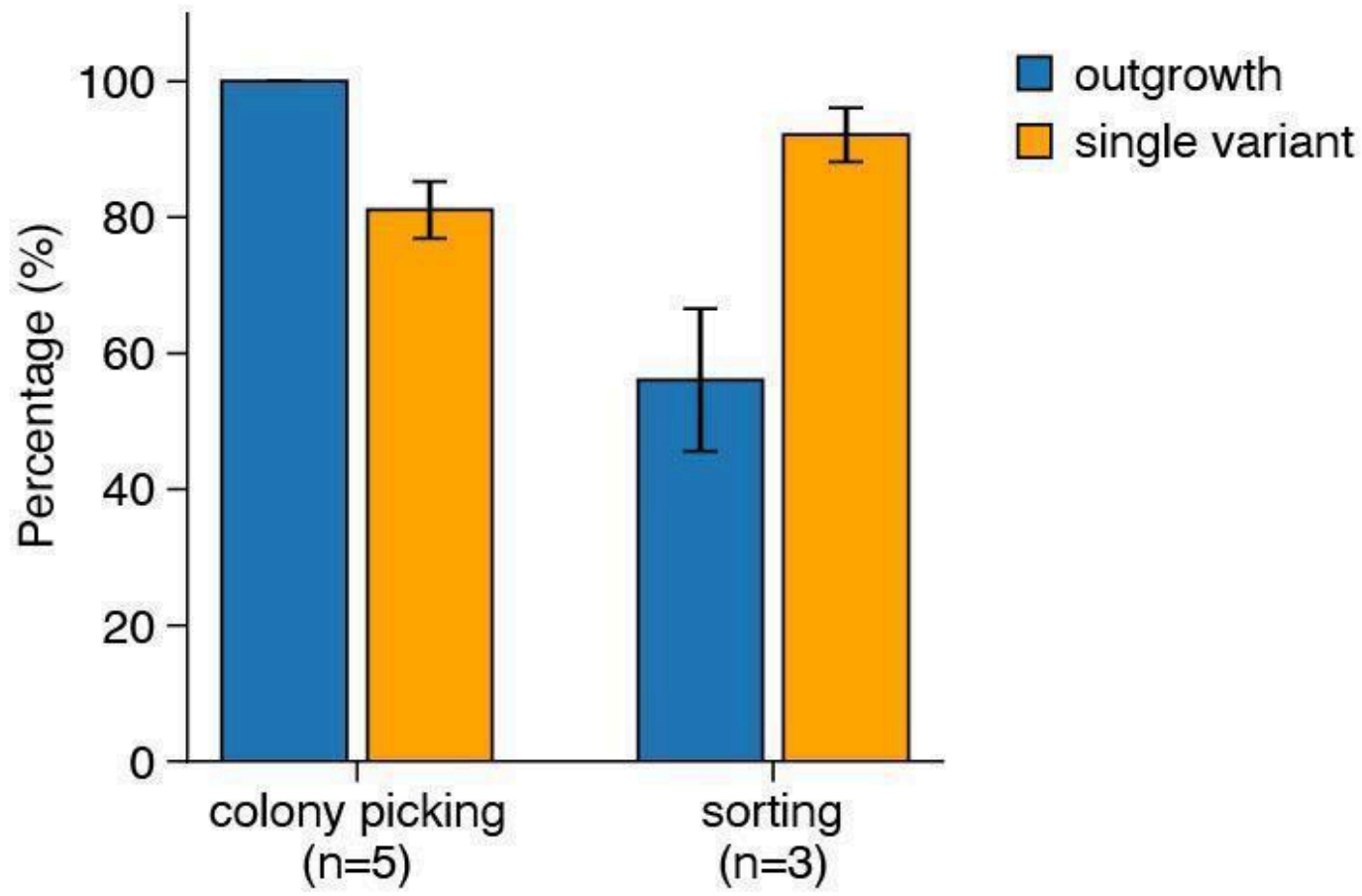

Supplementary Fig. S2. Bar plots showing cell outgrowth and single variant isolation rates for manual colony picking versus single cell sorting. Data represent mean values; error bars indicate standard error from biological replicates.

- Lines 109–110: The authors should comment on the number of colonies picked versus unique sequences obtained, and whether diversity loss occurred at this step. This is relevant for assessing bottlenecks in the pipeline.

Yes we include that information on lines 148-152. There we say, "*After individually inoculating 274 wells of a 384 well plate by hand, all inoculated wells showed successful outgrowth. Sequencing after STAMMPPING experiments revealed 51 unique monobody sequences, with a recovery rate of 92% (33/36) for the 36 highly enriched sequences (**Fig. 2d, Supplementary Table S3**).*"

Unlike with designed libraries where you know the exact number of unique library members, with yeast surface display, after enrichment, you do not know how many enriched sequences you will obtain. The number of enriched sequences depends on the number of rounds of sorting, as well as the gating strategy and the target's selectivity. We obtained 51 unique monobody sequences after picking 274 colonies. After performing NGS sequencing on the pool of enriched cells, as well as the input library, of those 51 monobody sequences, 33 sequences were highly enriched, where the remaining monobodies were likely weaker or nonspecific binders pulled out from yeast surface display. As stated above, of the 36 highly enriched monobodies in YSD, we recovered 33 with colony picking, resulting in a 92% recovery rate.

- Line 115: "Supplementary*".

We appreciate the reviewer's correction and the text has been modified throughout.

- Lines 117–118: Because the pipeline relies on capture via an anti-eGFP nanobody and uses microfluidic devices with patterned surfaces, the authors should provide an approximate cost per screened variant and indicate whether patterned slides or devices can be reused. For adoption as an HT methodology, cost efficiency is critical.

In response to the reviewer's point and the previous point on comparing SPR and ADAPT-M, we have added Supplementary Table S1 to provide an approximate cost estimate per screened variant.

Supplementary Table S1. Cost analysis for screening protein variants with either SPR or BLI versus our ADAPT-M + STAMMPPING strategy. Costs are based on screening 51 unique putative binders and exclude costs associated with replicate measurements, thereby representing an overestimate for ADAPT-M+STAMMPPING. CFPS costs were estimated using the commercially available PURExpress system.

Cost per screened variant	SPR / BLI	ADAPT-M + STAMMPPING
Gene synthesis	\$34.79	\$1.28
Antigen consumption (single cycle kinetics)	\$21.98	\$0.43
CFPS	\$18.96	\$1.24
Surface Patterning	NA	\$0.25
Total cost	\$75.73	\$3.20

Regarding device re-use, we have included the following sentence in the Methods section:
"*Although it is theoretically possible to re-use devices (i.e. test binding to a different target) after patterning, expression, and target dissociation, all STAMMPPING experiments here were performed from newly patterned and expressed devices.*"

- Line 132: Given the strongly methodological nature of the manuscript, please report the time required for oligo pool cloning and whether any QC step was performed to evaluate representation of the designed library in the YSD format. Recovery rate or dropout analysis would be useful.
We have detailed the protocol used for oligo pool cloning in the Methods section. Generation of sufficient YSD-compatible DNA from oligo pools can be completed in a single day. Because preparation of yeast cells for transformation requires multiple days, oligo pool cloning is typically performed in parallel with yeast preparation and therefore does not add any additional time to the overall workflow. To clarify this point we have amended the following sentence:
"Initial identification of candidate binders from libraries containing up to 1 x 107 members with YSD requires 2-3 weeks (Fig. 1b, left). During the first week, the library is cloned into a YSD-compatible vector and transformed into yeast cells as fusions to a surface anchor protein, Aga2, permitting surface display of the library upon induction. During weeks 2 and 3, the library is induced and yeast cells are subjected to iterative rounds of fluorescence activated cell sorting (FACS) to select and enrich a target-binding population."

Given the relatively small size of the library screened here (12,000 members) and the high efficiency of a typical yeast transformation (~10^7 transformants), we expected to recover the majority of library members and therefore did not perform any dedicated QC prior to selection.

We have amended Line 139-140 to report the recovery rate of sequences represented in YSD format:
"*To identify designs that bound the target RBD, we cloned the library into a YSD vector (11,995/12,000 sequences recovered) and performed 3 rounds of FACS and outgrowth (Figure 2b).*"

Discussion

- The manuscript should more explicitly discuss the mismatch between in-cellulo and in-vitro affinities. The strong correlation between STAMMPPING and BLI contrasts with the weaker correspondence to YSD titrations. This supports a known bias in YSD where epitope accessibility, multivalent avidity, and cell surface context can inflate apparent affinity. This should be acknowledged and framed as motivation for the ADAPT-M workflow.

This was also mentioned by Reviewer #3. We agree with both reviewers and have a sentence in the Results section as follows in response:

"Observed strong binding within YSD experiments could reflect extrinsic properties inherent to the YSD environment (*e.g.* multivalent avidity effects, high local target concentrations, nonspecific binding to the yeast cell wall, and unexpected glycosylation patterns), *motivating the use of ADAPT-M that decouples binding from variant expression and presentation on the cell surface.*"

We also added the following to the Discussion in response:

Despite YSD's scalability, false positives can persist post-selection due to non-specific selection at high analyte concentrations, multivalent avidity effects arising from surface display, and/or unexpected glycosylation events. In principle, these false positives can be reduced through adjustments to the enrichment workflow (e.g. lower analyte concentrations, negative selection with off-target analytes). ADAPT-M offers an orthogonal quantitative workflow that filters out false positives post-enrichment, enabling rapid discrimination between true high affinity binders and YSD artifacts.

Figure 1

- Panel a: The STAMMPPING diagram is visually dense. Enlarging or simplifying it would improve readability and support broader adoption of the method.

We have enlarged the STAMMPPING diagram in panel by >200%, and to further improve clarity, we updated panel b to focus on a single microfluidic chamber.

- Panel b: The caption refers to "meGFP binding to the VHH," which is misleading. This step corresponds to immobilization or capture, not binding. Rephrase accordingly.

We have modified the caption as follows to improve clarity:

"meGFP-tagged candidate binders are expressed within each chamber on-chip using PURExpress, a cell-free transcription-translation system, and immobilized via anti-eGFP nanobody-mediated capture for on-chip purification."

- Panel c: The methods imply expression in bacteria prior to STAMMPPING, not in a cell-free transcription–translation system. The backbone used should be clearly indicated to avoid confusion between IVTT and bacterial expression.

In response to this comment, the text has been modified throughout to more explicitly indicate the use of DNA prior to on-chip expression using a cell-free transcription-translation system.

"meGFP-tagged candidate binders are expressed within each chamber on-chip using PURExpress, a cell-free transcription-translation system, and immobilized via anti-eGFP nanobody-mediated capture for on-chip purification."

- Panel b–c: This is a picky detail so feel free to ignore. If the amplicon map is the same in both panels, the T7 promoter should be represented using the same color for consistency.

We appreciate this suggestion and have updated panel b accordingly.

Additional points

- Clarify whether PDMS–glass devices, once patterned, can be washed and reused. This has practical impact on throughput, cost, and reproducibility

That is a useful point to mention. We have included the following sentence in the Methods section: "*Although it is theoretically possible to re-use devices (i.e. test binding to a different target) after patterning, expression, and target dissociation, all STAMMPPING experiments here were performed from newly patterned and expressed devices.*"

- The authors use meGFP-only and empty chambers, but nonspecific interactions appear variable. They should comment on how variability in background binding affects Kd estimation at the lower affinity limit.

We agree with the reviewer, and have added a sentence in the Methods section, under the section titled 'Determination of limit of detection/noise floor' as follows:

"Importantly, using empty chambers to establish the limit of detection provides a strict binding threshold, as meGFP-only containing chambers consistently exhibited weaker nonspecific binding than empty chambers. *Because of this, binding affinities for binders near the noise floor are likely to be an overestimate.*"

- The global fitting approach presumes similar stoichiometry and surface presentation. The authors should briefly justify this assumption, especially since monobodies differ in expression levels and may exhibit different immobilization efficiencies.

We agree with the reviewer, and have amended the Methods section to justify our 1:1 stoichiometry assumption as follows:

Determination of STAMMPPING equilibrium binding constants

Considering the structural data show a 1:1 stoichiometry, we assume all binders/targets bind with the same stoichiometry.

We further clarify that we cull chambers in which the monobody did not express, leaving us with a narrower distribution of immobilized monobodies:

"Prior to fitting curves to determine binding affinities, we performed several quality control steps. First, we filtered out any chambers with GFP expression levels below the threshold set by considering blank chambers (mean + ten or more standard deviations). *This leaves us with a narrow distribution of GFP intensities (and densities) for chambers analyzed for binding. While there is some variation (< 2-fold) in chamber GFP fluorescence within a given experiment, this has a very small effect on fit K_d values (**Supplementary Fig. 25**).*"

- The compressed dynamic range observed when RBD is labeled via the His-Avi/avidin system deserves a brief mechanistic interpretation (e.g., avidity, probe geometry). A short cautionary note in the discussion would guide users applying ADAPT-M to different targets.

Similar to Reviewer #3's point above, we have addressed both reviewers' points with an additional limitations paragraph in the Discussion section as follows:

Second, as a fluorescence-based assay, ADAPT-M and STAMMPPING are subject to limitations, similar to other methods that rely on labeling, including dynamic range constraints and deviation from physiological measurements from the addition of tags, which can impact the sensitivity and precision, as we observed here with tetrameric-labeled RBD. Comparison of STAMMPPING measurements for

monovalent-labeled RBD with label-free BLI yields a correlation of 0.83 R^2, suggesting the addition of monovalent tags do not substantially impact the affinities.
